# Direct synthesis of molecularly imprinted nanozymes with a biomimetic catalytic motif for glycosidic bond cleavage

**DOI:** 10.3389/fchem.2026.1866827

**Published:** 2026-08-11

**Authors:** Mohan Lakavathu, Yan Zhao

**Affiliations:** Department of Chemistry, Iowa State University, Ames, IA, United States

**Keywords:** artificial enzyme, biomass conversion, cellulose, cross-linking, micelle, molecular imprinting

## Abstract

Carbohydrates are the most abundant organic molecules on Earth and play essential roles in biology. Natural glycosidases cleave glycosidic linkages using a pair of conserved carboxylic acid residues. Here we report the direct synthesis of nanozymes that mimic glycosidases through molecular imprinting of thiosemicarbazide-derived glycan templates, prepared in one step from unprotected mono- or oligosaccharides. The thiosemicarbazide group binds strongly to a dicarboxylic acid functional monomer (FM), enabling installation of the double-acid catalytic motif into the imprinted active site. The resulting nanozymes cleave glycosidic bonds in oligo- and polysaccharides (e.g., cellobiose and cellulose) by a mechanism analogous to retaining glycosidases. Molecular imprinting also allows the active site to accommodate a single or predefined number of sugar residues, tuning both the size of the pocket and the distance between binding and catalytic groups, thereby enabling rational control of the cleavage site along the substrate.

## 1 Introduction

Enzymes excel at using functional groups with medium or even low intrinsic reactivities to catalyze challenging chemical transformations. Glycosidases, among the most proficient enzymes in nature (Wolfenden et al., 1998), cleave the glycosidic bonds in oligo- or polysaccharides with simply a pair of carboxylic acids (Lairson and Withers, 2004; Zechel and Withers, 2000). Cooperativity between the two acids is key to their success; removing one of the two acids from the active site can reduce the efficiency of the enzyme by as much as 100,000-fold (MacLeod et al., 1994; MacLeod et al., 1996).

Inspired by this remarkable proficiency and motivated by the desire to replace fragile biocatalysts with more robust materials, chemists have pursued a variety of platforms to build artificial enzymes, i.e., synthetic catalysts that replicate selected features of natural enzymes (Breslow, 2005; Kirby and Hollfelder, 2009; Raynal et al., 2014). In recent years, nanozymes, often with an inorganic core, emerged as a powerful class of enzyme mimics, especially those with oxidase activities (Huang et al., 2019; Jiang et al., 2019; Wang et al., 2018; Wang et al., 2019). One of the major challenges in the development of nanozymes is in the construction of substrate-tailored active sites. In natural enzymes, the active site is a multifunctional nanospace formed by protein folding and decorated with precisely positioned binding and catalytic groups. Its size, shape, and polarity are optimized for the intended catalysis. In contrast, chemists are generally much more adept at synthesizing molecules than engineering such complex, multifunctional active sites, which require precise control over three-dimensional structure and the placement of multiple functional groups within a molecular scaffold.

Molecular imprinting is probably the most efficient way to build a multifunctional 3D binding site custom-tailored for different molecules (Haupt and Mosbach, 2000; Wulff, 2002; Ye and Mosbach, 2008). The method typically involves formation of a highly cross-linked polymer network in the presence of template molecules and functional monomers (FMs) that bind the templates by noncovalent or reversible covalent bonds. If the template–FM complex can be captured successfully by the polymerization and cross-linking, removal of the templates from the polymer network creates imprinted pockets with size, shape, and distribution of functional groups potentially complementary to those of the templates. Traditional molecularly imprinted polymers (MIPs) face challenges such as insolubility, heterogeneous distributions of the imprinted binding sites, and possible template entrapment within heavily cross-linked networks. Nonetheless, the conceptual simplicity, straightforward synthesis, and broad applicability of MIPs make them useful in chemical and biological applications (Pan et al., 2018; Zhang, 2020). If catalytic groups can be installed near the imprinted binding sites, enzyme-mimetic MIP catalysts may be produced (Chen et al., 2010; Kirsch et al., 2009; Muratsugu et al., 2020; Servant et al., 2011; Shen et al., 2016; Wulff and Liu, 2012; Yuan et al., 2018).

In this paper, we report the direct synthesis of organic nanozymes for the hydrolysis of glycosidic bonds using cellulose as the substrate (Lakavathu and Zhao, 2025; Lakavathu and Zhao, 2026; Zangiabadi and Zhao, 2022). Cellulose makes up 35%–50% of the lignocellulosic biomass and represents an enormous natural resource for carbon-neutral, sustainable chemicals, fuels, and materials (Deng et al., 2023; Jing et al., 2019; Luterbacher et al., 2014; Osman et al., 2021; Robertson et al., 2017; Zhang et al., 2017a). The 1,4-glycosidic bonds in cellulose as acetals are in principle readily hydrolyzed under acidic conditions, but the high crystallinity of cellulose makes the polymer recalcitrant even to enzyme catalysts. Our cellulase-mimicking nanozymes are prepared directly in a 2-day, one-pot reaction from readily synthesized starting materials, made possible by the strong binding interaction between a double acid FM and the thiosemicarbazide-derived glycan templates. Our catalysts bind to a cellulose chain on the nonreducing end through reversible boronate bonds and employ a pair of carboxylic acids as in natural cellulase to cleave the polysaccharide. By controlling the distance between the binding and catalytic groups, we could tune the site of cleavage along the polymer substrate, to produce cello-oligomers of predefined chain lengths.

## 2 Results and discussion

**SCHEME 1:**
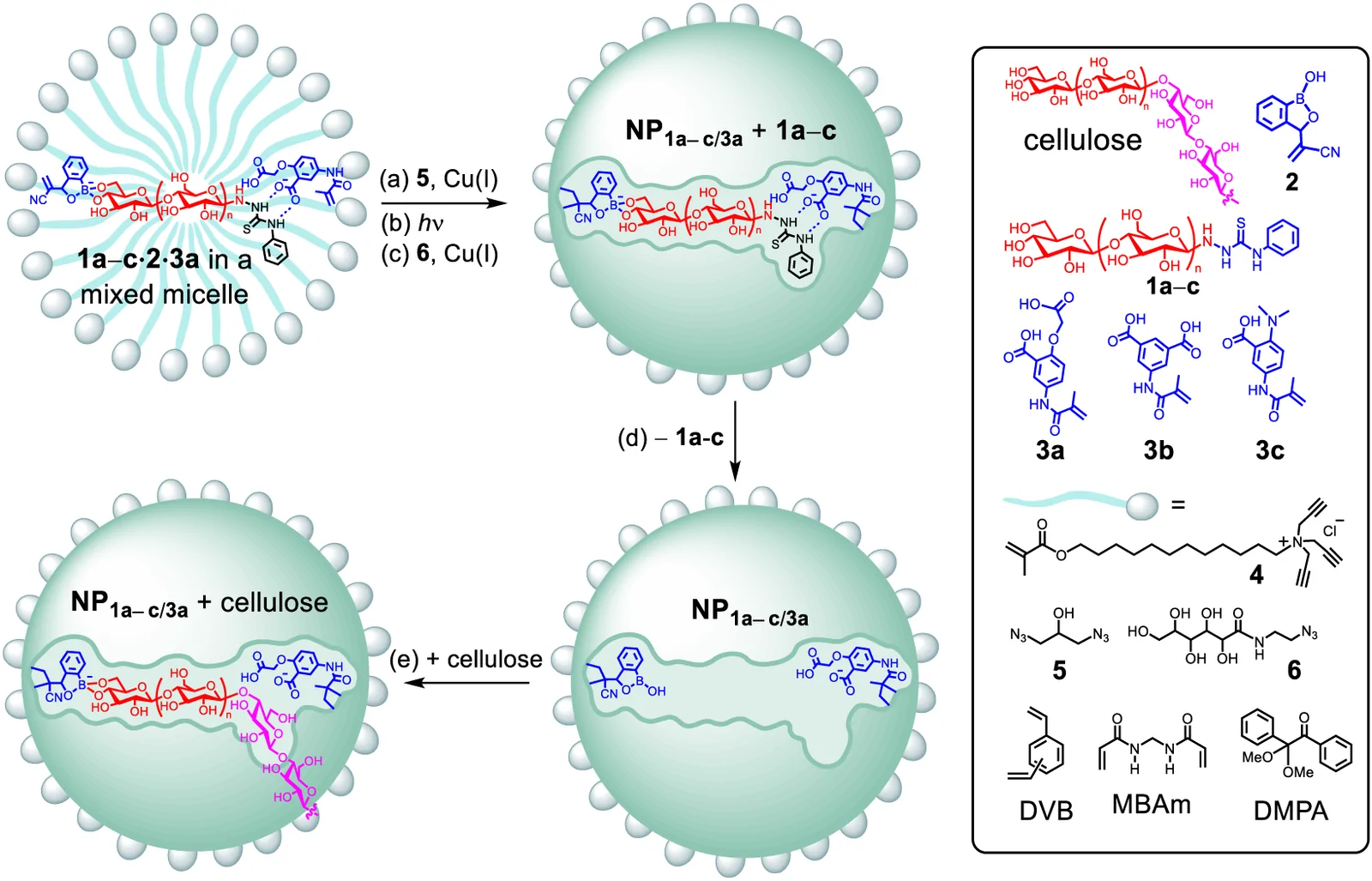
Preparation of nanozyme **NP**_**1a–c/3a**_ via molecular imprinting of **T**–**FM** complex within the cross-linkable micelle of **4**. The clicked surface ligands **6** are omitted from the drawing for clarity.

Scheme 1 illustrates our method to prepare synthetic mimics of cellulase, through molecular imprinting within cross-linkable micelles (Bose et al., 2023). The template molecules, which mimic a portion of the polysaccharide substrate, are thiosemicarbazide derivatives **1a**–**c**. These molecules were prepared in a one-step reaction from commercially available 4-phenylthiosemicarbazide and glucose, cellobiose, and cello-oligomers, respectively. The thiosemicarbazide group resembles thiourea structurally, which forms strong double hydrogen bonds with a carboxylic acid (Blažek Bregović et al., 2015). The template thus should interact strongly with carboxylic acid-containing FM **3a**–**c**. Another FM used in the imprinting is boroxole **2**, prepared via a one-step reaction from commercially available 2-formylphenylboronic acid and acrylonitrile (Sravan et al., 2013). Boroxoles are known to form reversible boronate bonds with the 4,6-diol of glucose even in water (Bérubé et al., 2008). Thus, we expect a termolecular T-FM complex **1a–c·2·3** to be formed in the micelle. Formation of hydrogen-bonded complexes in water is often challenging because of solvent competition. In our case, the template and FMs being amphiphilic are easily incorporated into the micelles of surfactant **4**. The nonpolar microenvironment of the micelle stabilizes the hydrogen-bonded complex (Duan and Zhao, 2021) and boronate formation (Gunasekara and Zhao, 2017). Additionally, incorporation of **1a–c**, **2**, and **3** into the micelles increases their local concentrations, further facilitating the associative process.

Molecular imprinting takes place in a three-step, one-pot reaction (Scheme 1): step a involves surface-cross-linking of the micelles with diazide **5** via the copper-catalyzed alkyne–azide click reaction; step b involves core-cross-linking via the photo-initiated free radical polymerization using DVB and MBAm as the oil- and water-soluble cross-linkers, respectively; step c is the surface-functionalization of the micelles by the click reaction of monoazide **6** with the residual alkyne groups on the micelle surface. Surface functionalization puts a layer of the sugar-derived ligands on the resulting molecularly imprinted nanoparticles (MINPs) and facilitates their purification by precipitation from acetone and solvent washing. **NP**_**1a–c/3a**_ denotes MINP prepared with **1a–c** as the template and **3a** as the acid-containing FM.

As shown in Scheme 1, FM **3a** has two carboxylic acids arranged ortho to each other on a benzene scaffold. The idea is that one acid will hydrogen-bond with the N–H bonds of the thiosemicarbazide group of the template, which has a basic nitrogen—i.e., the one bonded to the anomeric carbon of the glycan—that can potentially engage in ion-pairing/hydrogen-bonding interactions with the other carboxylic acid of **3a** (see the structure of T-FM complex **1a–c·2·3** in Scheme 1). In this way, a biomimetic double carboxylic acid motif is directly introduced via the polymerized FM into the imprinted site, near the glycosidic oxygen of the to-be-bound substrate, for cleaving the glycosidic bond.

**FIGURE 1:**
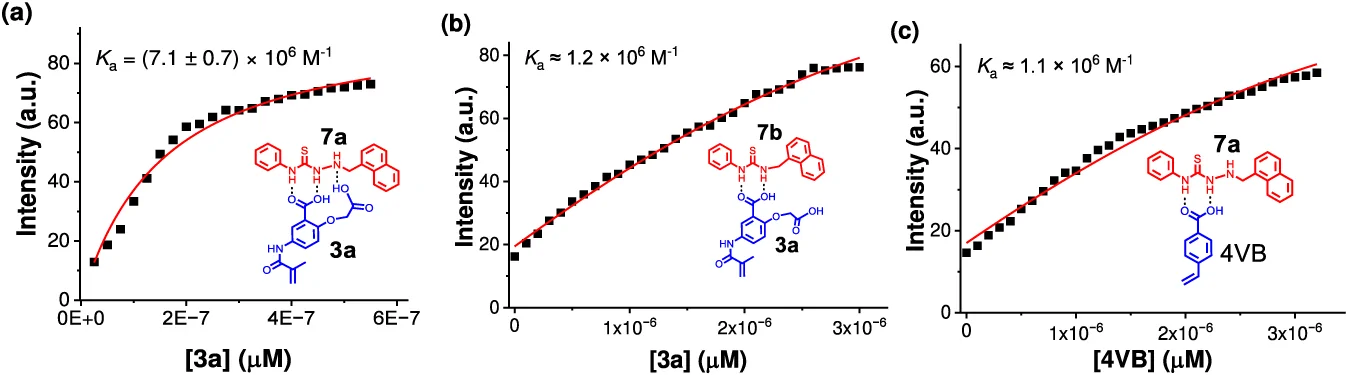
**(a)** Fluorescence intensity of **7a** as a function of the concentration of **3a** in 2 mM aqueous CTAB solution at 25 °C. **(b)** Fluorescence intensity of **7b** as a function of the concentration of **3a** in 2 mM aqueous CTAB solution at 25 °C. **(c)** Fluorescence intensity of **7a** as a function of the concentration of 4-vinylbenzoic acid (4VB) in 2 mM aqueous CTAB solution at 25 °C. [**7a**] = 1.0 nM; [**7b**] = 2.0 nM. The red smooth line is from the nonlinear least-squares curve-fitting of the fluorescence data to a 1:1 binding isotherm, with the binding constant (*K*_a_) given.

We previously verified the effectiveness of boroxole as a sugar-binding FM (Gunasekara and Zhao, 2017). To confirm the formation of the hydrogen-bonded complex between FM **3a** and the thiosemicarbazide template in micelles, we synthesized a model template **7a** (see its structure in Figure 1a). Its fluorescence allows us to study the template–FM complexation in non-cross-linkable cationic micelles of cetyltrimethylammonium bromide (CTAB), to mimic the situation during the micellar imprinting. We also synthesized thiourea analogue **7b** as the control (see the structure in Figure 1b). Note that **7b** does not have the basic hydrazine nitrogen of **7a**, so that it can only hydrogen-bond with one of the carboxylic acids of **3a**.

Figure 1a shows nonlinear least squares curve fitting of the fluorescence titration data of **7a** by **3a** to a 1:1 binding model. A binding constant of *K*_a_ = (7.1 ± 0.7) × 10^6^ M^-1^ is obtained, confirming the strong interaction between the template and the FM in the micelle environment. The binding mode does not impose regiochemical control over the two carboxylic acids of **3a**. If one acid forms double hydrogen bonds with the thiourea-like portion of the thiosemicarbazide group, the other may interact with the basic hydrazine nitrogen.

The control experiments support the double‐acid binding hypothesized in **7a·3a**. For example, without the basic hydrazine nitrogen, **7b** binds the same FM (**3a**) with a much lower *K*_a_ of ∼1.2 × 10^6^ M^-1^, confirming the importance of the hydrazine nitrogen to the binding (Figure 1b). The double acid on **3a** is also critical, as the binding between **7a** and monoacidic 4-vinylbenzoic acid (4VB) is similarly weak, with *K*_a_ ≈ 1.1 × 10^6^ M^-1^ (Figure 1c).

Having confirmed the strong double acid binding between model template **7a** and FM **3a** in micelles, we prepared **NP**_**1a–c/3a**_ following procedures illustrated in Scheme 1. To test the design hypothesis, we also prepared control MINPs using **3b** or **3c** as the functional monomer. FM **3b** has a meta geometry for the two acids and can help us understand whether the ortho relationship of the acids in **3a** is important. FM **3c** has a single carboxylic acid and a neighboring dimethylamino group. In natural glycosidases, one carboxylic acid in the active site acts as a general acid to help the departure of the leaving group, while the other one, in the deprotonated carboxylate form, acts as a general base or attacks the anomeric carbon directly as the nucleophile (Lairson and Withers, 2004; Zechel and Withers, 2000). It is possible that a carboxylic acid/amine dyad might work similarly.

Formation of the nanoparticles was monitored by ^1^H NMR spectroscopy, dynamic light scattering (DLS), and transmission electron microscopy (TEM) (see details in the Supplementary Material). To characterize the imprinted sites, we determined the binding constants (*K*_*a*_) of the nanoparticles for glucose, cellobiose, and cello-oligomers by isothermal titration calorimetry (ITC).

Due to strong hydration of carbohydrates by water molecules, their molecular recognition in water is extremely challenging (Zhao, 2022). Even their natural receptors (i.e., lectins) could only bind typical monosaccharides with a *K*_*a*_ value in the range of 10^3^–10^4^ M^-1^, much lower than those for typical drug molecules of similar size by their receptors (Kamerling and Boons, 2007; Wang and Boons, 2011). Table 1 shows that the nonimprinted nanoparticles (NINPs) prepared without any templates display negligible binding for glucose. Meanwhile, **NP**_**1a/3a**_ prepared with the glucose-derived template **1a** binds the monosaccharide with an impressive *K*_a_ of 14.8 × 10^4^ M^-1^, 1–2 orders of magnitude higher than those by typical lectins. Since benzoboroxole itself only binds glucose with a *K*_a_ of ∼30 M^-1^ in water (Bérubé et al., 2008), the 5000-fold increase in binding is a strong indicator for the successful imprinting of the sugar-derived template.

**TABLE 1:** ITC binding data for sugar guests by MINPs^a^.

Entry	Host	Guest	*K*_a_ (×10^4^ M^-1^)	Δ*G* (kcal/mol)	*N*
1	NINP	Glucose	-	-	-
2	**NP**_**1a/3a**_	Glucose	14.8 ± 1.2	−7.05	1.23 ± 0.04
3	**NP**_**1a/3a**_	Cellobiose	13.4 ± 1.6	−6.99	1.28 ± 0.03
4	**NP**_**1a/3a**_	Cello-oligomers	7.53 ± 0.25	−6.64	1.39 ± 0.01
5	**NP**_**1b/3a**_	Glucose	1.37 ± 0.16	−5.64	0.93 ± 0.12
6	**NP**_**1b/3a**_	Cellobiose	26.5 ± 3.0	−7.41	0.88 ± 0.05
7	**NP**_**1b/3a**_	Cello-oligomers	19.9 ± 1.0	−6.94	1.08 ± 0.01
8	**NP**_**1c/3a**_	Glucose	2.06 ± 0.17	−5.89	1.55 ± 0.06
9	**NP**_**1c/3a**_	Cellobiose	4.87 ± 0.41	−6.39	1.23 ± 0.04
10	**NP**_**1c/3a**_	Cello-oligomers	33.6 ± 3.2	−7.52	1.21 ± 0.02

^a^ The cross-linkable surfactants were a 3:2 mixture of **4** and **5**. The titrations were performed in 10 mM MES, buffer at pH 5 at 298 K.

During micellar imprinting, all the templates are amphiphilic and thus prefer to reside near the surface of the micelles. Molecular imprinting is expected to create imprinted pockets near the micellar surface as a result. Such a location should enable the imprinted site to bind not only the corresponding monosaccharide, but also an oligosaccharide or polysaccharide, as long as the carbohydrate could interact with the boroxole binding group in the imprinted pocket. As shown in Scheme 1, this binding motif is key to the desired hydrolysis of cellulose, as the sugar residues not engaging in the boronate bond formation stay outside the imprinted site, solvated by water.

Consistent with the binding model described above, **NP**_**1a/3a**_ binds not only glucose—its intended guest—but also cellobiose and cello-oligomers with similarly high affinity. Although these larger oligosaccharides cannot be fully accommodated within the monosaccharide-imprinted cavity, their binding affinities are only modestly reduced (Table 1, entries 2–4). The binding trends observed for **NP**_**1b/3a**_ and **NP**_**1c/3a**_ are likewise reasonable (entries 5–10): each nanoparticle preferentially binds its own templating sugar (cellobiose or cello-oligomers, respectively) over non-templating sugars. Notably, the cellobiose-imprinted **NP**_**1b/3a**_ also binds longer cello-oligomers effectively, similar to **NP**_**1a/3a**_, which recognizes both cellobiose and the higher oligosaccharides.

Interestingly, for **NP**_**1b/3a**_ and **NP**_**1c/3a**_, the binding of a shorter sugar—glucose for **NP**_**1b/3a**_ and glucose/cellobiose for **NP**_**1c/3a**_—is much weaker than for its own template, by more than an order of magnitude. Clearly, incomplete filling of the imprinted site is less favorable than overfilling (with the extra sugar units hanging outside the imprinted pocket as described above). The molecular surface of a carbohydrate comprises many small amphiphilic parts and is considered “polyamphiphilic” (Spohr et al., 1992). Water molecules near such a surface are known to have higher free energy than those in the bulk. Release of these high-energy water molecules is a major driving force for the binding between a glycan and a glycan-binding protein (Lemieux, 1996). Binding of a single glucose by hexokinase, for example, is estimated to release 65 ± 10 water molecules (Rand et al., 1993). In our case, sugar-binding by the molecularly imprinted nanoparticles is driven by a combination of covalent (i.e., boronate bond formation) and noncovalent interactions. Release of high-energy water molecules will certainly favor binding of a long guest that completely fills the binding pocket than a short one that only fills part of the pocket.

Most importantly, not only does **NP**_**1a/3a**_ bind cellobiose strongly (Table 1, entry 3), it is also able to cleave the disaccharide with enzyme-like Michaelis-Menten kinetics when the solution is heated. The catalytic turn-over number (*k*_cat_) for cellobiose at pH 6.5 and 90 °C is 0.047 min^-1^ and the Michaelis constant (*K*_M_) 493 ± 99 μM, affording a catalytic efficiency (*k*_cat_/*K*_M_) of 95 M^-1^ min^-1^. This efficiency is about 1/19 of that of the digestive β-glucosidase GH1 from *Spodoptera frugiperda* (1780 M^-1^ min^-1^) (Tamaki et al., 2016), quite impressive for a nanozyme prepared in a one-pot reaction.

**FIGURE 2:**
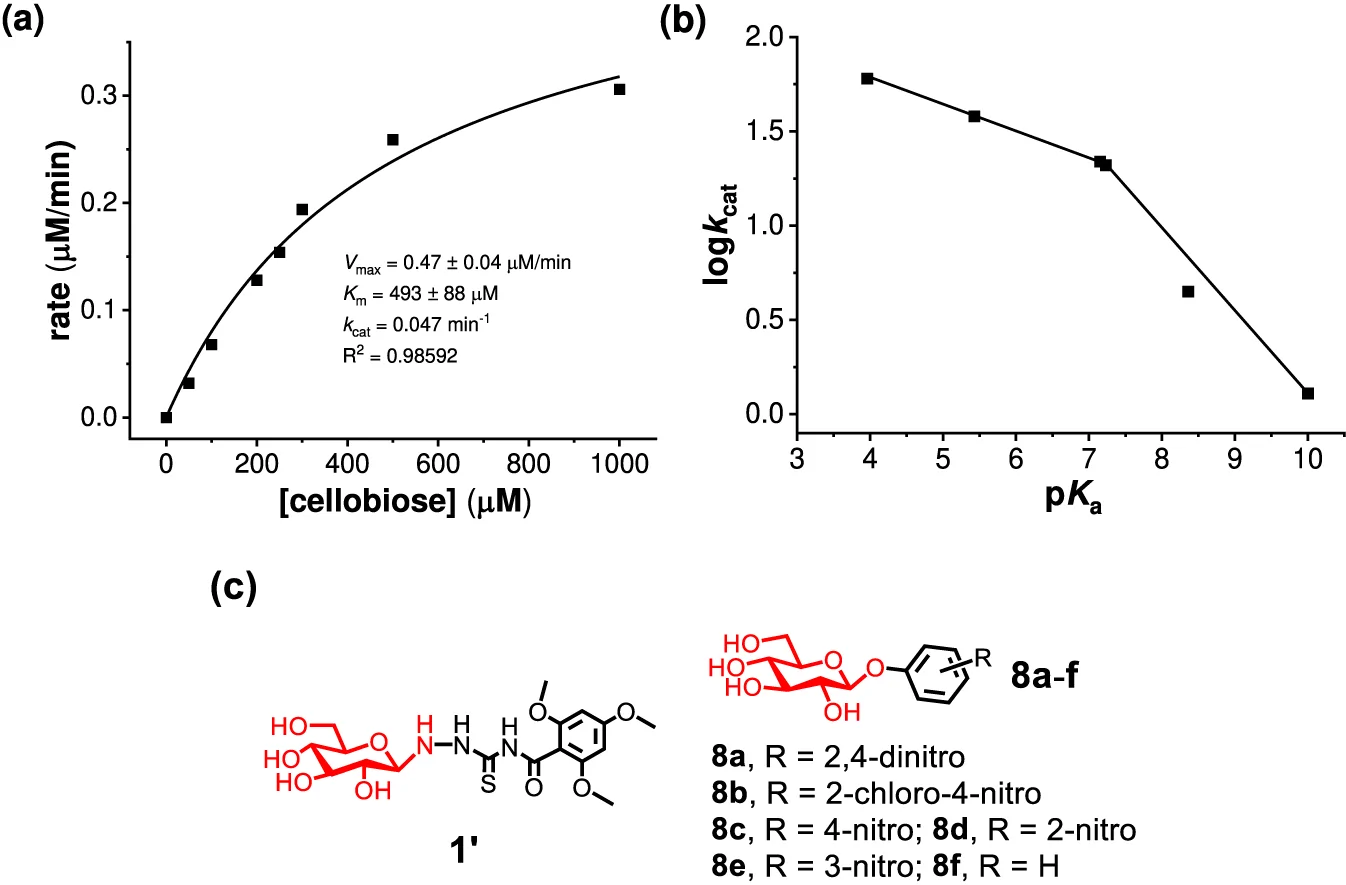
**(a)** Michaelis–Menten plot for cellobiose hydrolysis by **NP**_**1a/3a**_ in 10 mM MES buffer (pH 6.5) at 90 °C. [**NP**_**1a/3a**_] = 10.0 µM; **(b)** Brønsted plot for the catalytic hydrolysis of **8a**–**f** by **NP**_**1a′/3a**_ (prepared following similar procedures as in Scheme 1 using template **1a′**), plotted as a function of the p*K*_a_ value of the phenol leaving group. **(c)** Structure of template **1a′** and substrates **8a**–**f** for **NP**_**1a′/3a**_.

To probe the catalytic mechanism, we prepared **NP**_**1a′/3a**_ using template **1′**that has a bulky 2,4,6-trimethoxyphenyl group. The expectation is that the resulting active site will be able to accommodate aryl β-D-glucopyranosides **8a**–**f**. We then measured the Michaelis-Menten plots for all the catalytic hydrolyses of these substrates and obtained the Brønsted plot that correlates the log*k*_cat_ values to the p*K*_a_ of the phenol leaving groups in these substrates (Figure 2b). The plot obtained is similar to those reported for retaining glycosidases such as *Agrobacterium* sp. *β*-glucosidase, suggesting a similar catalytic mechanism (Kempton and Withers, 1992; Zechel and Withers, 2000). The biphasic plot consists of a less leaving-group-dependent region (p*K*_a_ = 4–7) and a more leaving-group-dependent region (p*K*_a_ = 7–10), due to the change of the rate-limiting step during the hydrolysis as a function of the leaving group.

Encouraged by the catalytic hydrolysis of cellobiose and aryl glucosides, we studied the hydrolysis of cellulose following established procedures to measure the “enzyme activity” of these catalysts (Brogan et al., 2018). The positive control was a commercial cellulase from *Aspergillus niger*, an endocellulase capable of cleaving both cellulose and cello-oligomers (Okada, 1988). The negative control was the NINP that showed negligible activity. The substrate was type 20 Sigmacell cellulose and the spectroscopic assay determined the total concentration of all reducing sugars as “glucose equivalents” (Brogan et al., 2018; Jurick, 2012).

**FIGURE 3:**
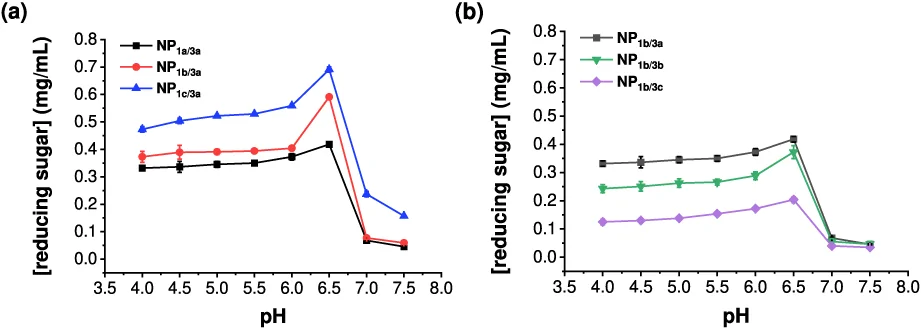
**(a)** Effects of solution pH on the hydrolysis of cellulose after 12 h at 90 °C by NP catalysts prepared with templates having different glycans (**1a**–**c**); **(b)** Effects of solution pH on the hydrolysis of cellulose after 12 h at 90 °C by NP catalysts prepared with **1b** as the template and **3a**–**c** as the catalytic FM. [Sigmacell cellulose] = 5.0 mg/mL. [catalyst] = 2.0 mg/mL. NaOAc buffer was used for pH 4.0–5.0, MES buffer for pH 5.5–6.5, and HEPES buffer for pH 7.0–7.4.

Figure 3a shows that **NP**_**1a–c/3a**_ all display a similar pH profile in its catalysis, with the fastest hydrolysis of cellulose occurring near neutral conditions, at pH 6.5. Among the three catalysts, the catalytic activity follows the order of **NP**_**1c/3a**_ > **NP**_**1b/3a**_ > **NP**_**1a/3a**_. This is the same order observed for the binding of cello-oligomers by the three nanoparticles at 25 °C (Table 1, entries 4, 7, and 10). Our catalysts function by binding to a cellulose chain on the surface of cellulose crystals at the nonreducing end. Since the binding is essential to the placement of the acidic catalytic groups near the glycosidic bond(s) to be cleaved, the correlation between the binding and the catalysis is fully anticipated.

Among the three catalytic functional monomers **3a**–**3c** (Figure 3b), the ortho dicarboxylic acid-containing **3a** clearly outperforms the meta dicarboxylic acid **3b** and the amine/carboxylic acid-containing **3c** performs least effectively. Thus, at least in this case, the biomimetic motif is superior to the abiotic ones and the ortho relationship is important to the catalytic hydrolysis.

**FIGURE 4:**
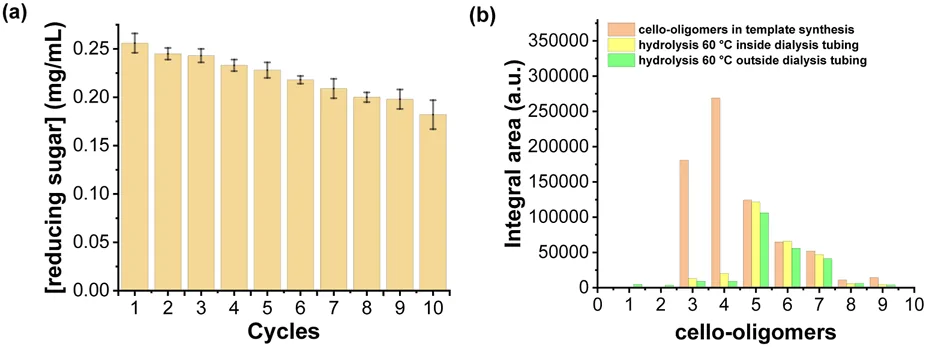
**(a)** Recyclability of **NP**_**1c/3a**_ for cellulose hydrolysis in NaOAc buffer (pH 6.5) at 60 °C. **(b)** Distribution of cello-oligomers in the original sample used in the template synthesis versus those in the products of cellulose hydrolysis by **NP**_**1c/3a**_ using 10 mM MES buffer (pH 6.5) at 60 °C.

Figure 4a shows the recyclability of **NP**_**1c/3a**,_ our most active catalyst in this study. For these experiments, 5 mg of cellulose is placed in a dialysis tubing containing the nanoparticle catalyst and the hydrolysis is allowed to occur at 60 °C for 12 h. With a MW-cutoff (MWCO) of 2000 Da, the dialysis membrane allows the sugar products formed to escape into the external solution while the catalyst and unreacted cellulose stay inside. A fresh batch of cellulose is added every 12 h, and the external solution is analyzed after each cycle. Consistent with the stability of the catalysts, **NP**_**1c/3a**_ maintains >70% of its activity after 10 cycles of usage.

The cello-oligomers used in the synthesis of template **1c** were prepared by the hydrolysis of cellulose using phosphoric acid (Liebert et al., 2008). As shown in Figure 4b, the mass spectrum is dominated by oligomers with a degree of polymerization (DP) of 3–5. When the catalytic hydrolysis by **NP**_**1c/3a**_ was performed inside the dialysis tubing, we analyzed the solutions both inside and outside. The two solutions have similar distributions of chain lengths, comprising mostly DP5–7 oligomers. Thus, the products shift to a distribution of higher molecular weight oligomers in comparison to what was used in the template synthesis. This result suggests that, when the catalysts are prepared with a mixture of template molecules as in **NP**_**1c/3a**_, not all the nanozymes have the same activity. The catalyst templated with a longer oligomer is expected to bind cellulose more strongly, as shown by our binding data (Table 1, entries 4, 7, and 10). Moreover, Figure 3a shows that the nanoparticles templated with the longer sugars have a higher catalytic activity in cellulose hydrolysis. Thus, a shift toward higher molecular weight oligomers in the product is completely expected.

## 3 Conclusion

Molecular imprinting has already been employed to endow inorganic nanozymes with enhanced substrate-specificity (Bu et al., 2024; Zhang et al., 2017b; Zhang et al., 2025). This work shows that micellar imprinting can be used to produce cellulose-hydrolyzing organic nanozymes directly in a one-pot reaction within 2 days. Design of the thiosemicarbazide-derived glycan templates is critical. Not only can these templates be made in a single step from unprotected mono- and oligosaccharides, the hydrogen-bonding properties of the thiosemicarbazide group enable the installation of the dicarboxylic acid catalytic motif found in natural glycosidases in the imprinted site via FM **3a**. Synthesis of the nanozyme in this way is greatly simplified in comparison to our previously reported procedures that require multistep synthesis of both the templates and the photoaffinity labels used in the post-modification (Zangiabadi et al., 2024; Zangiabadi and Zhao, 2022). In addition, the modular synthesis of the template allows straightforward tuning of the size of the imprinted active site, as well as the distance between the sugar-binding boroxole and the glycosidic bond-cleaving acids, so that cellulose may be hydrolyzed to afford either glucose or cello-oligomers with controlled chain lengths.

Although applied here to cellulose hydrolysis, this nanozyme methodology is fundamentally generalizable. Structurally analogous systems can be designed to process other biological glycans (Li and Zhao, 2021). The one-step synthesis of templates from free glycans and facile preparation of the catalysts are expected to be highly advantageous compared with the previouisly reported systems.

## 4 Materials and methods

### 4.1 Typical procedure for the preparation of NP catalysts

A solution of FM **2** (40 µL from 10 mM stock, 0.0004 mmol), FM **3** (40 µL from 10 mM stock, 0.0004 mmol), and template **1a–c** (40 µL from 10 mM stock, 0.0004 mmol) in methanol (5 mL) was prepared in a glass vial. After the mixture was stirred for 6 h at room temperature, methanol was removed *in vacuo* to afford the sugar-boronate complex. A micellar solution of compound **4** (13.9 mg, 0.03 mmol), compound **5** (11.1 mg, 0.02 mmol), divinylbenzene (DVB, 2.1 μL, 0.015 mmol), N,N′-methylenebis (acrylamide) (MBAm, 20 μL of a 38.5 mg/mL solution in DMSO, 0.005 mmol), and 2,2-dimethoxy-2-phenylacetophenone (DMPA, 10 μL of a 12.8 mg/mL solution in DMSO, 0.0005 mmol) in H_2_O (2.0 mL) was added to the sugar-boronate complex. The mixture was subjected to ultrasonication for 10–15 min until the mixture became clear. Then CuCl_2_ (10 μL of a 6.7 mg/mL solution in H_2_O, 0.0005 mmol) and sodium ascorbate (10 μL of a 99 mg/mL solution in H_2_O, 0.005 mmol) were added to the mixture. After the reaction mixture was stirred slowly at room temperature for 12 h, the reaction mixture was sealed with a rubber stopper, degassed three times, purged with nitrogen for 15 min, and irradiated in a Rayonet reactor for 12 h. Compound **6** (10.6 mg, 0.04 mmol), CuCl_2_ (10 μL of a 6.7 mg/mL solution in H_2_O, 0.0005 mmol), and sodium ascorbate (10 μL of a 99 mg/mL solution in H_2_O, 0.005 mmol) were added. Reaction progress was monitored by ^1^H NMR spectroscopy and nanoparticle formation was monitored by dynamic light scattering (DLS). After being stirred for another 6 h at room temperature, the reaction mixture was poured into acetone (8 mL). The precipitate collected by centrifugation was washed with a mixture of acetone/water (5 mL/1 mL) three times, followed by acetone (5 mL) two times before being dried in air to afford the **NP**_**1a-c/3a–c**_ as an off-white powder (typical yield ca. 80%).

### 4.2 LC-MS characterization of cellulose hydrolysis products

In a typical procedure, a reaction mixture containing cellulose (5.0 mg/mL) and the appropriate catalyst (cellulase or synthetic catalyst at 0.5–5.0 mg/mL) in 1.0 mL of buffer was incubated at indicated temperatures for 12 h. After cooling to room temperature, the reaction mixture was centrifuged at 20,000 RPM for 10 min to remove the nanoparticle catalyst and residual cellulose before LC-MS analysis. The reaction mixture after hydrolysis was analyzed by LC-MS analysis using an Agilent 1200 Series Binary VWD system with an Agilent 6540 UHD Accurate Mass Q-TOF mass detector. Separation of the products was performed on a Thermo Scientific HILIC-LC column (4.6 mm, 150 mm) at 60 °C. For quantitative analysis, injection volumes were adjusted for the signal intensity to stay within the linear range of the calibration curve. All samples were centrifuged at 20,000 RPM before analysis to remove the nanoparticles (to avoid column blockage over extended usage). Comparison with non-centrifuged samples showed no change in glucose concentration during the centrifugation step. The mobile phase was a mixture of acetonitrile and water, with 0.1% formic acid. Product identities were assigned by high-resolution mass spectrometry in positive-ion mode.

## Data Availability

The original contributions presented in the study are included in the article/Supplementary Material, further inquiries can be directed to the corresponding author.
