# Supplementary material for: Direct synthesis of molecularly imprinted nanozymes with a biomimetic catalytic motif for glycosidic bond cleavage
Source: Front Chem. 2026 Aug 11;14:1866827. doi: 10.3389/fchem.2026.1866827 (PMC13503606; doi:10.3389/fchem.2026.1866827)
Supplement: Supplementary file 1 [file DataSheet1.pdf]

frontiers

Supplementary Material

Direct Synthesis of Molecularly Imprinted Mimics of Cellulase Enzymes with a Double Carboxylic Acid Motif

Mohan Lakavathu and Yan Zhao*
Department of Chemistry, Iowa State University, Ames, Iowa 50011-3111, U.S.A.

Correspondence to: zhaoy@iastate.edu

Contents

1. General Methods S3
2. Synthesis S3
Scheme S1. Synthesis of Functional Monomers S3
Scheme S2. Synthesis of templates S5
3. Fluorescence Titrations S8
Table S1 .... S8
Figure S1 .... S8
4. Characterization of Nanoparticles S9
Figure S2 .... S9
Dynamic Light Scattering S9
Figure S3 .... S10
Figure S4 .... S10
Figure S5 .... S10
Transmission Electron Microscopy (TEM) S11
Figure S6 .... S11
5. ITC Binding Studies S11
Figure S7 .... S12
Figure S8 .... S12
Figure S9 .... S13
Figure S10 .... S13
Figure S11 .... S14
Figure S12 .... S14
Figure S13 .... S15
Figure S14 .... S15
Figure S15 .... S**Error! Bookmark not defined.**
Figure S16 .... S**Error! Bookmark not defined.**
6. Hydrolysis of Cellulose and Compounds 8a–f S17
Hydrolysis of cellulose S17
Figure S17 .... S17
LC-MS Characterization of Hydrolysis Products S17

Calibration for substrates S18
Figure S18 .... S18
Figure S19 .... S18
Figure S20 .... S18
Figure S21 .... S19
Table S2 .... S20
Figure S22 .... S20
7. NMR Spectra of Key Compounds S21
Figure S24 .... S21
Figure S25 .... S22
Figure S26 .... S22
Figure S27 .... S23
Figure S28 .... S23
Figure S29 .... S24
Figure S30 .... S24
Figure S31 .... S25
Figure S32 .... S25
Figure S33 .... S26
Figure S34 .... S26
Figure S35 .... S27
Figure S36 .... S27
Figure S37 .... S28
Figure S38 .... S28
Figure S39 .... S29
Figure S40 .... S29
*8. **ESI-MS Spectra of Key Compounds*** S30
References S33

1. General Methods

All organic solvents and reagents were of ACS-certified grade or higher grade and were purchased from Fisher Scientific and dried using standard procedures. Ultrapure water (18.2 MU; Millipore Co., USA) was used in all the preparation of buffers and nanoparticles. Flash column chromatography was performed on SiliFlash P60 silica gel (40-63 µm, 60 Å). All reactions were performed using standard Schlenk techniques. NMR spectra (^{1}H and ^{13}C NMR) were recorded on a Bruker DRX-400, a Bruker AV III 600, or a Varian VXR-400 spectrometer. Chemicals shifts are reported in ppm relative to residual solvent peaks ($CDCl_3$ = 7.26 ppm for ^{1}H NMR and 77.16 ppm for ^{13}C NMR, CD_3OD = 3.31 ppm for ^{1}H NMR and 49.00 for ^{13}C NMR). Coupling constants are reported in hertz. ESI-mass spectrometry was recorded on an Agilent QTOF 6540 mass spectrometer with a QTOF detector. Dynamic light scattering (DLS) was performed on a Malvern Zetasizer Nano ZS. UV-vis spectra were recorded on a Cary 100 Bio UV-visible spectrophotometer. LC-MS analysis was performed with a Thermo Scientific HPLC-LC column (4.6 mm, 150 mm) coupled to an Agilent 1200 Series Binary VWD system with an Agilent 6540 UHD Accurate Mass Q-TOF mass spectrometry detector.

2. Synthesis

Compounds **2**,[1] **5**,[2,3] **8a–8f**[4] and **12**[5] were synthesized following previously reported procedures.

Scheme S1. Synthesis of Functional Monomers

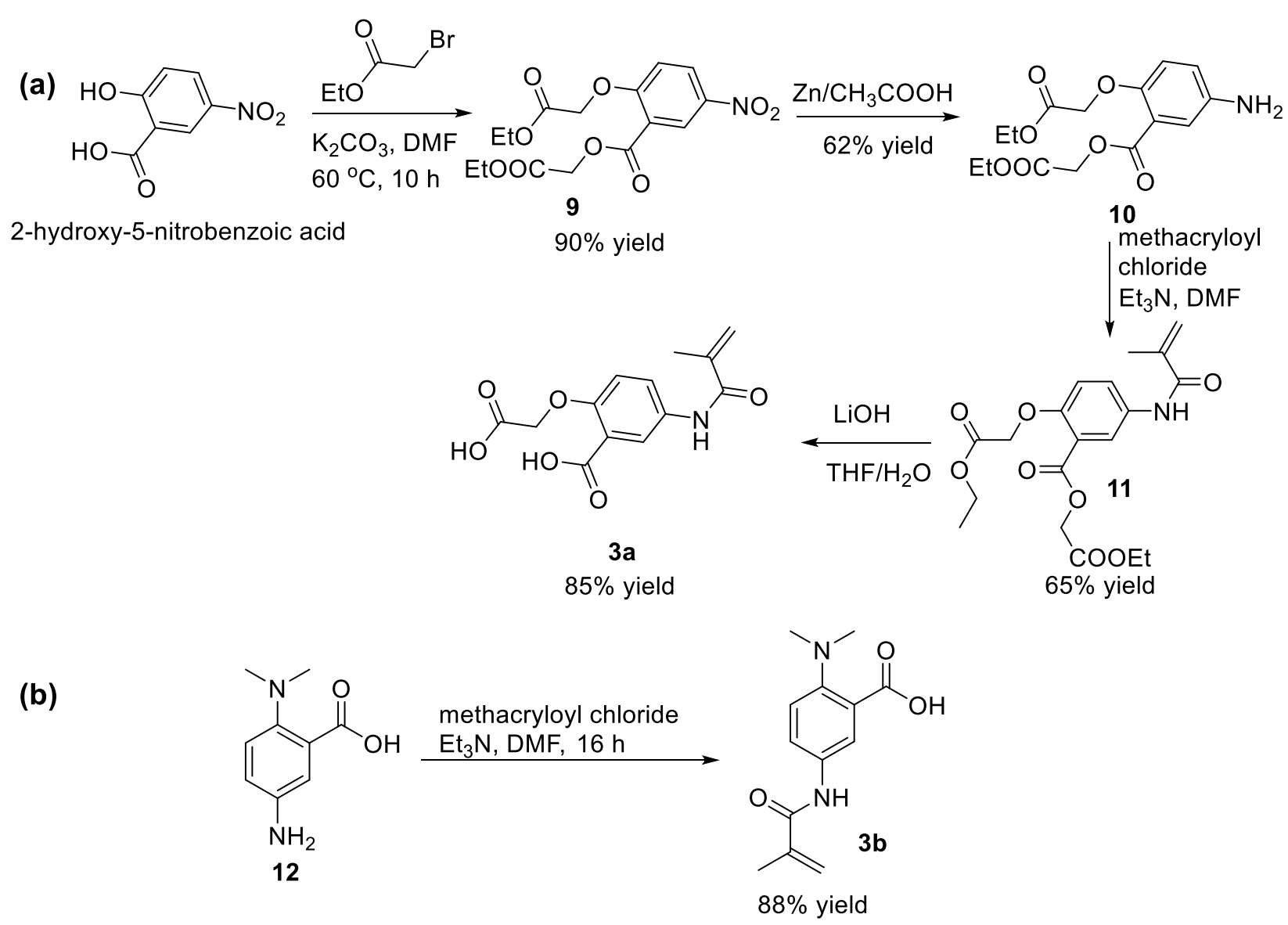

Synthesis of compound 9. Ethyl 2-bromoacetate (0.37 mL, 3.3 mmol) was added to a suspension of 2-hydroxy-5-nitrobenzoic acid (0.549 g, 3 mmol) and potassium carbonate (0.62 g, 4.5 mmol) in anhydrous DMF (5 mL). After the reaction mixture was stirred at 60 °C overnight. Then it was cooled to room temperature, diluted with water (50 mL) and extracted with ethyl acetate (3 × 30 mL). The combined organic phase was washed with brine (30 mL), dried over $MgSO_4$, and concentrated by rotary evaporation. The residue was purified by column chromatography over silica gel using 6:2 hexane/ethyl acetate as the eluent to afford **9** as a yellowish oil (0.958 g, 90%). ^{1}H NMR (400 MHz, $CDCl_3$) δ 8.07 (d, J = 8.5 Hz, 1H), 7.91 (dd, J

= 8.5, 2.1 Hz, 1H), 7.72 (d, *J* = 2.0 Hz, 1H), 4.84 (s, 2H), 4.82 (s, 2H), 4.30 – 4.23 (m, 4H), 1.32 –1.28 (m, 6H). HRMS (ESI$^+$/QTOF) Calcd for $C_{15}H_{17}NO_9$ m/z: $[M+H]^+$ 356.0937; Found m/z 356.0986.

Synthesis of compound 10. The compound **9** (0.355 g, 1 mmol) was dissolved in 2:1 ethanol/ethyl acetate (5.0 mL), followed by the addition of zinc powder (0.130 g, 2 mmol) and acetic acid (2.0 mL). The reaction mixture was stirred at 50 °C for 2 h. The mixture was cooled to room temperature. Ethyl acetate (30 mL) was added, followed by saturated Na_2CO_3 (30 mL). The aqueous layer was extracted with ethyl acetate (3 × 30 mL). The combined organic phase was washed with water (30 mL) and brine (30 mL). The organic layer was dried over $MgSO_4$ and concentrated via rotary evaporation to afford a yellow oil (0.220 g, 62%). ^{1}H NMR (400 MHz, DMSO-d6) δ 7.57 (d, *J* = 8.6 Hz, 1H), 6.18 (dd, *J* = 8.6, 2.0 Hz, 1H), 6.03 (s, 1H), 6.03 – 6.02 (m, 1H), 4.68 –4.67 (m, 4H), 4.18 – 4.07 (m, 4H), 1.22–1.14 (m, 6H). HRMS (ESI$^+$/QTOF) Calcd for $C_{15}H_{19}NO_7$ m/z: $[M+H]^+$ 326.1196; Found m/z 326.1195.

Synthesis of compound 11. Methacryloyl chloride (0.20 mL, 2.1 mmol) was added dropwise to a solution of compound **10** (0.478g, 2 mmol) and triethylamine (0.35 mL, 2.5 mmol) in DMF (3 mL) at 0 ºC. After 16 h, an aqueous solution of HCl (1 N, 30 mL) was added. The mixture was extracted with ethyl acetate (3 × 10 mL). The combined organic phase was washed with water (1 × 10 mL) and brine (1 × 10 mL), dried over $MgSO_4$, and concentrated by rotary evaporation. The residue was purified by column chromatography over silica gel using 10:1 hexane/ethyl acetate as the eluent to give compound **10** as an off-white powder (0.362 g, 65 %). ^{1}H NMR (400 MHz, $CDCl_3$) δ 7.93 (d, *J* = 8.5 Hz, 1H), 7.70 (m, 1H), 7.66 (d, *J* = 8.3 Hz, 1H), 6.94 (dd, *J* = 8.6, 2.0 Hz, 1H), 5.79 – 5.73 (m, 1H), 5.51 (q, *J* = 1.5 Hz, 1H), 4.80 (s, 2H), 4.74 (s, 2H), 4.27 – 4.23 (m, 4H), 2.05 (s, 3H), 1.30–1.27 (m, 6H). HRMS (ESI$^+$/QTOF) Calcd for $C_{19}H_{23}NO_8$ m/z: $[M+H]^+$ 394.1457; Found m/z 394.1465.

Synthesis of compound 3a. The LiOH (0.07 g, 3 mmol) was added to a solution of compound **11** (0.61 g, 2 mmol) in 1:1 mixture of THF and water (10 mL). After stirred at room temperature for 6 h, the reaction mixture was diluted with 1 M HCl aqueous solution and extracted with ethyl acetate (3 × 20 mL). The combined organic phase was washed with brine (20 mL), dried over $MgSO_4$, and concentrated by rotary evaporation. The residue was purified by column chromatography over silica gel using 5:2 hexane/ethyl acetate as the eluent to afford **3a** as a yellow solid (0.476 g, 85 %). ^{1}H NMR (400 MHz, DMSO-d6) δ 10.03 (s, 1H), 7.71 (d, *J* = 8.6 Hz, 1H), 7.48 – 7.41 (m, 2H), 5.82 (s, 1H), 5.57 (s, 1H), 4.71 (s, 2H), 1.95 (s, 3H). ^{13}C NMR (101 MHz, DMSO-d6) δ 170.26, 167.68, 166.76, 158.20, 144.24, 140.60, 132.63, 121.08, 115.64, 112.26, 104.84, 65.62, 19.10. HRMS (ESI$^+$/QTOF) Calcd for $C_{13}H_{13}NO_6$ m/z: $[M+H]^+$ 280.0776; Found m/z 280.0770.

Synthesis of compound 3b. Methacryloyl chloride (0.20 mL, 2.1 mmol) was added dropwise to a solution of 5-amino-2-(dimethyl amino) benzoic acid[5] (0.360 g, 2 mmol) and triethylamine (0.35 mL, 2.5 mmol) in DMF (3 mL) at 0 ºC. After 16 h, an aqueous solution of HCl (1 N, 30 mL) was added. The mixture was extracted with ethyl acetate (3 × 10 mL). The combined organic phase was washed with water (1 × 10 mL) and brine (1 × 10 mL), dried over $MgSO_4$, and concentrated by rotary evaporation. The residue was purified by column chromatography over silica gel using

10:1 hexane/ethyl acetate as the eluent to give **3b** as light-yellow powder (0.436 g, 88 %). NMR (600 MHz, $CDCl_3$) δ 8.05 (s, 1H), 6.25 – 6.21 (m, 2H), 5.87 – 5.86 (m, 1H), 5.83– 5.82 (m, 1H), 5.65– 5.63 (m, 1H), 2.98 (s, 3H), 2.90 (s, 3H), 2.00 (s, 3H). HRMS (ESI^+/QTOF) Calcd for $C_{13}H_{16}N_2O_3$ m/z: $[M+H]^+$ 249.1194; Found m/z 249.1190.

Scheme S2. Synthesis of templates

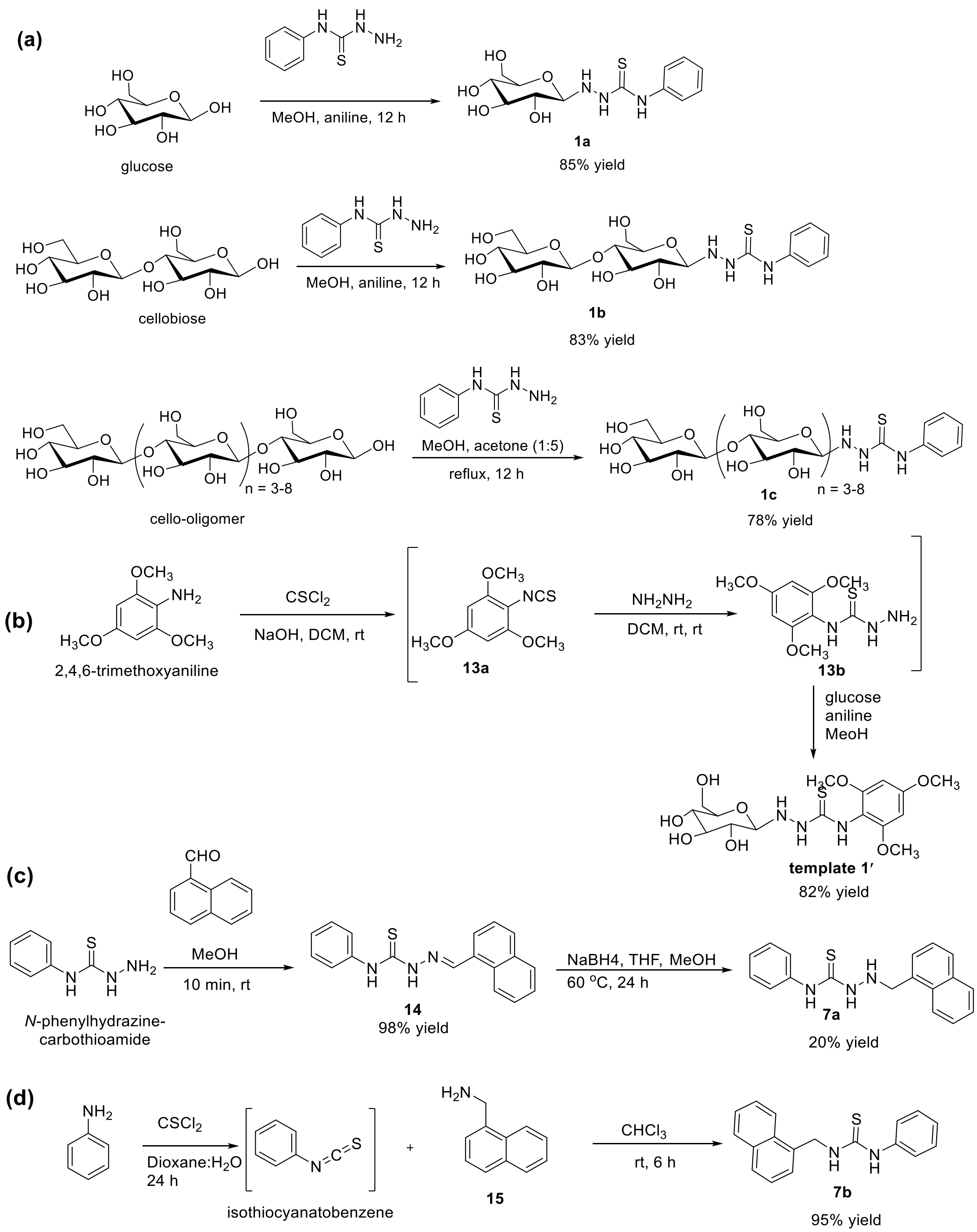

Synthesis of compound 1a.[6] Aniline (2.3 μL, 0.03 mmol) was added to a solution of glucose (0.180 g, 1 mmol) and 4-phenylthiosemicarbazide (0.167 g, 1 mmol) in methanol (3 mL). The mixture was heated to reflux overnight, and the solvent was removed by rotary evaporation after the mixture was cooled to room temperature. The residue was purified by column chromatography over silica gel using 1:1 dichloromethane/methanol as the eluent to afford **1a** as a pale-yellow powder (0.279 g, 85%). ^{1}H NMR (600 MHz, DMSO-d6) δ 9.75 (s, 1H), 9.13 (s, 1H), 7.67 (s, 1H), 7.31 (t, J = 7.7 Hz, 2H), 7.11 (t, J = 7.4 Hz, 1H), 6.21 (d, J = 4.6 Hz, 1H), 4.92 (t, J = 4.1 Hz, 1H), 4.77 (d, J = 5.4 Hz, 1H), 4.63 (d, J = 4.7 Hz, 1H), 4.46 (d, J = 6.7 Hz, 1H), 4.36 (t, J = 5.9 Hz, 1H), 3.62– 3.55 (m, 2H), 3.47 – 3.41 (m, 2H), 3.14– 3.10 (m, 1H), 3.07– 3.03 (m, 1H). ^{13}C NMR (101 MHz, DMSO-d6) δ 180.01, 139.59, 129.45, 129.27, 128.54, 125.69, 125.02, 124.61, 114.34, 90.95, 78.04, 77.42, 71.60, 70.95, 62.55. HRMS (ESI$^+$/QTOF) Calcd for $C_{13}H_{19}N_3O_5S$ m/z: $[M+H]^+$ 330.1079; Found m/z 330.1070.

Synthesis of compound 1b. Aniline (2.3 μL, 0.03 mmol) was added to a solution of cellobiose (0.342 g, 1 mmol) and 4-phenylthiosemicarbazide (0.167 g, 1 mmol) in methanol (3 mL). The mixture was heated to reflux overnight, and the solvent was removed by rotary evaporation after the mixture was cooled to room temperature. The residue was purified by column chromatography over silica gel using 1:1 dichloromethane/methanol as the eluent to afford **1b** as a yellow powder (0.407 g, 83%). ^{1}H NMR (600 MHz, DMSO-d6) δ 9.88 (s, 1H), 9.12 (s, 1H), 7.65 (s, 2H), 7.31 (t, J = 7.7 Hz, 2H), 7.11 (t, J = 7.4 Hz, 1H), 6.66 (d, J = 3.5 Hz, 1H), 5.24 (t, J = 4.6 Hz, 1H), 5.03 – 4.97 (m, 2H), 4.91 (t, J = 4.2 Hz, 1H), 4.77 (s, 1H), 4.67 – 4.58 (m, 2H), 4.51 – 4.49 (m, 1H), 4.35 – 4.32 (m, 1H), 4.27– 4.97 (m, 1H), 3.72 – 3.70 (m, 2H), 3.66 – 3.54 (m, 2H), 3.44 – 3.40 (m, 1H), 3.31 – 3.29 (m, 2H), 3.22 – 3.15 (m, 2H), 3.10 – 2.95 (m, 2H). ^{13}C NMR (101 MHz, DMSO) δ 179.98, 139.56, 129.44, 129.20, 128.53, 125.02, 124.60, 90.93, 78.03, 77.40, 73.53, 71.59, 70.94, 62.53. HRMS (ESI$^+$/QTOF) Calcd for $C_{19}H_{29}N_3O_{10}S$ m/z: $[M+H]^+$ 492.1607; Found m/z 492.1628.

Synthesis of template 1'. The NaOH (0.120 g, 3 mmol) was added to a solution of the 2,4,6-trimethoxyaniline (0.183 g, 1 mmol) in DCM, and the resulting mixture was cooled to 0 °C. Thiophosgene (0.228 mL, 3 mmol) was added dropwise, and the reaction mixture was stirred for 2 h at 30 °C. The reaction was quenched with water and extracted with DCM (2 × 10 mL). The combined organic layers were washed with brine, dried over Na_2SO_4, and concentrated under reduced pressure to afford the isothiocyanate intermediate. This intermediate was dissolved in DCM, cooled to 0 °C, and hydrazine (0.038 g, 1.2 mmol) was added through the solution for 10 min. The resulting solid was filtered and dried in vacuo to obtain the desired product **13b**. The Aniline (2.3 μL, 0.03 mmol) was added to a solution of glucose (0.180 g, 1 mmol) and compound **13b** (0.257 g, 1 mmol) in methanol and acetone mixture (1:1, 3 mL). The mixture was heated to reflux overnight, and the solvent was removed by rotary evaporation after the mixture was cooled to room temperature. The residue was purified by column chromatography over silica gel using 1:1 dichloromethane/methanol as the eluent to afford template **1'** as a pale brown powder (0.343 g, 82%). ^{1}H NMR (600 MHz, DMSO-d6) δ 10.21 (s, 1H), 6.38 (s, 1H), 6.27 (s, 2H), 6.18 (s, 1H), 4.79 (s, 1H), 4.69 – 4.67 (m, 3H), 4.66 – 4.63 (m, 1H), 4-57 – 4.54 (m, 2H), 3.88– 3.79 (m, 9H), 3.65 – 3.63 (m, 2H), 3.24 – 3.21 (m, 2H), 3.18 (s, 1H). ^{13}C NMR (151 MHz, DMSO-d6) δ 184.26, 159.30, 156.45, 91.21, 87.13, 81.26, 76.42, 74.48, 72.94, 68.95, 61.06, 55.79, 55.63. HRMS (ESI$^+$/QTOF) Calcd for $C_{16}H_{25}N_3O_8S$ m/z: $[M+H]^+$ 420.1362; Found m/z 420.1396.

Synthesis of compound 14. 4-Phenylthiosemicarbazide (0.167 g, 1 mmol) was added to a solution of naphthaldehyde (0.156 g, 1 mmol) in methanol (3 mL). The mixture was stirred at room temperature for 10 min. The resulting precipitation was filtered and dried to afford the product as a pale white powder (0.298 g, 98%). ^{1}H NMR (400 MHz, DMSO-d6) δ 11.86 (s, 1H), 10.17 (s, 1H), 9.08 (s, 1H), 8.45 (d, *J* = 7.3, 1H), 8.33 (d, *J* = 8.6 Hz, 1H), 8.03 (t, *J* = 6.2 Hz, 2H), 7.68 – 7.58 (m, 5H), 7.39 (t, *J* = 7.9 Hz, 2H), 7.24 – 7.20 (m, 1H). ^{13}C NMR (101 MHz, DMSO-d6) δ 176.41, 141.49, 139.58, 133.86, 131.17, 130.87, 129.61, 129.38, 128.57, 127.70, 126.63, 126.22, 126.05, 126.02, 125.80, 122.96. HRMS (ESI$^+$/QTOF) Calcd for $C_{18}H_{15}N_3S$ m/z: $[M+H]^+$ 306.1059; Found m/z 306.1107.

Synthesis of compound 7a. Compound **14** (0.305 g, 1 mmol) was added to a solution of $NaBH_4$ (0.148 g, 4 mmol) in mixture of methanol and THF (1:1, 5 mL). The mixture was stirred at 60 ºC for 24 h. The reaction mixture was quenched with dilute 2N HCl and extracted with ethyl acetate or EtOAc (3 × 10 mL). The combined organic phase was dried ($MgSO_4$), concentrated in vacuo, and purified via silica gel column chromatography (hexane: ethyl acetate, 3:1) to afford compound **7a** (0.061 g, 20%) as a white powder. ^{1}H NMR (400 MHz, DMSO-d6) δ 9.82 (s, 1H), 9.25 (s, 1H), 8.36 (s, 1H), 8.23 – 8.20 (m, 1H), 8.10 – 8.06 (m, 1H), 8.01 – 7.97 (m, 1H), 7.78 – 7.73 (m, 3H), 7.64 – 7.58 (m, 3H), 7.45 – 7.37 (m, 3H), 7.24 – 7.18 (m, 1H), 5.32 – 5.29 (m, 2H). ^{13}C NMR (101 MHz, DMSO-d6) δ 179.84, 139.99, 133.70, 131.37, 128.88, 128.52, 127.16, 126.32, 126.01, 124.63, 124.00, 43.48. HRMS (ESI$^+$/QTOF) Calcd for $C_{18}H_{17}N_3S$ m/z: $[M-H]^-$ 306.1070; Found m/z 306.1059.

Synthesis of compound 7b. The compound isothiocyanatobenzene (0.135 g, 1 mmol) was added to a solution of naphthalen-1-ylmethanamine (0.157 g, 1 mmol) in chloroform (3 mL). The mixture was stirred at room temperature for 6 h. The reaction mixture was extracted with chloroform (3 × 10 mL). The combined organic phase was dried ($MgSO_4$), concentrated in vacuo to afford compound **7b** (0.277 g, 95%) as a white powder. ^{1}H NMR (400 MHz, DMSO-d6) δ 9.72 (s, 1H), 8.26 (s, 1H), 8.16 – 8.10 (m, 1H), 8.01–7.97 (m, 1H), 7.88 (dd, *J* = 7.1, 2.4 Hz, 1H), 7.65 – 7.62 (m, 2H), 7.54 – 7.49 (m, 4H), 7.33 – 7.29 (m, 2H), 7.12 – 7.09 (m, 1H), 5.20 (d, *J* = 5.4 Hz, 2H). ^{13}C NMR (101 MHz, DMSO-d6) δ 181.12, 139.89, 134.54, 133.80, 133.71, 131.41, 129.55, 129.15, 129.05, 129.00, 128.16, 127.68, 127.23, 126.84, 126.74, 126.36, 125.94, 125.86, 124.57, 123.99, 123.93, 123.39, 45.86. HRMS (ESI$^+$/QTOF) Calcd for $C_{18}H_{16}N_2S$ m/z: $[M-H]^-$ 293.1107; Found m/z 293.1109.

3. Fluorescence Titrations

Table S1. Fluorescence-based binding analysis of model templates **7a** and **7b** with **3a** and 4-vinyl benzoic acid.[a]

entry	template	FM	k (x10^6)
1	**7a**	**3a**	7.1 ± 0.7
2	**7a**	**VB**	1.2 ± 0.2
3	**7b**	**3a**	1.1 ± 0.2

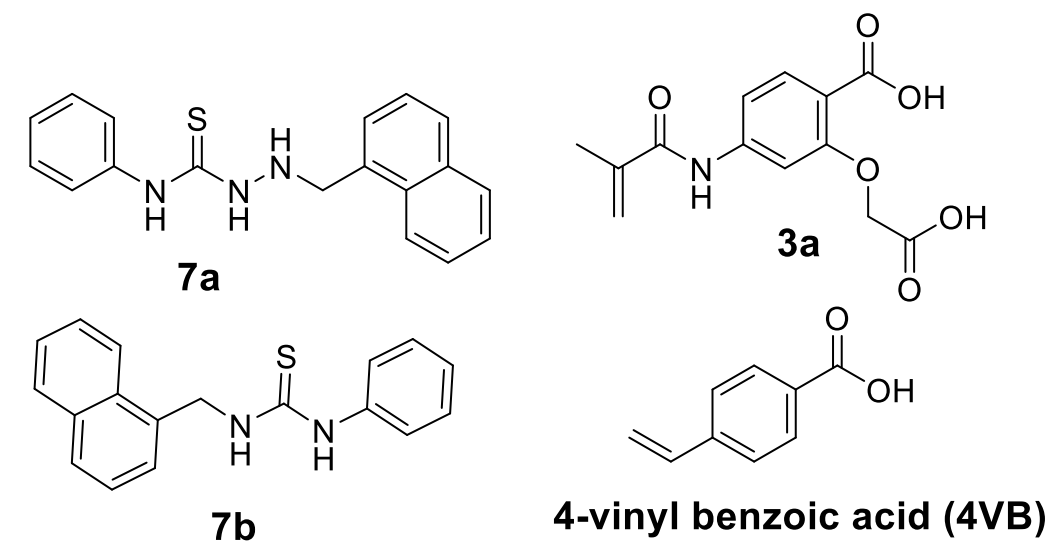

[a]Fluorescence-based binding analysis of template and acid (b) **7a** with **3a**; (d) **7b** with 4-vinyl benzoic acid and (f) **7b** with **3a** in 2 mM CTAB (Cetyltrimethylammonium bromide) solution at 25 °C.

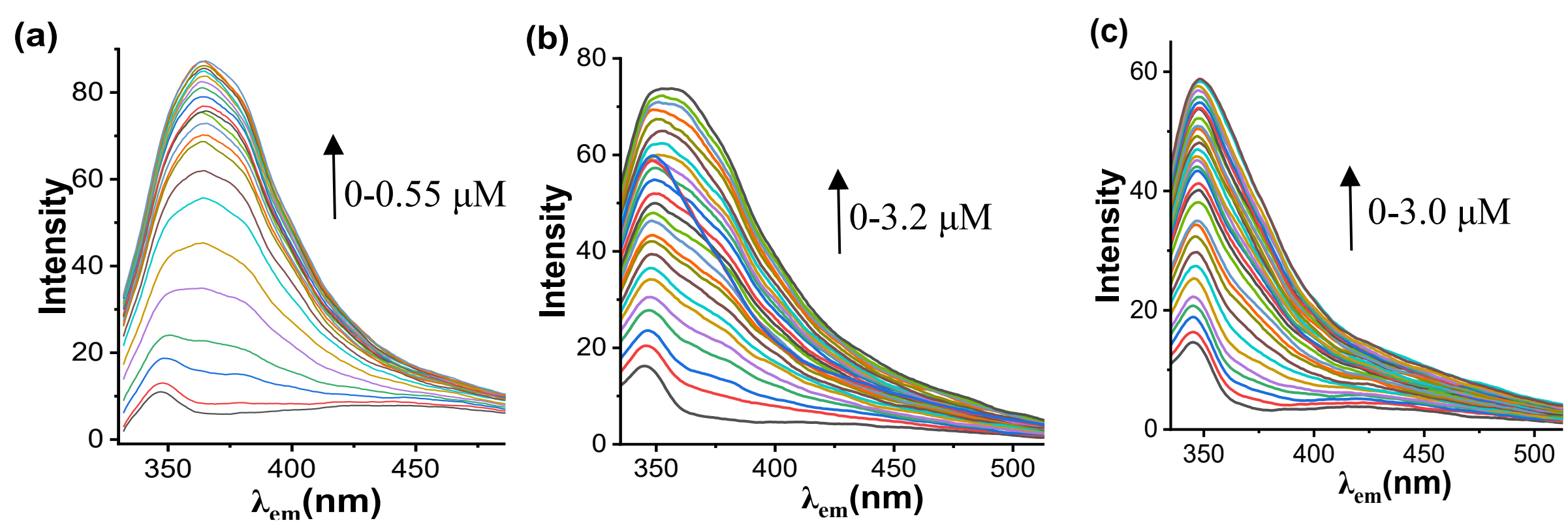

Figure S1. (a) Fluorescence spectra upon titration of **7a** by **3a** in a CTAB solution at room temperature. [**7a**] = 1.0 nM; [**3a**] = 0–0.55 μM from bottom to top; [CTAB] = 2.0 mM. (b) Fluorescence spectra upon titration of **7b** by **3a** in a CTAB solution at room temperature. [**7b**] = 1.0 nM; [**3a**] = 0–3.2 μM from bottom to top; [CTAB] = 2.0 mM. (c) Fluorescence spectra upon titration of **7a** by 4VB in a CTAB solution at room temperature. [**7a**] = 2.0 nM; [4VB] = 0–3.0 μM from bottom to top; [CTAB] = 2.0 mM. The excitation wavelength (λ_{ex}) was 311 nm. The excitation slit width was 10 nm, and the emission slit width was 5 nm. The binding constant was obtained by nonlinear least squares fitting in OriginPro of the emission intensity to a 1:1 binding isotherm.

4. *Characterization of Nanoparticles*

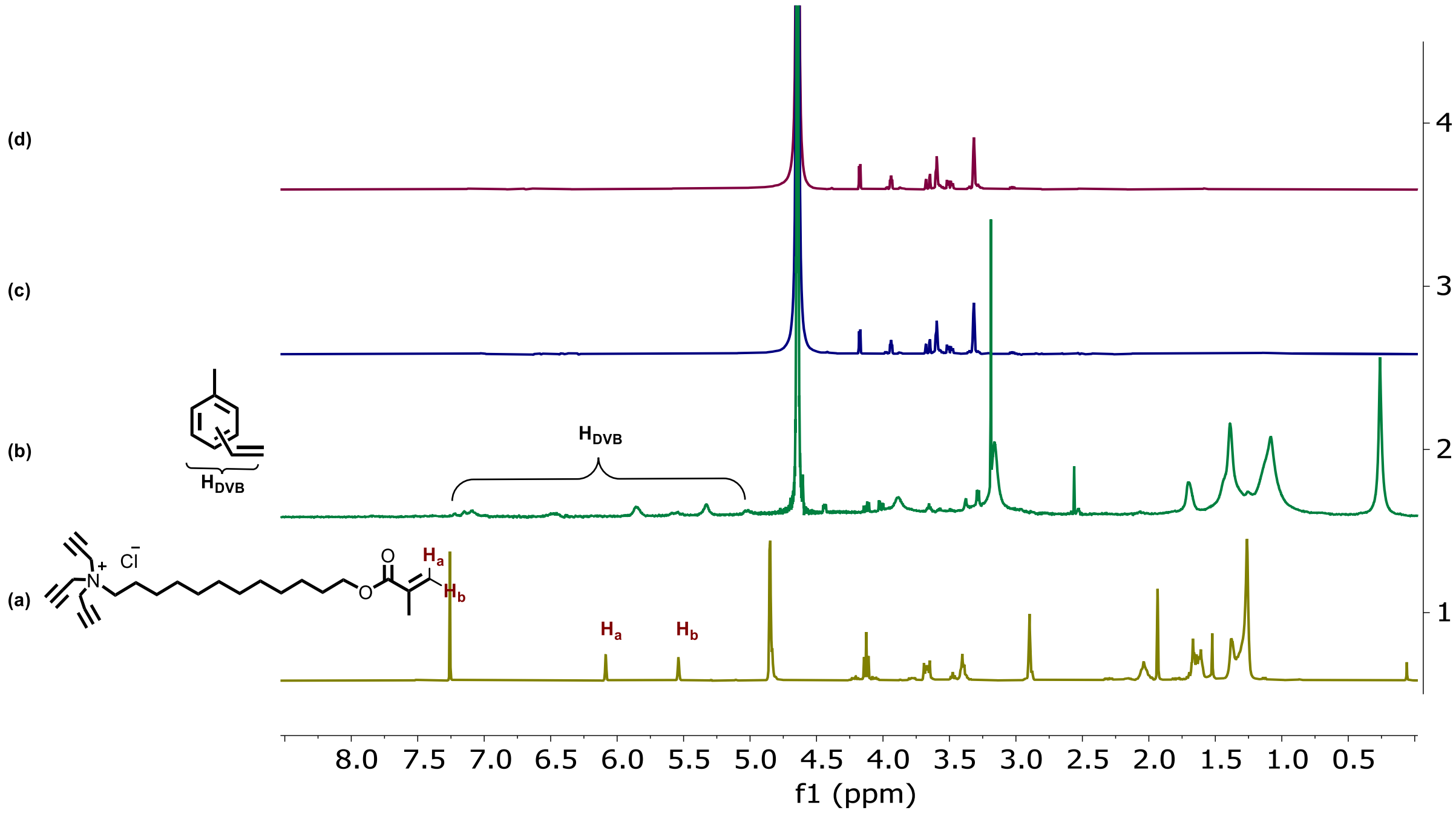

Figure S2. Stacked ^{1}H NMR spectra of (a) surfactant 1 in $CDCl_3$, (b) typical alkynyl-SCM in D_2O, (c) typical surface-functionalized SCM in D_2O, and (d) **NP1a/3a-c** after washing and redissolving in D_2O.

Dynamic Light Scattering

The particle size of MINP was determined on a Malvern Zetasizer Nano ZS using the Zetasizer software according to the Stokes-Einstein equation (1). The volume of a spherical nanoparticle (V_{D_h}) was calculated from equation (2). Assuming a density of 1.37 g/cm^3 (the density of protein), the molecular weight of the particle can be calculated using equation (3).[7] A nanoparticle with a hydrodynamic diameter of 4.87 nm has a calculated molecular weight of 50 kDa, which was used in making MINP solution for ITC titration.

$$D_h = \frac{k_B T}{6\pi\eta D_t} \qquad (1)$$

in which D_h is the hydrodynamic diameter, D_t the translational diffusion coefficient measured by dynamic light scattering, T the temperature, k_B the Boltzmann's constant, and η is dynamic viscosity of water (0.890 cP at 298 K).

$$V_{D_h} = \frac{4\pi}{3}\left(\frac{D_h}{2}\right)^3 \qquad (2)$$

$$\text{Mw in dalton} = \left(\frac{D_h}{0.132}\right)^3 \qquad (3)$$

in which D_h is the hydrodynamic diameter in nm.

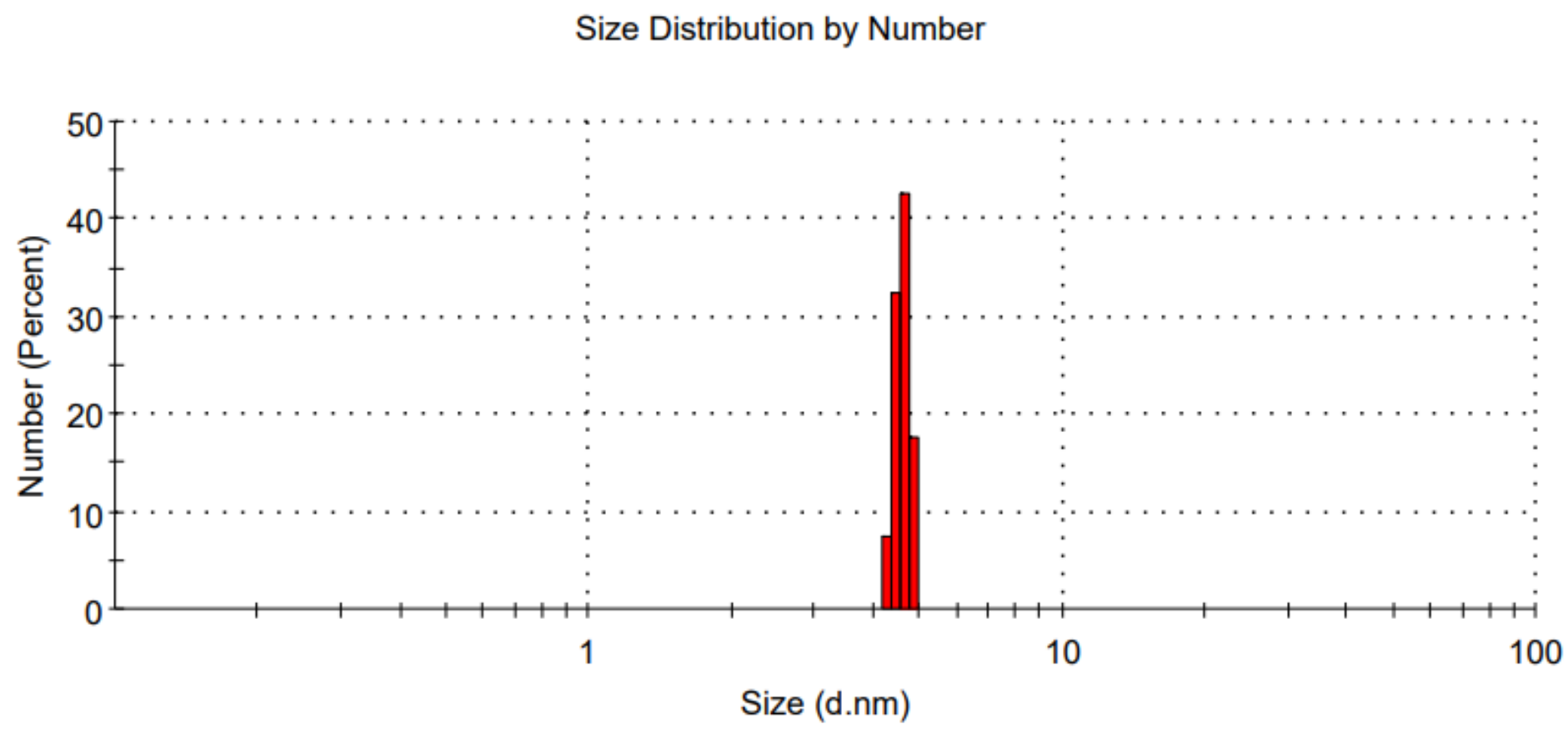

Figure S3. Distribution of the hydrodynamic diameters of the nanoparticles in water as determined by DLS for alkynyl-SCM (surface-cross-linked micelle). D = 4.67 ± 0.22 nm.

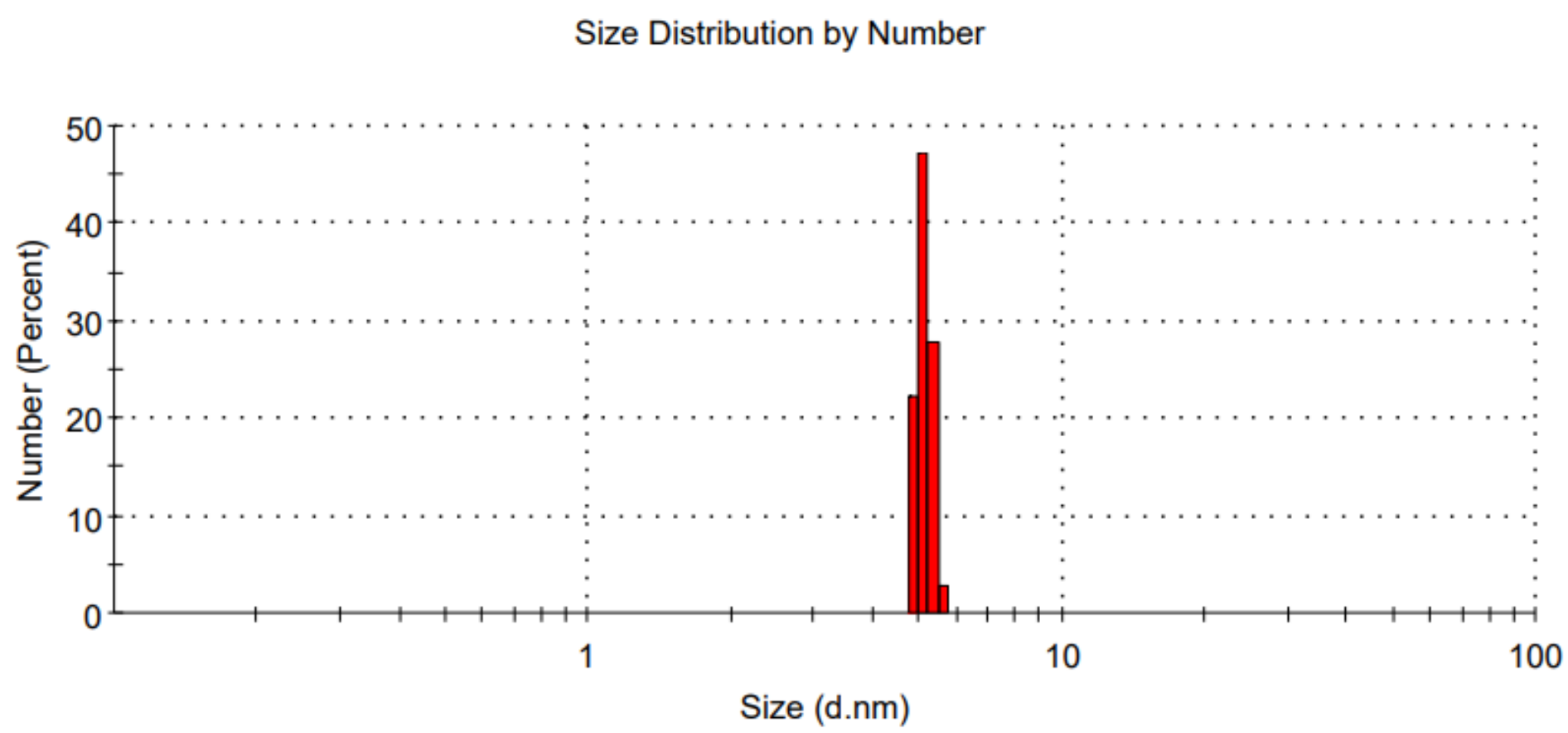

Figure S4. Distribution of the hydrodynamic diameters of the nanoparticles in water as determined by DLS for surface functionalized SCM (surface-cross-linked micelle). D = 5.32 ± 0.24 nm.

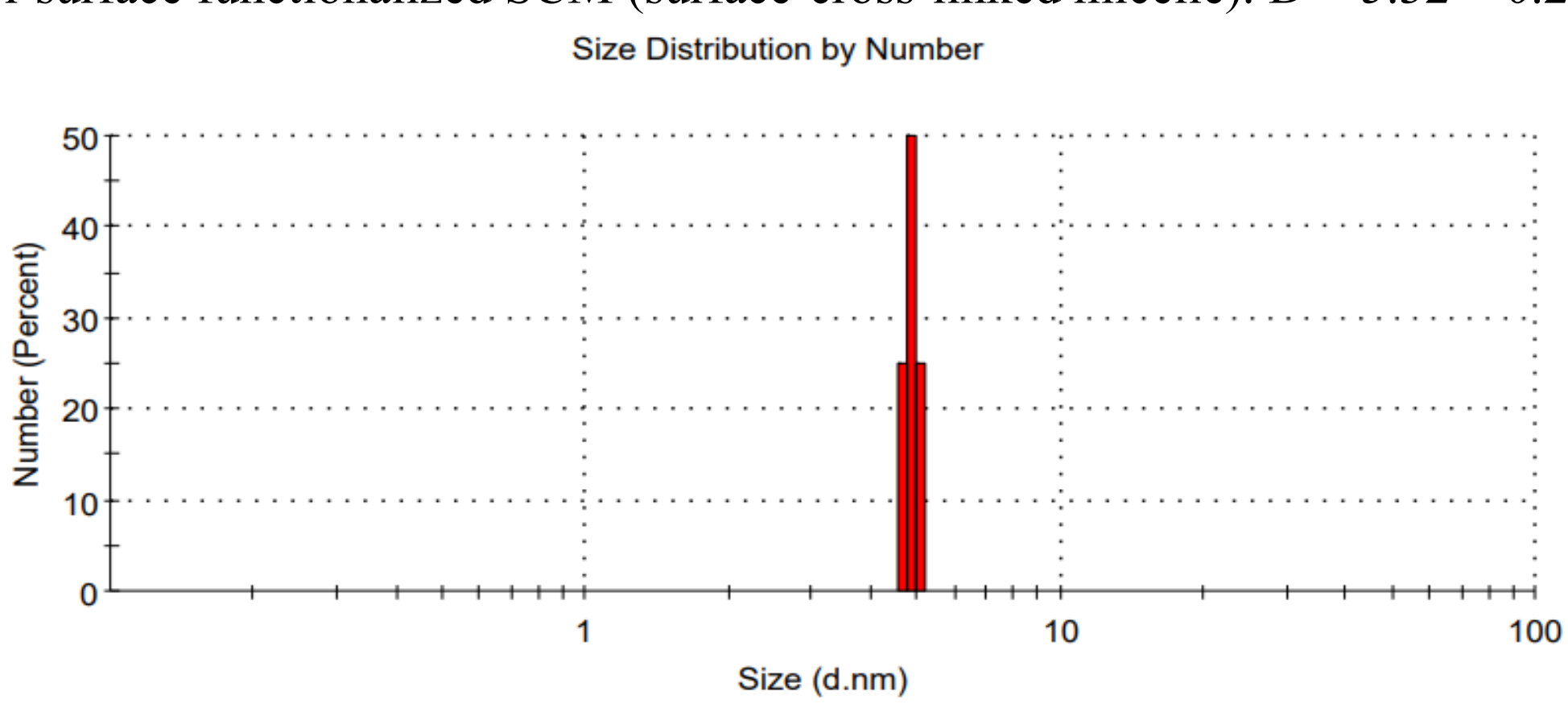

Figure S5. Distribution of the hydrodynamic diameters of the nanoparticles in water as determined by DLS for NP3a D = 4.81 ± 0.18 nm.

Transmission Electron Microscopy (TEM)

For the TEM imaging, 0.1 mg of nano catalyst was dissolved in 1 mL of Millipore water and the solution was ultra-sonicated for 10 min. A micro syringe was used to load one small drop (~1 μL) of the above solution onto a TEM copper grid covered with carbon film. The sample was left to form a thin layer, and then one small drop (~1 μL) of 2% uranyl acetate solution was loaded on the grid for the negative staining. The sample was left to dry and analyzed on a 200kV JEOL 2100 scanning/transmission electron microscope (STEM).

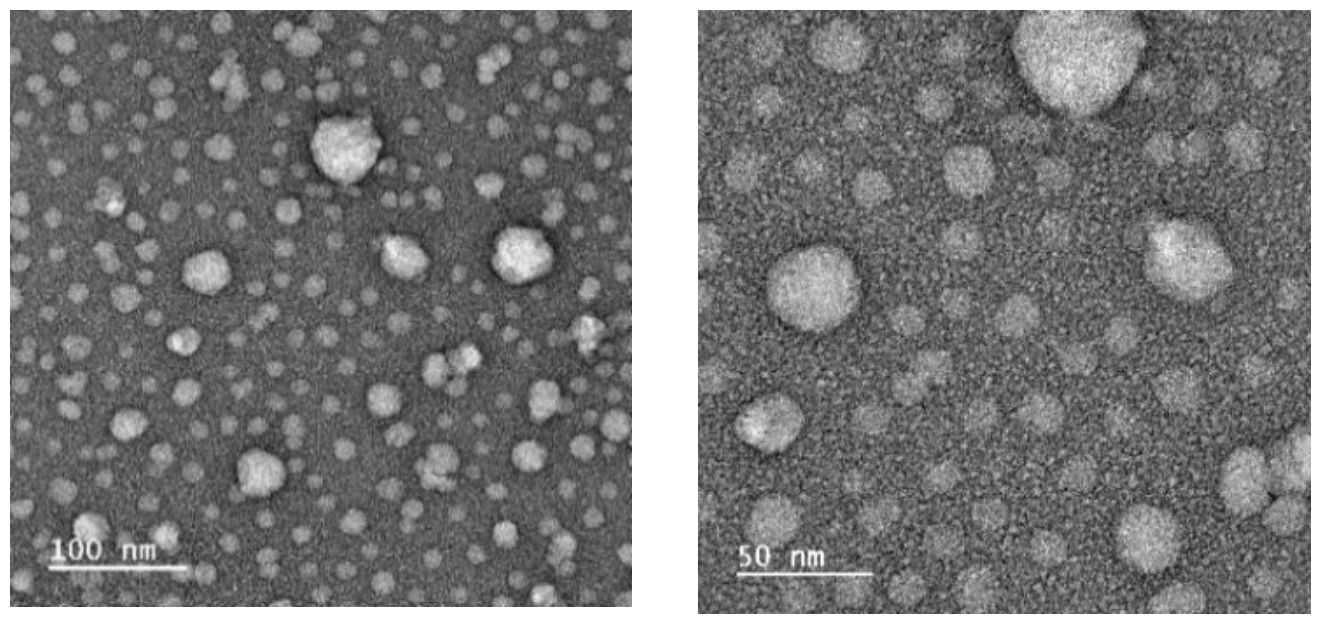

Figure S6. TEM image of typical NPs.

5. ITC Binding Studies

The determination of binding constants by ITC followed standard procedures at 298.15 K [8-10]. In general, a solution of an appropriate sugar in 10 mM MES buffer (pH 6.5) was injected in equal steps into 1.43 mL of the corresponding NP in the same solution. The top panel shows the raw calorimetric data. The area under each peak represents the amount of heat generated at each injection and is plotted against the molar ratio of the NP to the guest. The smooth solid line is the best fit of the experimental data to the sequential binding of N binding site on the NP. The heat of dilution for the guest, obtained by titration carried out beyond the saturation point, was subtracted from the heat released during the binding. Binding parameters were auto generated after curve fitting using Microcal Origin 7.

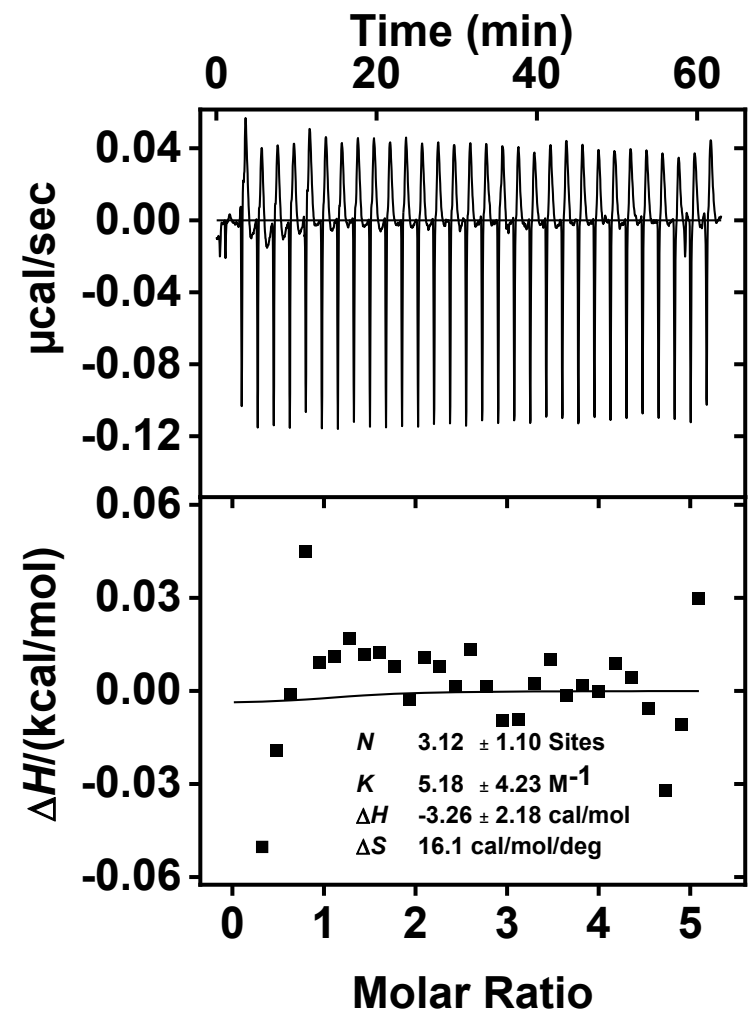

Figure S7. ITC titration curve obtained at 298 K for NINP titrated with glucose in 10 mM MES buffer at pH 6.5. The data corresponds to entry 1 in Table 1.

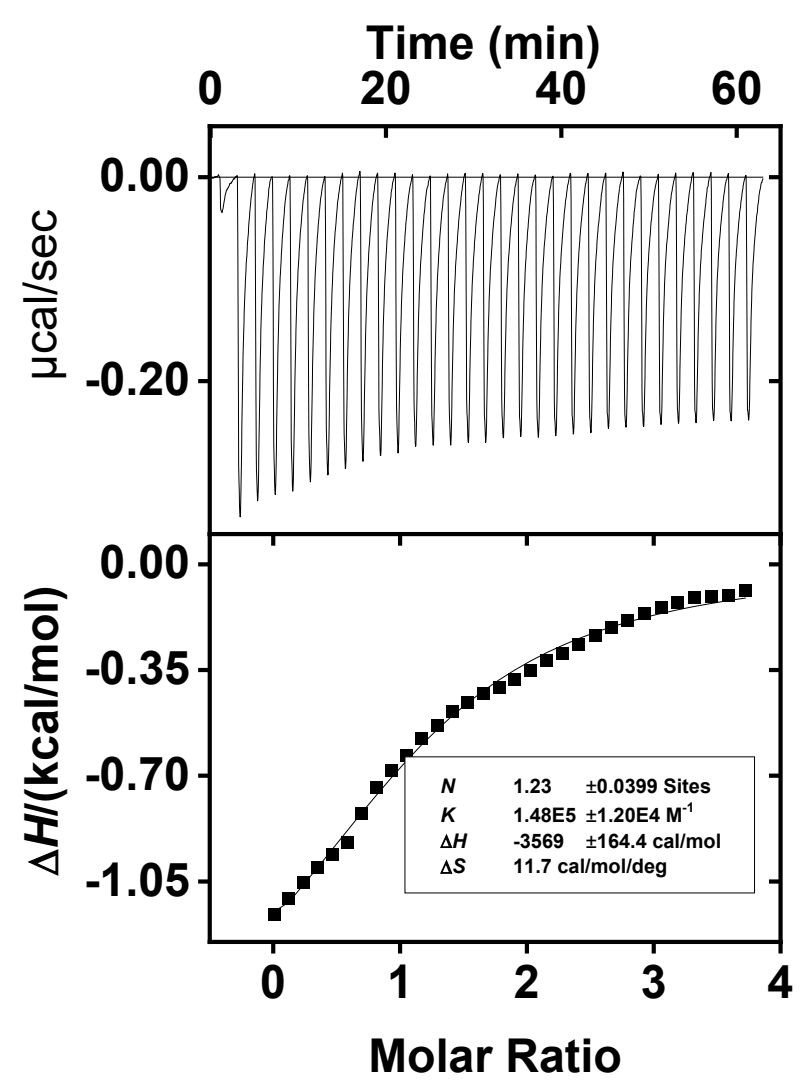

Figure S8. ITC titration curve obtained at 298 K for **NP1a/3a** titrated with glucose in 10 mM MES buffer at pH 6.5. The data corresponds to entry 2 in Table 1.

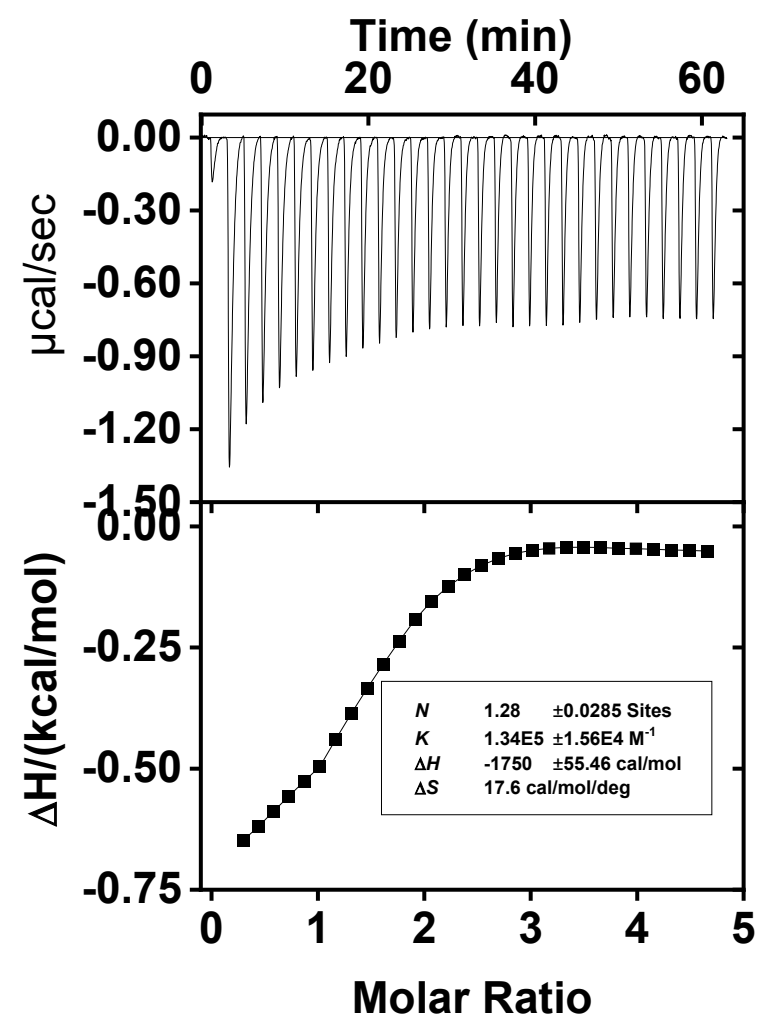

Figure S9. ITC titration curve obtained at 298 K for **NP1a/3a** titrated with cellobiose in 10 mM MES buffer at pH 6.5. The data corresponds to entry 3 in Table 1.

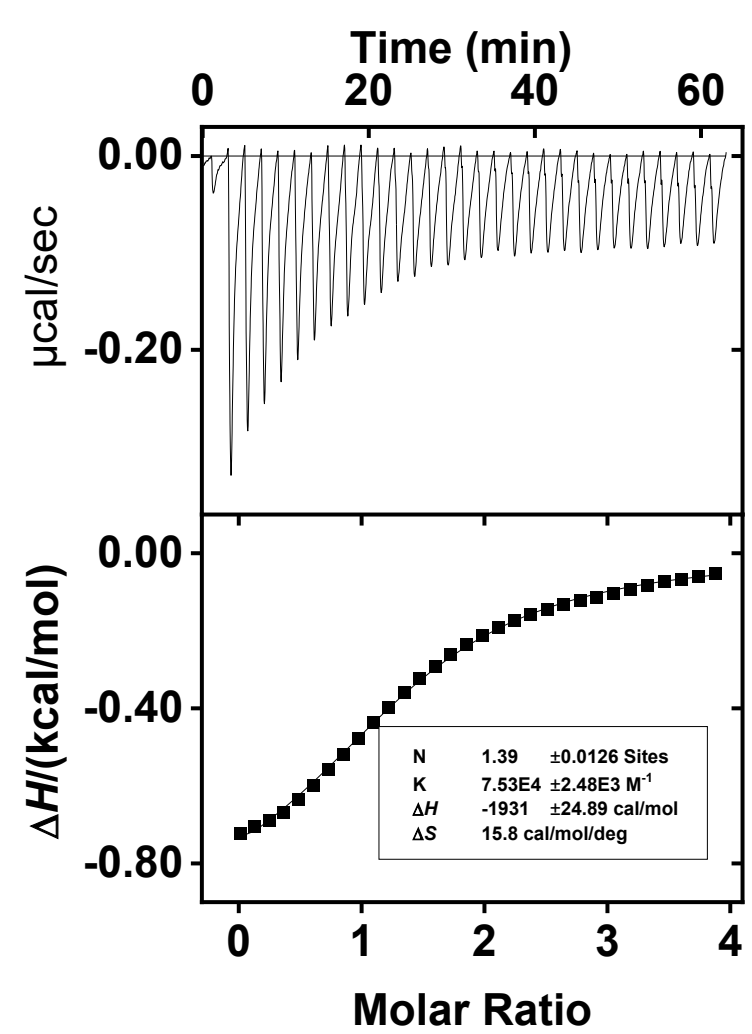

Figure S10. ITC titration curve obtained at 298 K for **NP1a/3a** titrated with cello-oligomer in 10 mM MES buffer at pH 6.5. The data corresponds to entry 4 in Table 1.

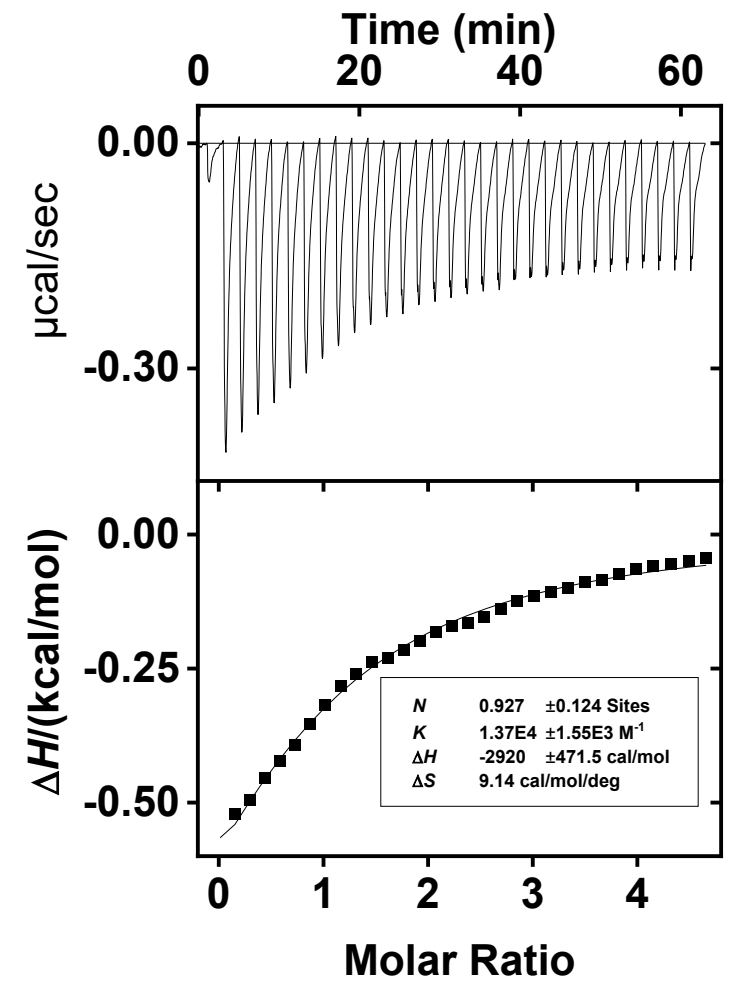

Figure S11. ITC titration curve obtained at 298 K for **NP1b/3a** titrated with glucose in 10 mM MES buffer at pH 6.5. The data corresponds to entry 5 in Table 1.

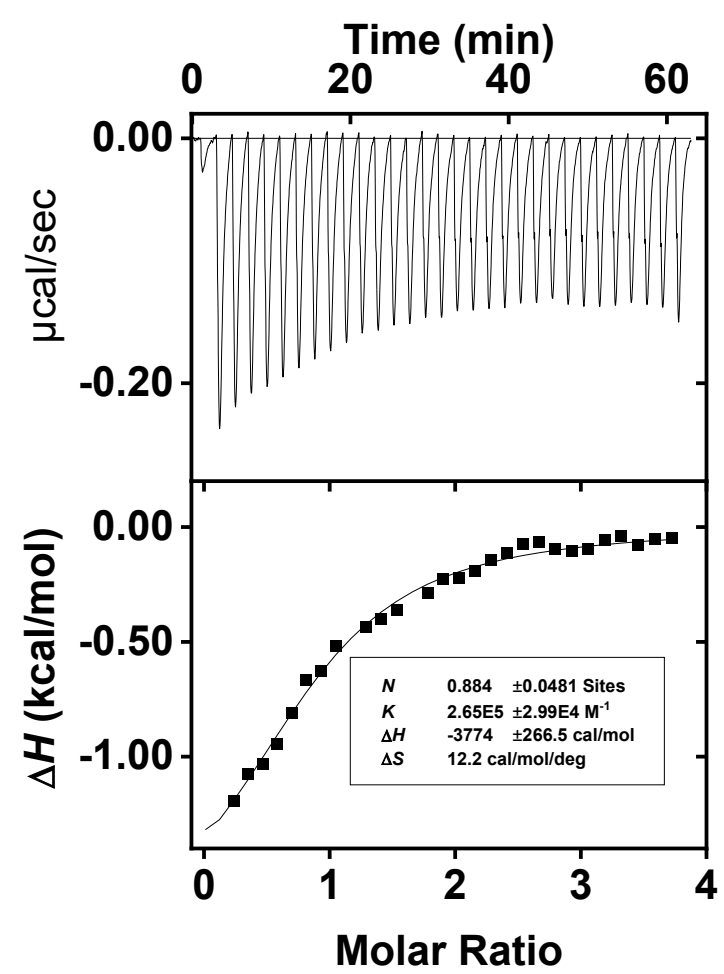

Figure S12. ITC titration curve obtained at 298 K for **NP1b/3a** titrated with cellobiose in 10 mM MES buffer at pH 6.5. The data corresponds to entry 6 in Table 1.

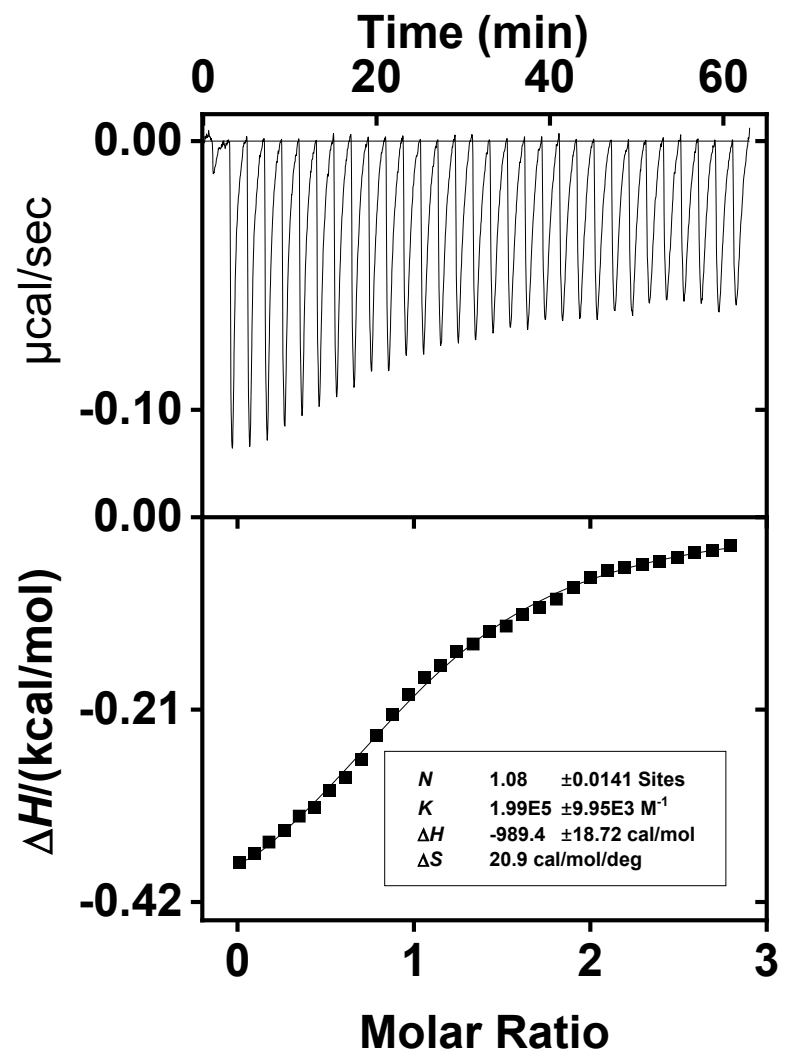

Figure S13. ITC titration curve obtained at 298 K for **NP1b/3a** titrated with cello-oligomer in 10 mM MES buffer at pH 6.5. The data corresponds to entry 7 in Table 1.

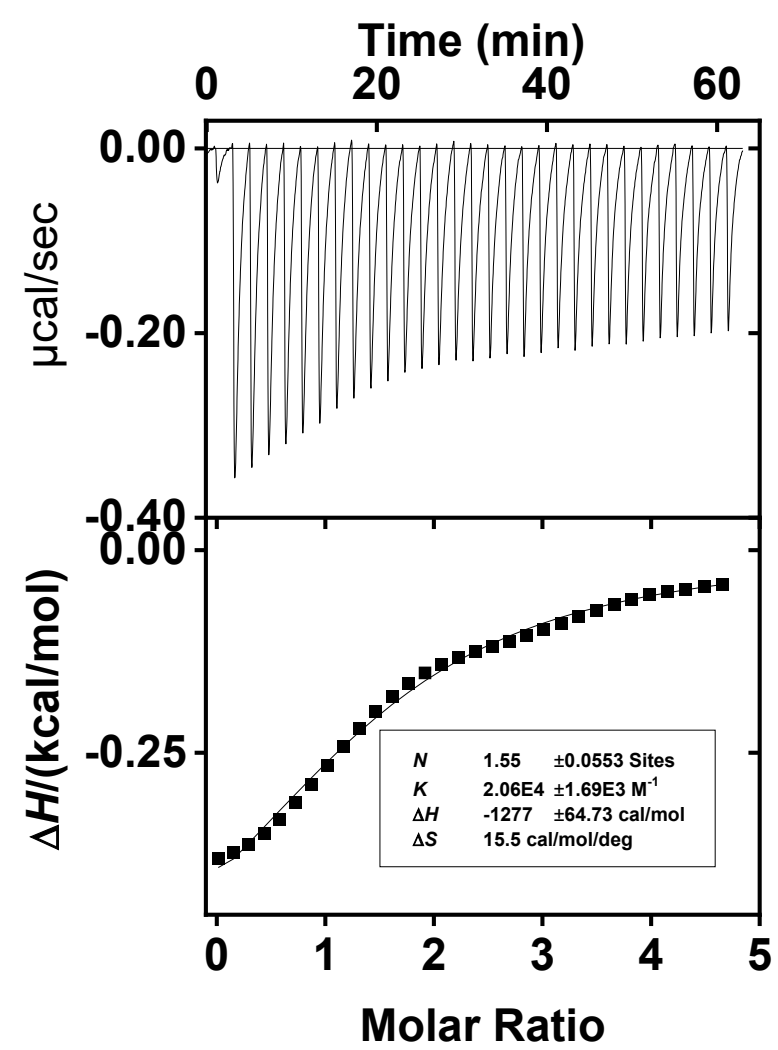

Figure S14. ITC titration curve obtained at 298 K for **NP1c/3a** titrated with glucose in 10 mM MES buffer at pH 6.5. The data corresponds to entry 8 in Table 1.

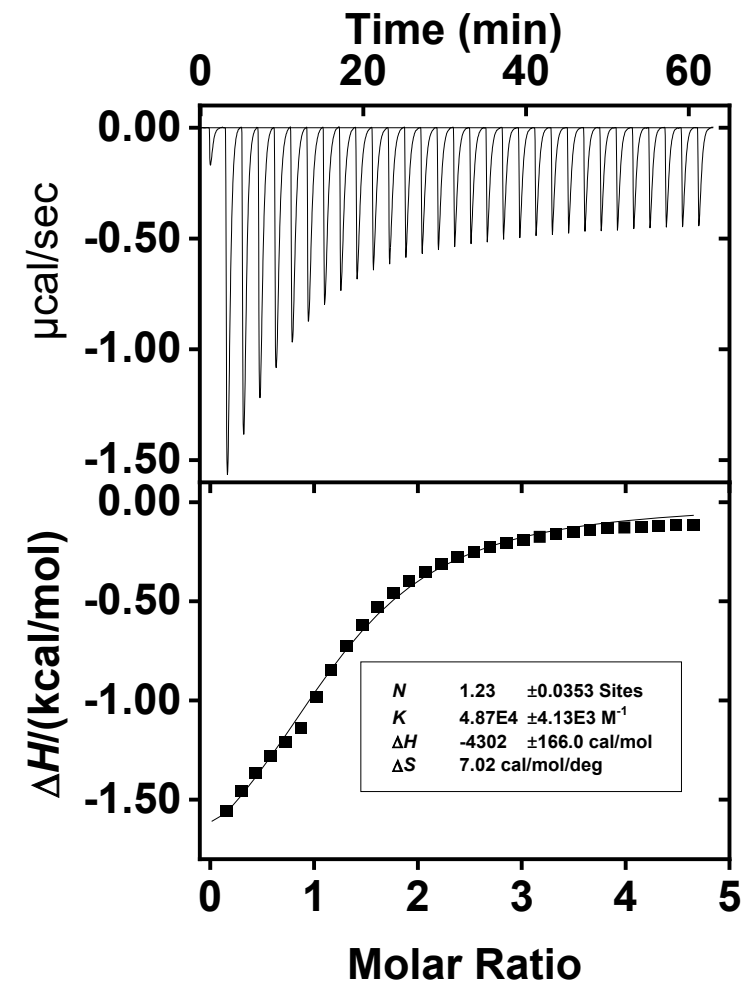

Figure S15. ITC titration curve obtained at 298 K for **NP1c/3a** titrated with cellobiose in 10 mM MES buffer at pH 6.5. The data corresponds to entry 9 in Table 1.

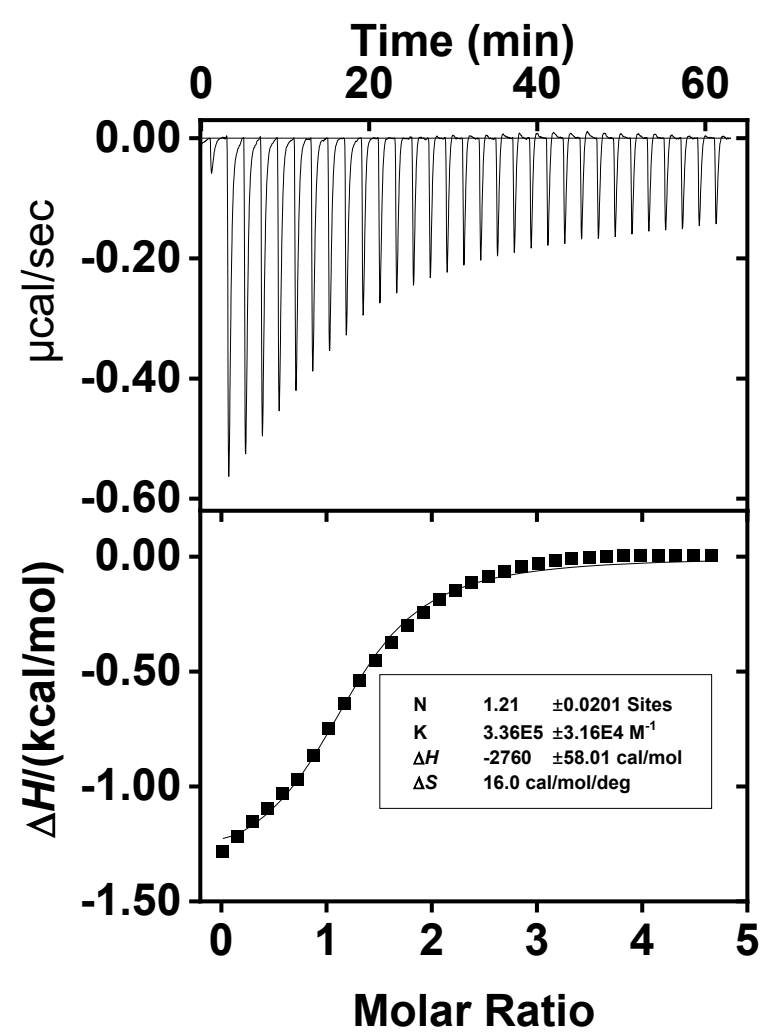

Figure S16. ITC titration curve obtained at 298 K for **NP1c/3a** titrated with cello-oligomer in 10 mM MES buffer at pH 6.5. The data corresponds to entry 10 in Table 1.

6. Hydrolysis of Cellulose and Compounds 8a–f

Hydrolysis of cellulose

In a typical procedure, a reaction mixture containing SigmaCell cellulose (5.0 mg/mL) and the ppropriate catalyst (cellulase or synthetic catalyst at 0.5-5.0 mg/mL) in 1.0 mL of the appropriate buffer was incubated at the indicated temperature for 12 h. All samples were centrifuged at 20,000 RPM before analysis to remove the nano particles. A 50 µL aliquot of the reaction mixture was added to 1.0 mL of a cyanoacetamide solution (25 mM, with 2 mM NaOH) and the mixture was allowed to sit at 95 ºC for 1 h. The concentration of reducing sugar (i.e.,"glucose equivalents") was determined by the absorbance at 275 nm at 25 ℃ using a calibration curve (Figure S8) generated from authentic samples.

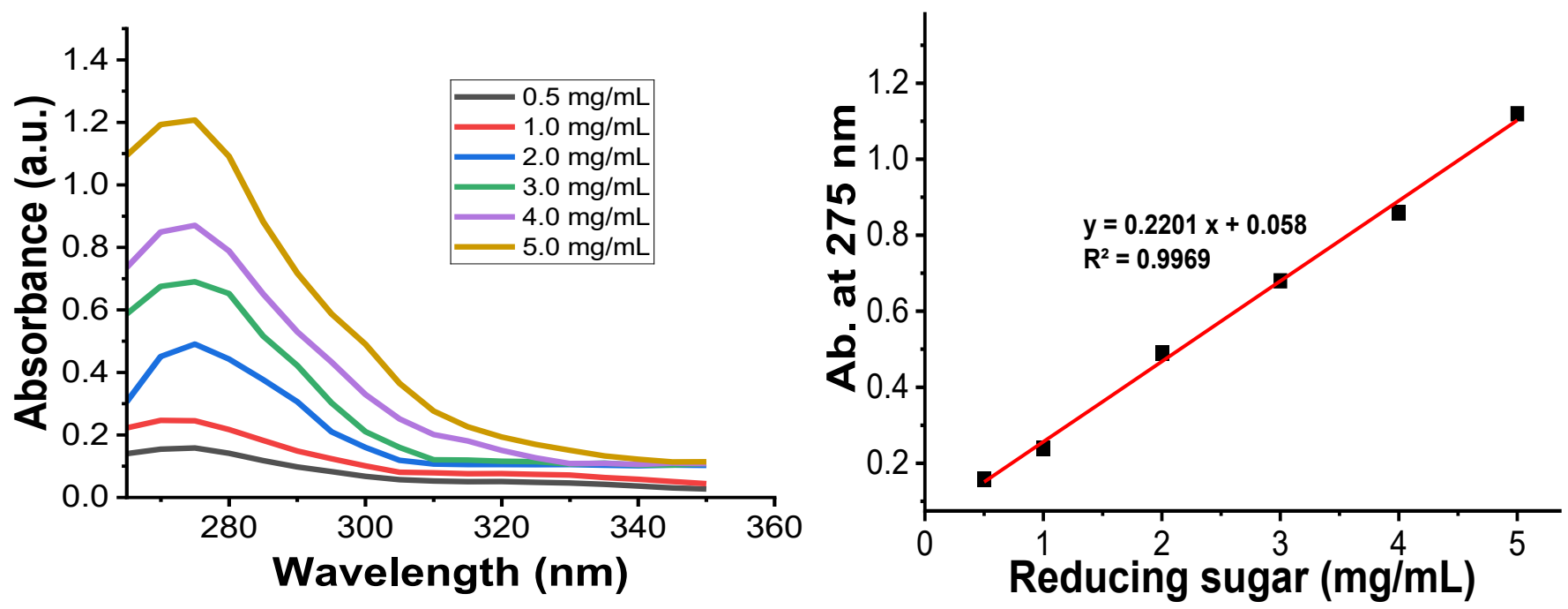

Figure S17. (a) UV/Vis spectra of glucose solutions used to generate the calibration curve for reducing sugar concentration analysis. The glucose samples were obtained by diluting 10 µL, 20 µL, 40 µL, 60 µL, 80 µL, and 100 µL aliquots of a 100 mg/mL glucose stock solution, respectively, with water to a final volume of 2.00 mL. Then 50 µL aliquots of these solutions were added to cyanoacetamide as described above to generate the calibration curve. (b) Calibration curve for reducing sugar generated from absorbance at 275 nm.

LC-MS Characterization of Hydrolysis Products

In a typical procedure, a reaction mixture containing cellulose (5.0 mg/mL) and the appropriate catalyst (cellulase or synthetic catalyst at 2 mg/mL) in 1.0 mL of buffer was incubated at indicated temperatures for 12 h. After cooled to room temperature, the reaction mixture was centrifuged at 20,000 RPM for 10 min to remove the nanoparticle catalyst and residual cellulose before LC-MS analysis. Analysis of reaction mixture after hydrolysis was performed by LC-MS analysis using an Agilent 1200 Series Binary VWD system with an Agilent 6540 UHD Accurate Mass Q-TOF mass detector. Separation of the products was performed on a Thermo Scientific HILIC-LC column (4.6 mm, 150 mm) at 60 ºC and Waters XBridge BEH Amide Column, 130Å, 5 µm, 4.6 mm X 150 mm, 1/pk at 50 ºC. For quantitative analysis, injection volumes were adjusted for the signal intensity to stay within the linear range of the calibration curve. All samples were centrifuged at 20,000 RPM before analysis to remove the nano particles (to avoid column blockage over extended usage). Comparison with non-centrifuged samples showed no change in glucose concentration during the centrifugation step. The mobile phase was a mixture of acetonitrile and water, with 0.1% formic acid. Product peaks were identified by a high-resolution mass detector in the positive ion mode.

Calibration for substrates

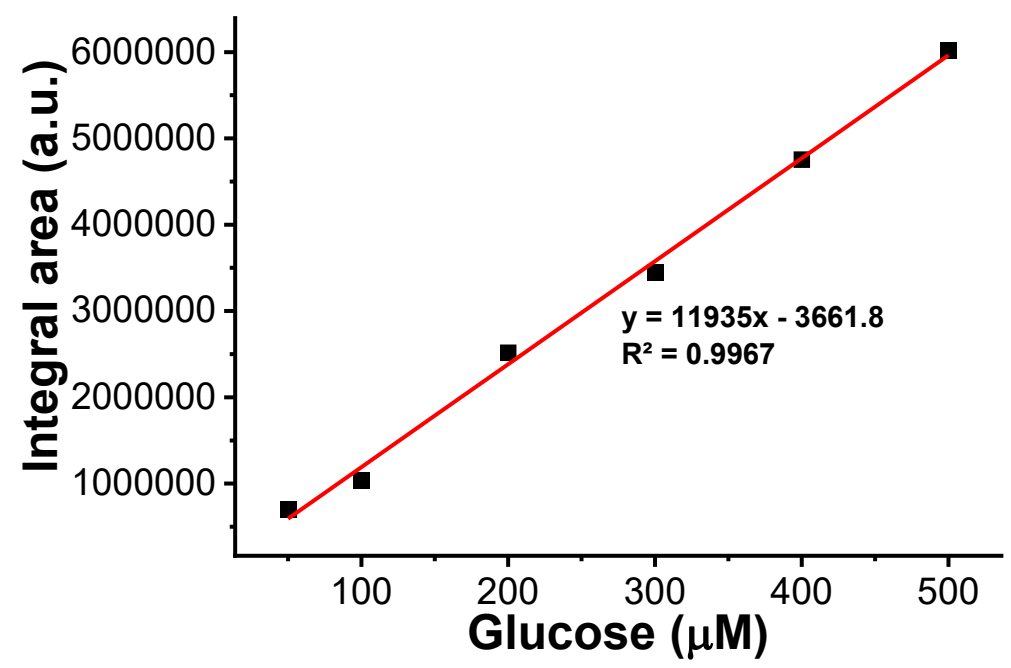

Figure S18. Calibration curve of glucose generated from authentic samples by LC-MS.

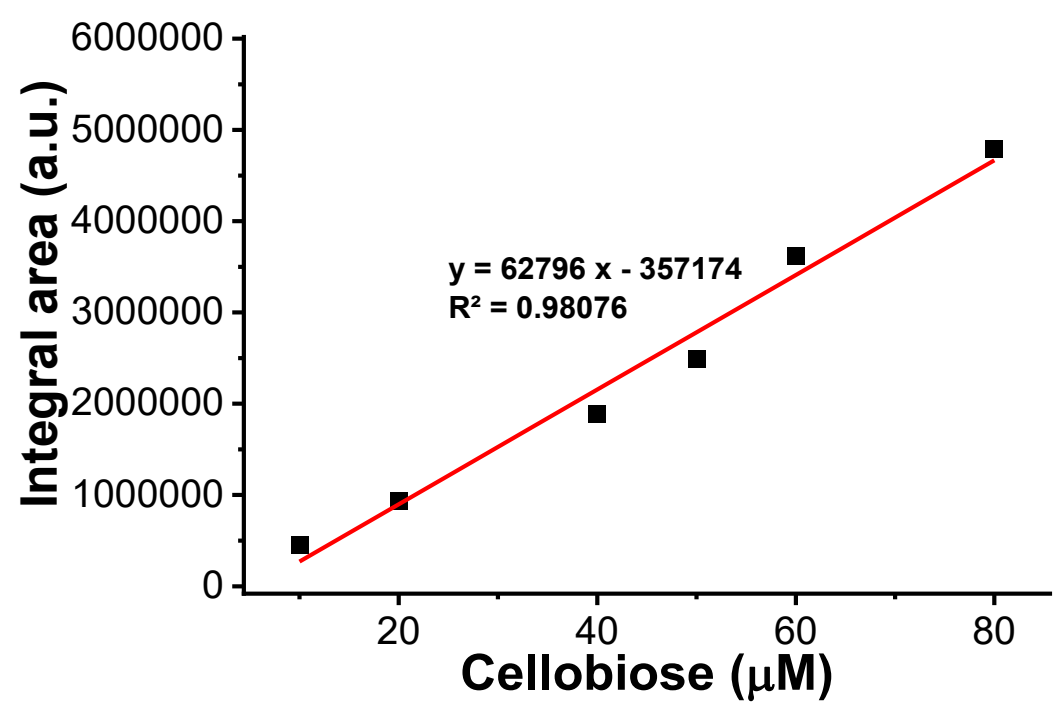

Figure S19. Calibration curve of cellobiose generated from authentic samples by LC-MS.

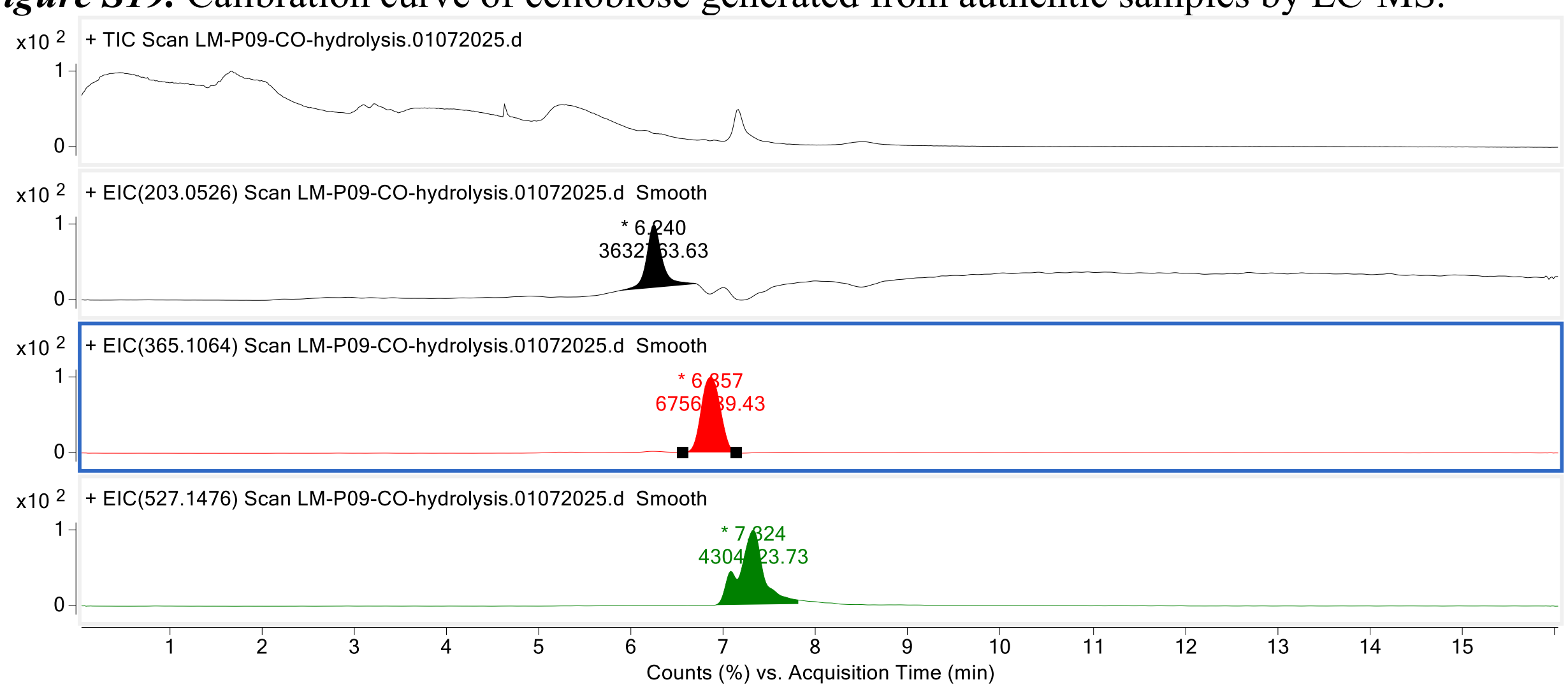

Figure S20. A representative LC-MS trace for the reaction mixture of cellulose hydrolysis catalyzed by NP**1c**/**3a** at 90 ºC for 12 h. Extracted ion chromatography (EIC) spectra indicates that glucose (black) was observed at 5.86 min and cellobiose (red) at 6.78 min. Glucose and cellobiose concentration in the sample is 0.8 mg/mL and 0.3 mg/mL. Other oligomers were not detected. The experiments were repeated three times and gave similar results.

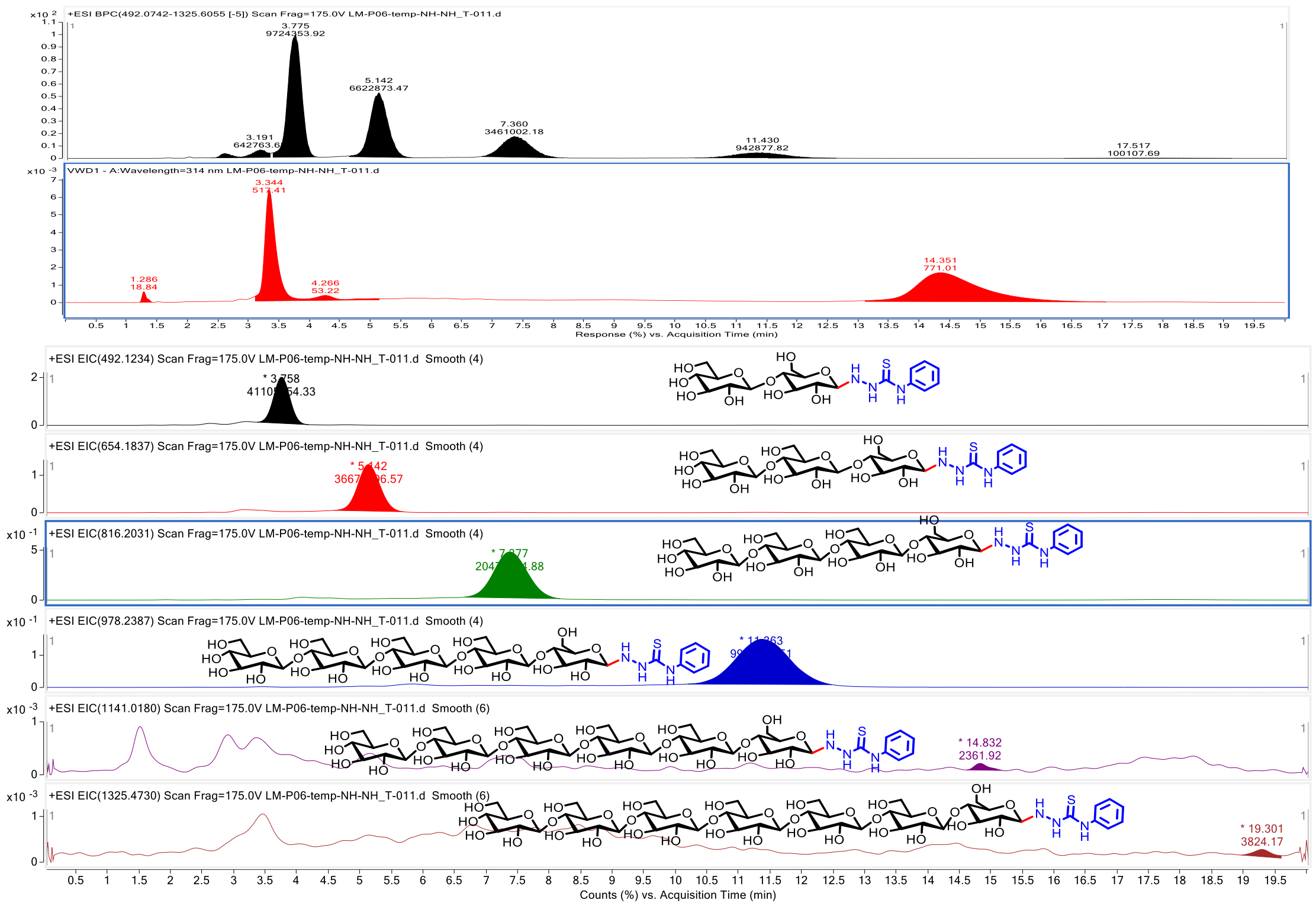

Figure S21. Representative LC-MS chromatogram of template **1c**. The reaction was conducted with 4-phenylthiosemicarbazide and cello-oligomers in a methanol/acetone mixture (1:5, v/v) at 60 °C for 12 hours. Extracted ion chromatograms (EICs) indicate the formation of products DP2–DP7 (hydrazine thiourea), consistent with a sequential transformation pathway mediated by hydrazine thiourea under the specified reaction conditions.

Table S2. Michaelis-Menten kinetic parameters for phenyl β-D-glucopyranoside derivatives (**8a-8f**) using **NP′1a/3a**.[a]

entry	substrates	pK_a	log(k_{cat}/k_m) ($M^{-1}sec^{-1}$)	k_{cat}/k_m ($M^{-1}sec^{-1}$)	K_m (μM)
1	**8a**	3.96	1.78	60.7	20.9
2	**8b**	5.43	1.58	38.9	25.1
3	**8c**	7.15	1.34	22	34.2
4	**8d**	7.23	1.32	21.1	32.6
5	**8e**	8.36	0.65	4.5	65.8
6	**8f**	10	0.11	1.3	82.6

[a] Michaelis–Menten kinetics for the hydrolysis of different substrates(**8a-8f**) by **NP′1a/3a** in 25 mM MES buffer at 40 °C and pH 6.5. [**NP′1a/3a**] = 10 μM.

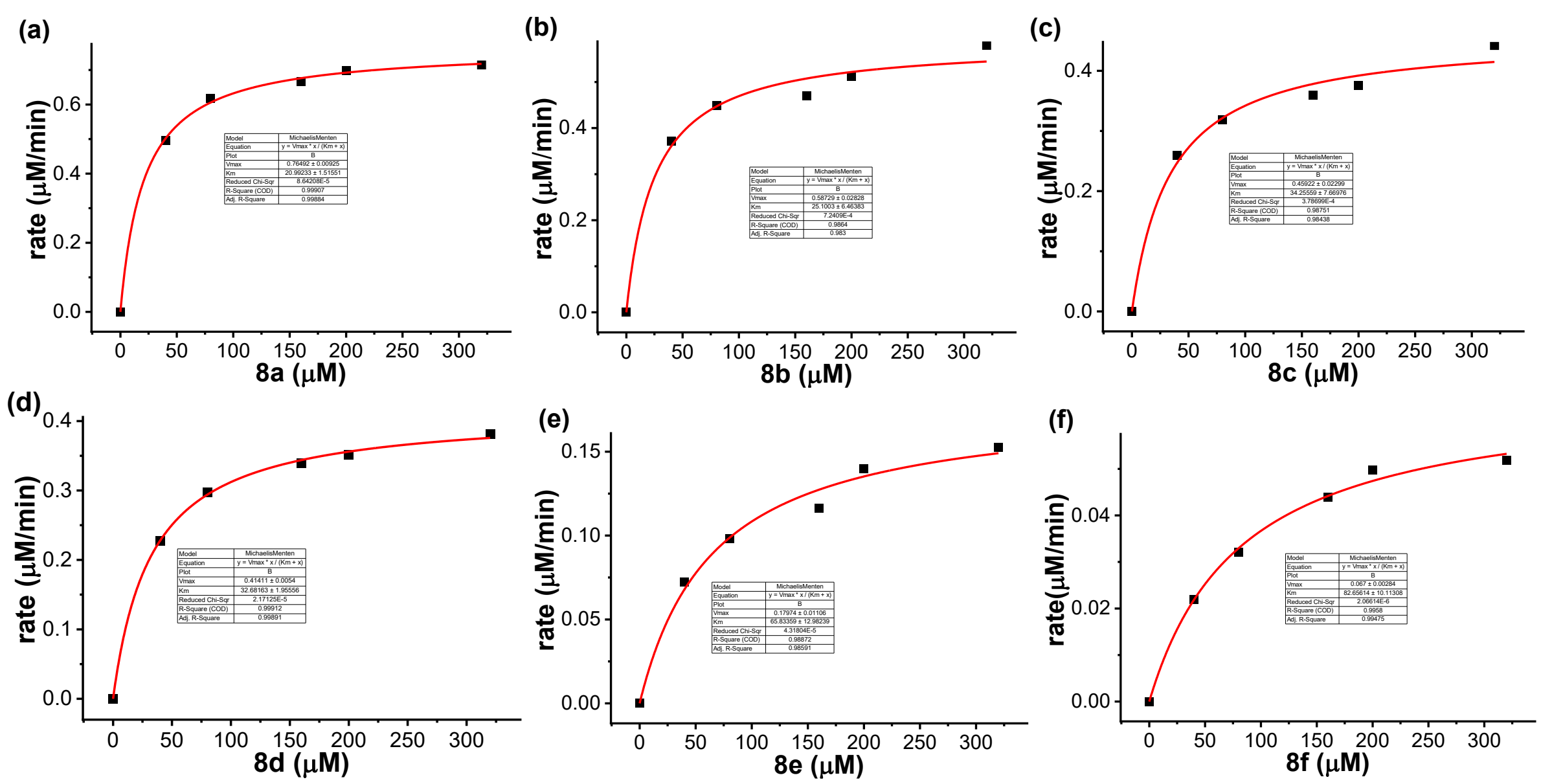

Figure S22. Michaelis–Menten plots for the hydrolysis of phenyl β-D-glucopyranoside derivatives **8a–8f** catalyzed by **NP′1a/3a**. Reactions were performed in 25 mM MES buffer (pH 6.5) at 40 °C with [**NP′1a/3a**] = 10 μM. Initial rates were measured at varying substrate concentrations and fitted to the Michaelis–Menten equation. The plots correspond to the following substrates: (a) **8a**, (b) **8b**, (c) **8c**, (d) **8d**, (e) **8e**, and (f) **8f**.

7. NMR Spectra of Key Compounds

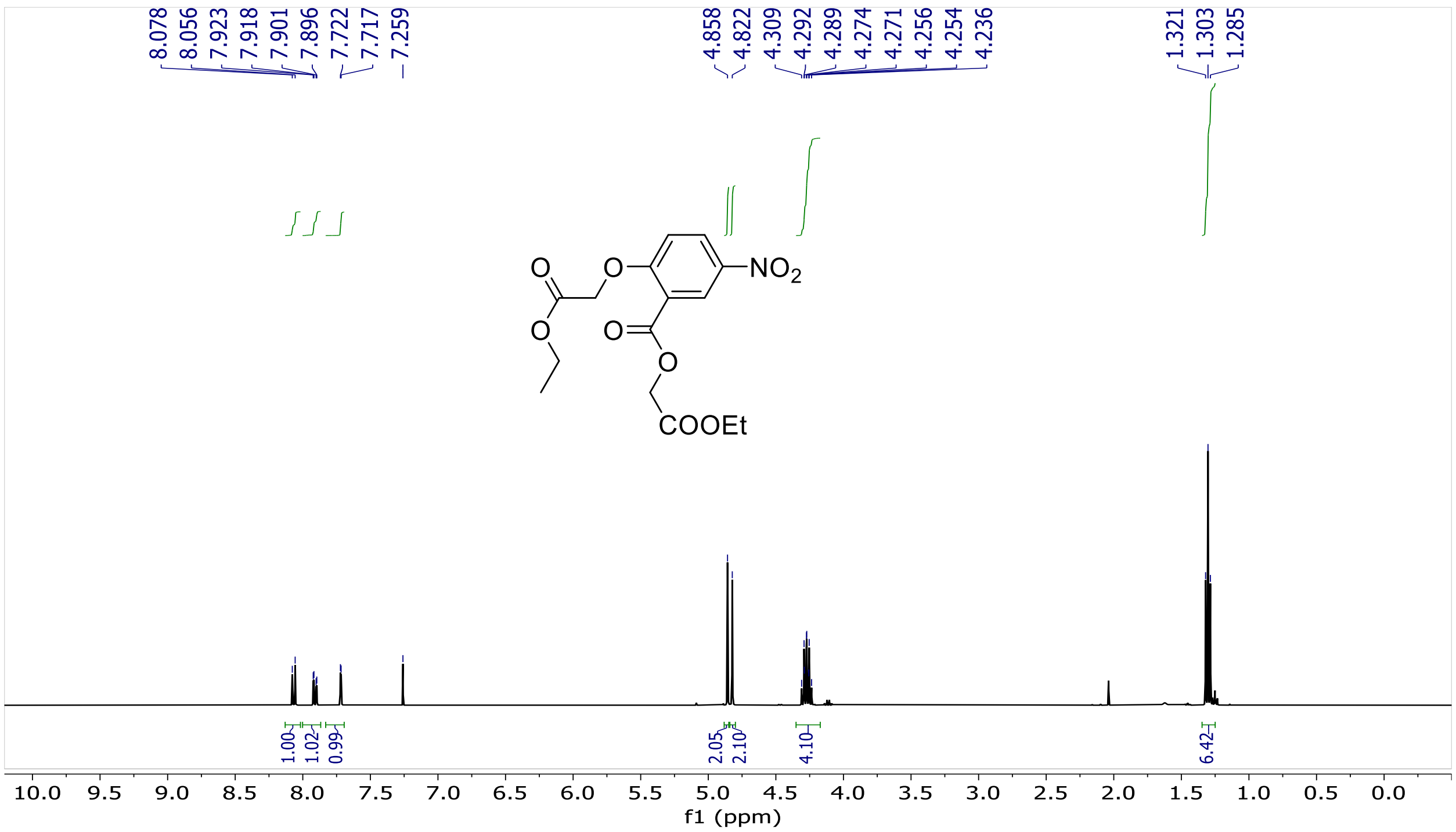

Figure S23. ^{1}H NMR (400 MHz, $CDCl_3$) spectrum of compound **9** in $CDCl_3$.

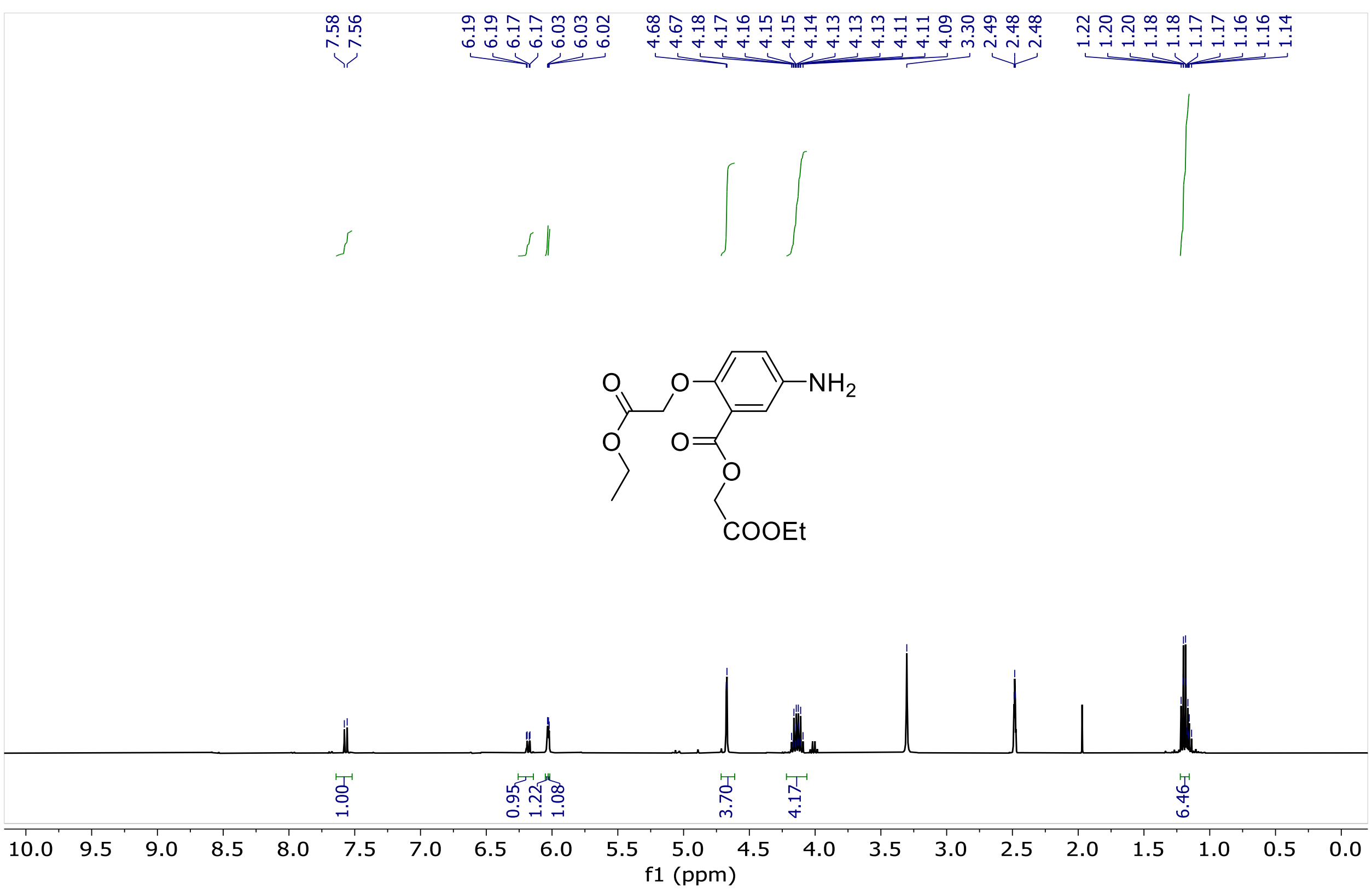

Figure S24. ^{1}H NMR (400 MHz, DMSO-d6) spectrum of compound **10** in DMSO-d6.

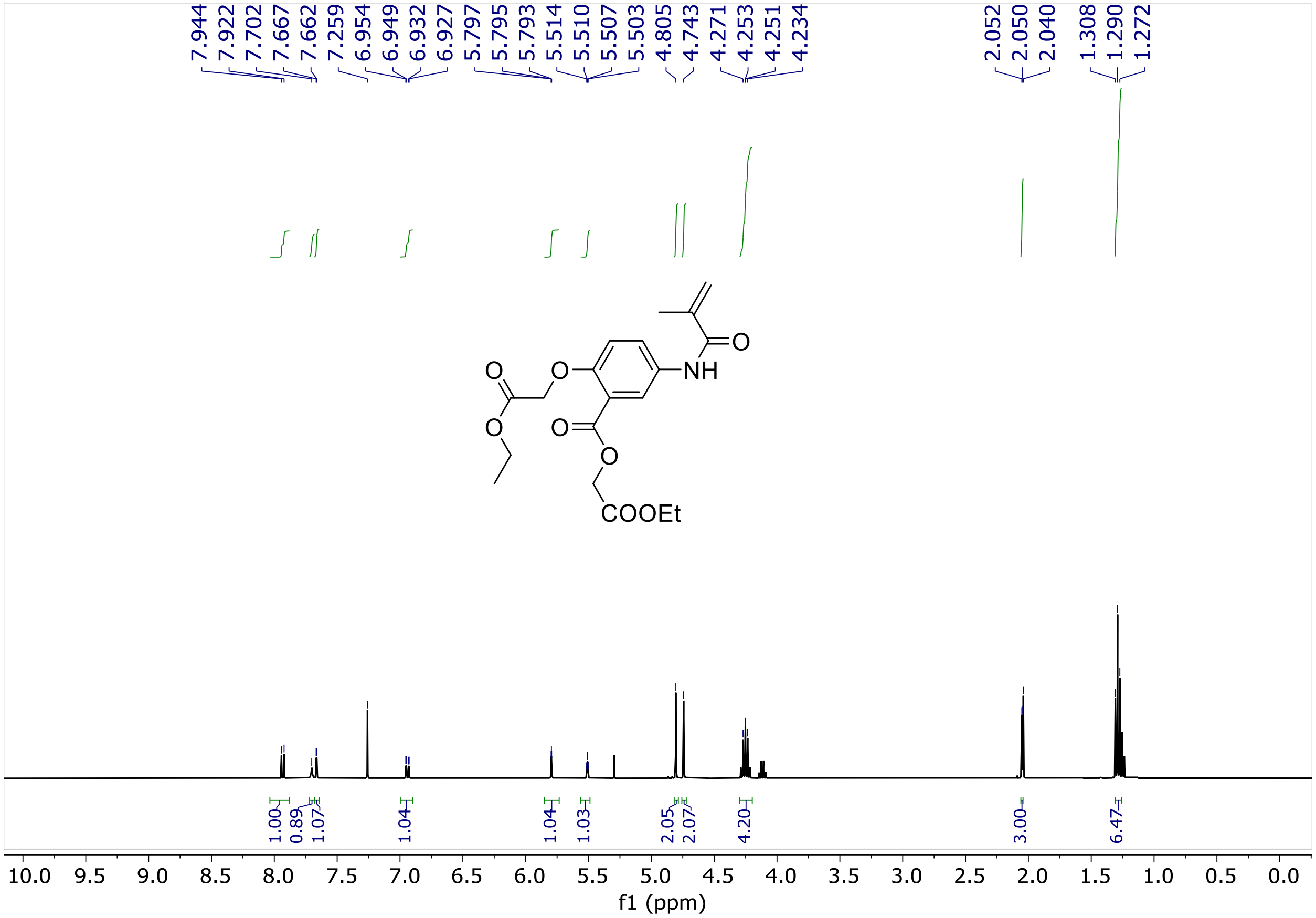

Figure S25. ^{1}H NMR (400 MHz, $CDCl_3$) spectrum of compound **11** in $CDCl_3$.

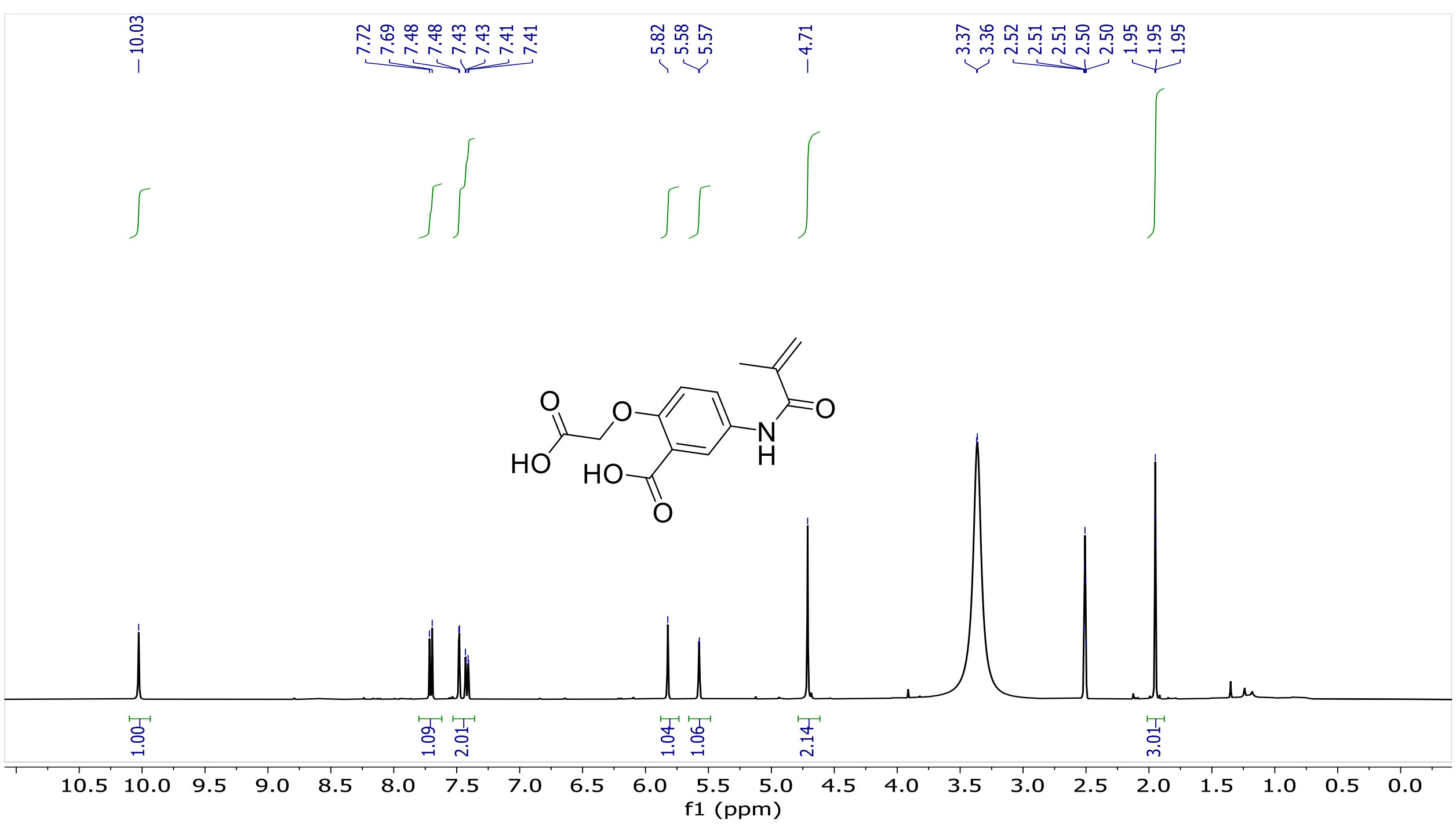

Figure S26. ^{1}H NMR (600 MHz, DMSO-d6) spectrum of compound **3a** in DMSO-d6.

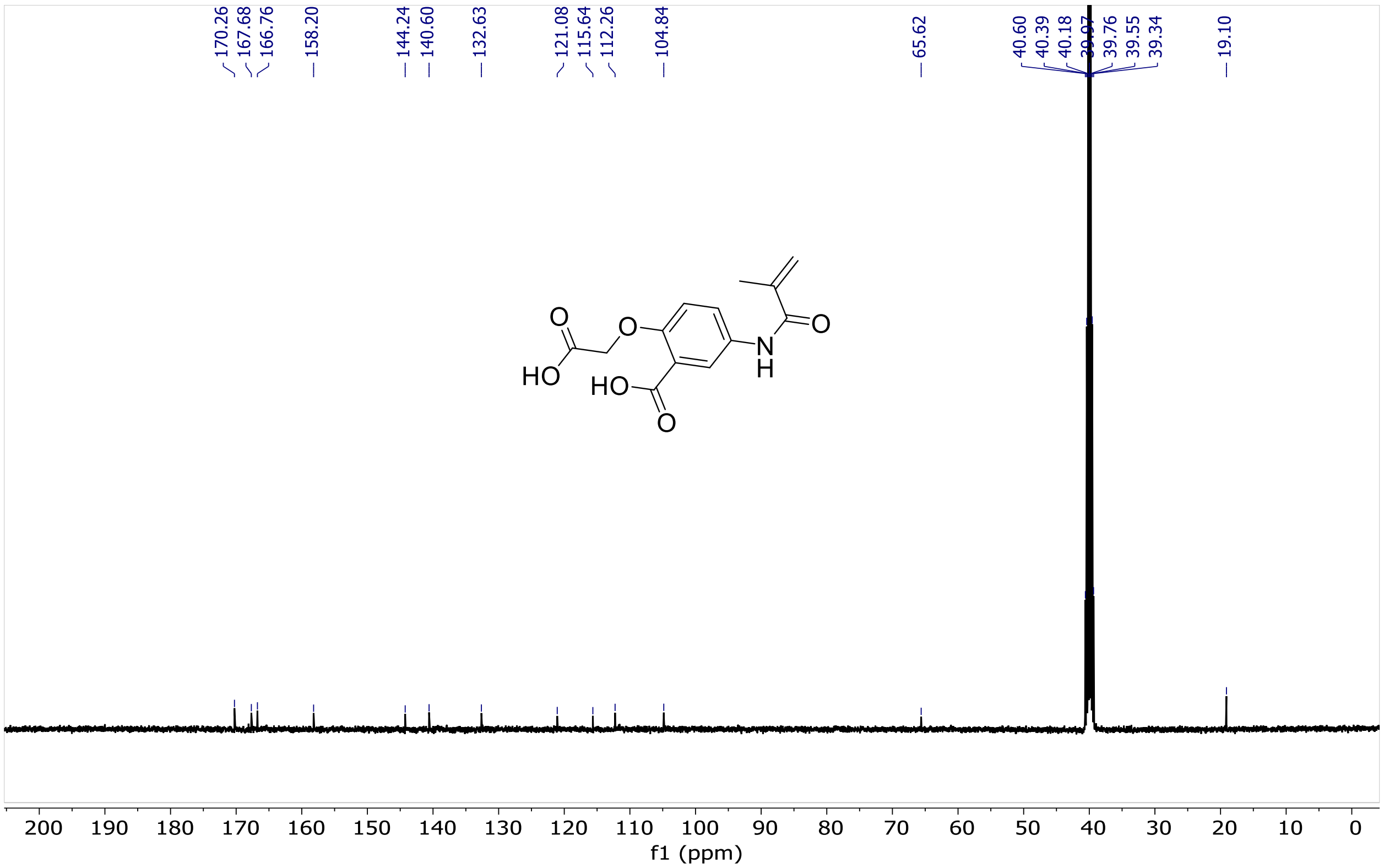

Figure S27. ^{13}C NMR (600 MHz, DMSO-d6) spectrum of compound **3a** in DMSO-d6.

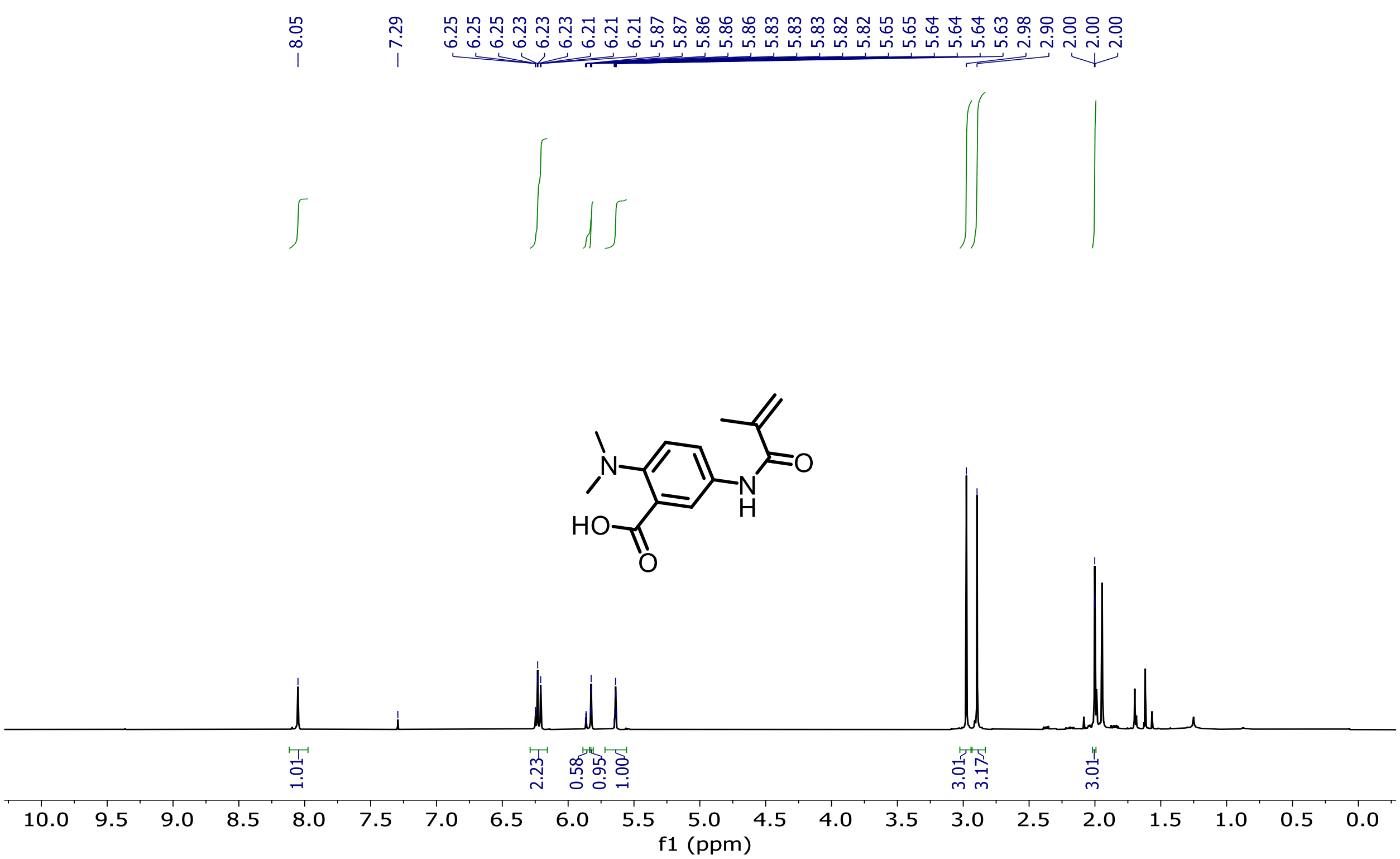

Figure S28. ^{1}H NMR (600 MHz, $CDCl_3$) spectrum of compound **3b** in $CDCl_3$.

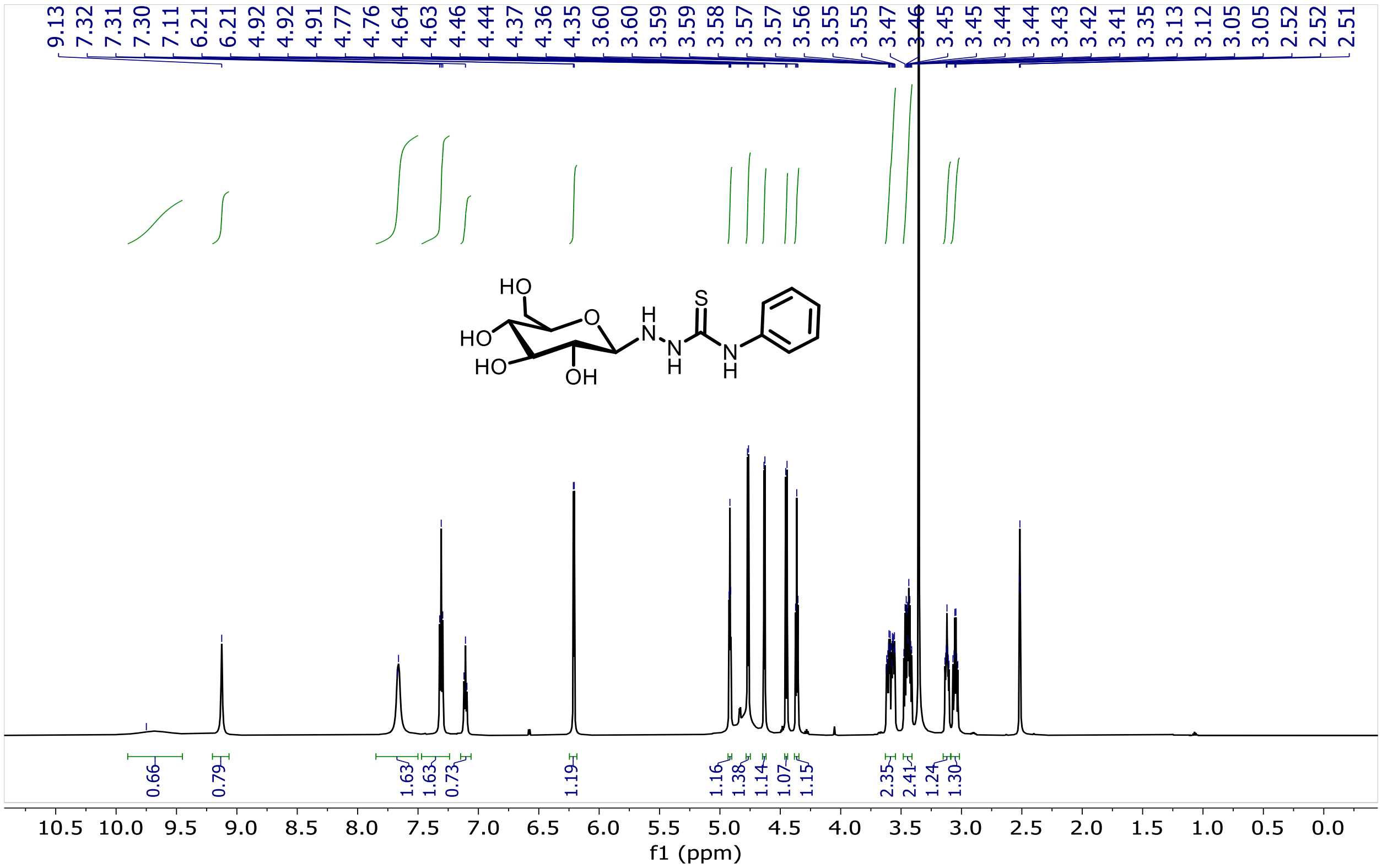

Figure S29. ^{1}H NMR (600 MHz, DMSO-d6) spectrum of compound **1a** in DMSO-d6.

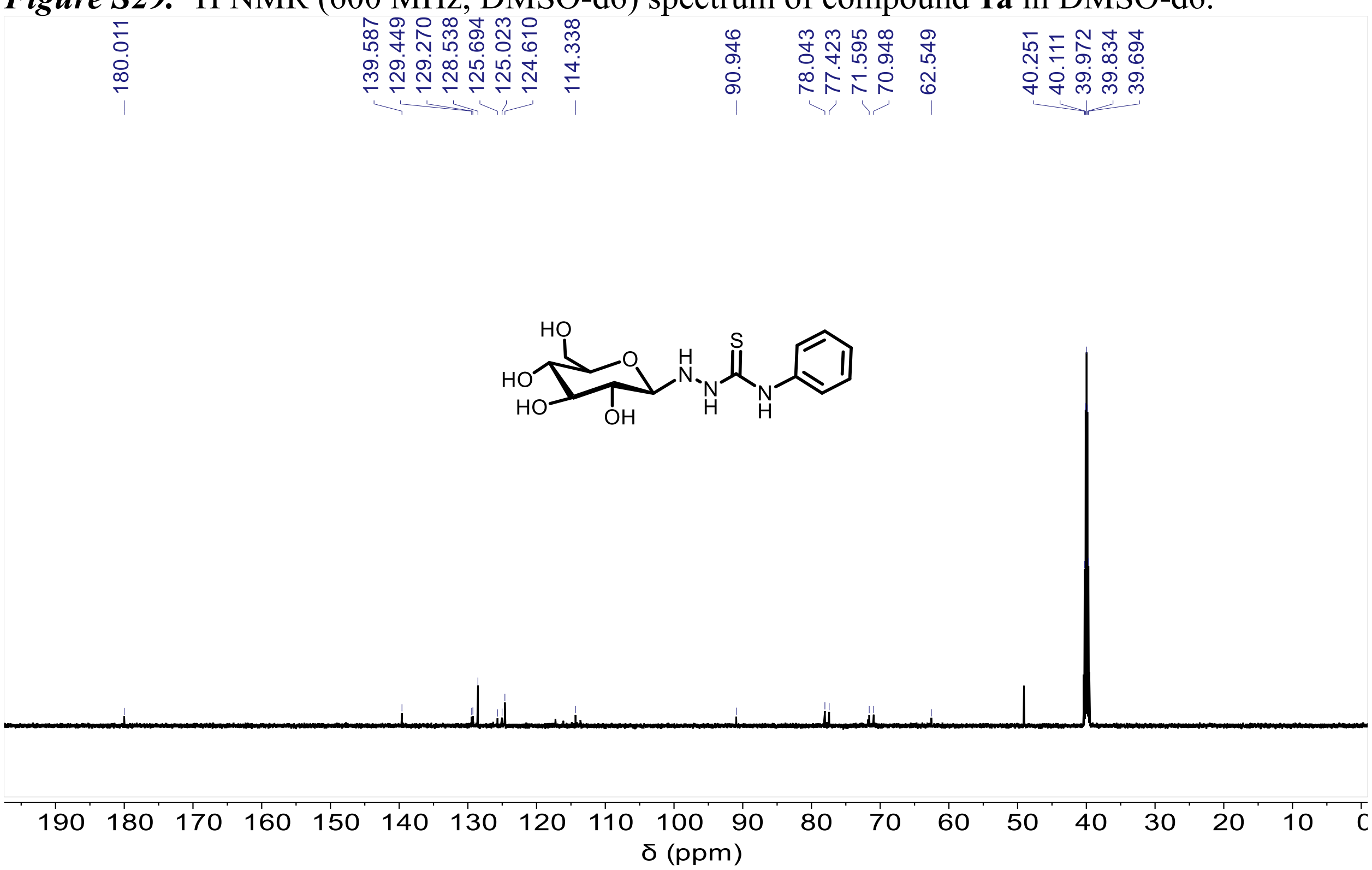

Figure S30. ^{13}C NMR (151 MHz, DMSO-d6) spectrum of compound **1a** in DMSO-d6.

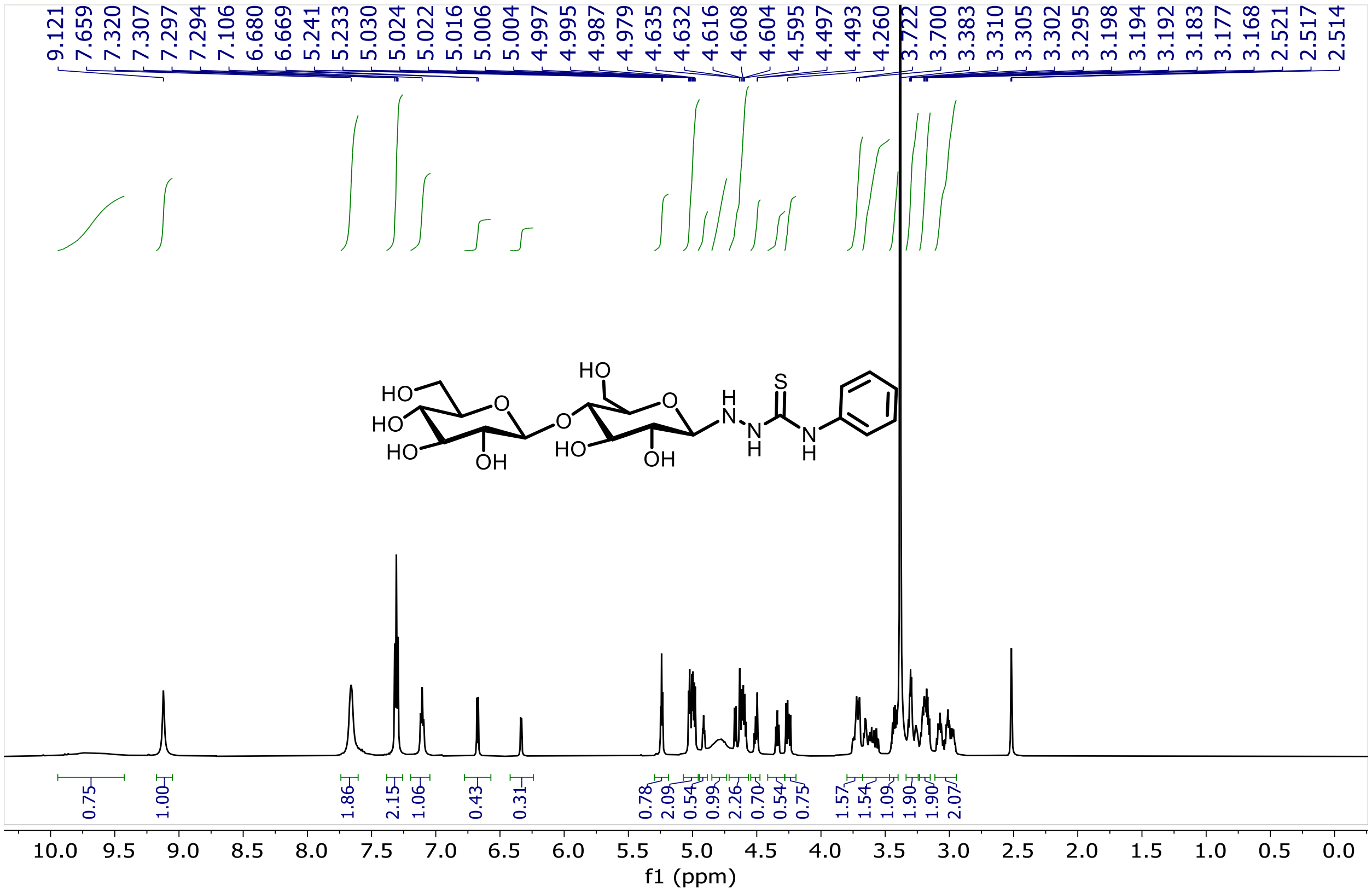

Figure S31. ^{1}H NMR (600 MHz, DMSO-d6) spectrum of compound **1b** in DMSO-d6.

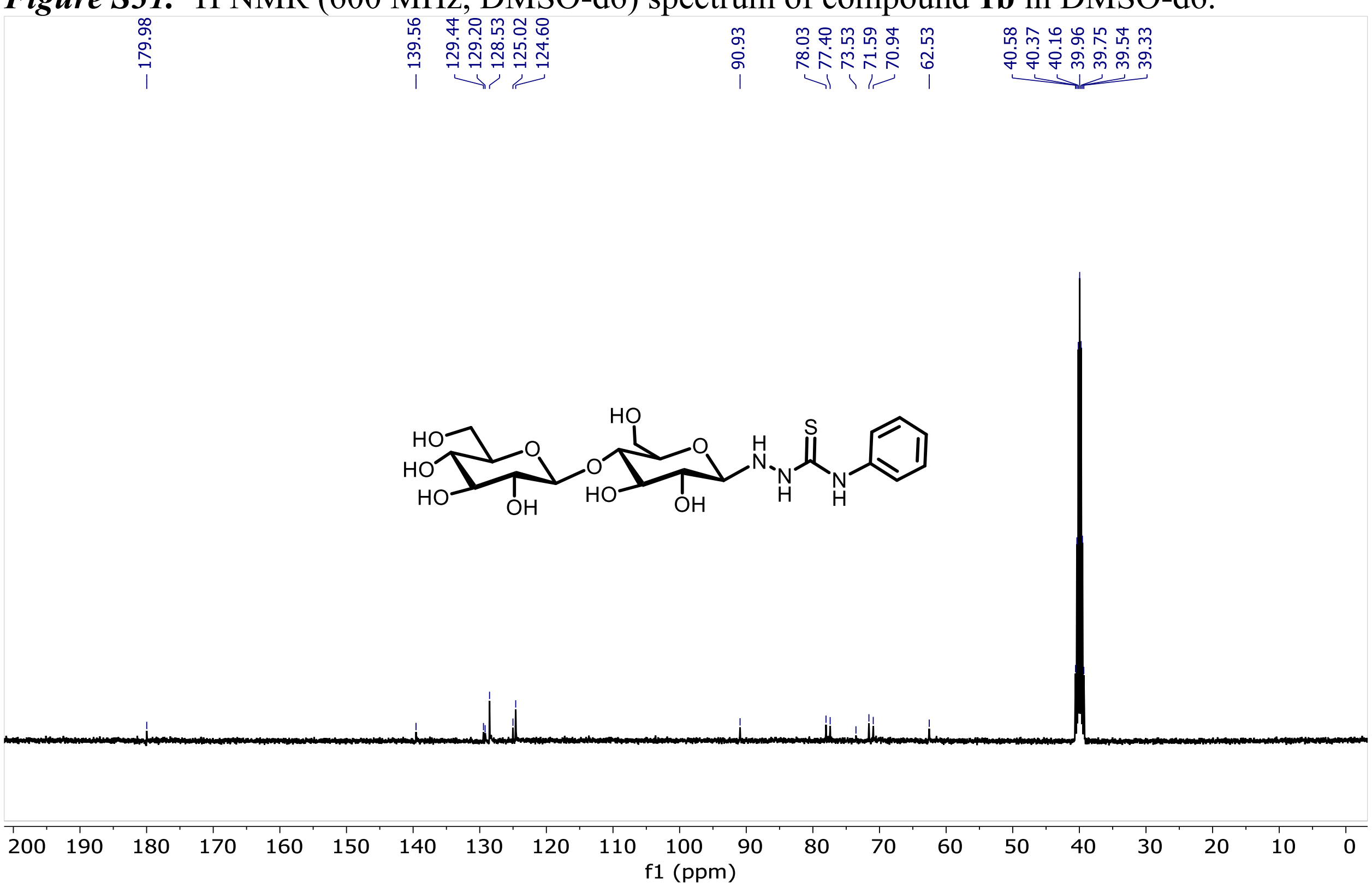

Figure S32. ^{13}C NMR (151 MHz, DMSO-d6) spectrum of compound **1b** in DMSO-d6.

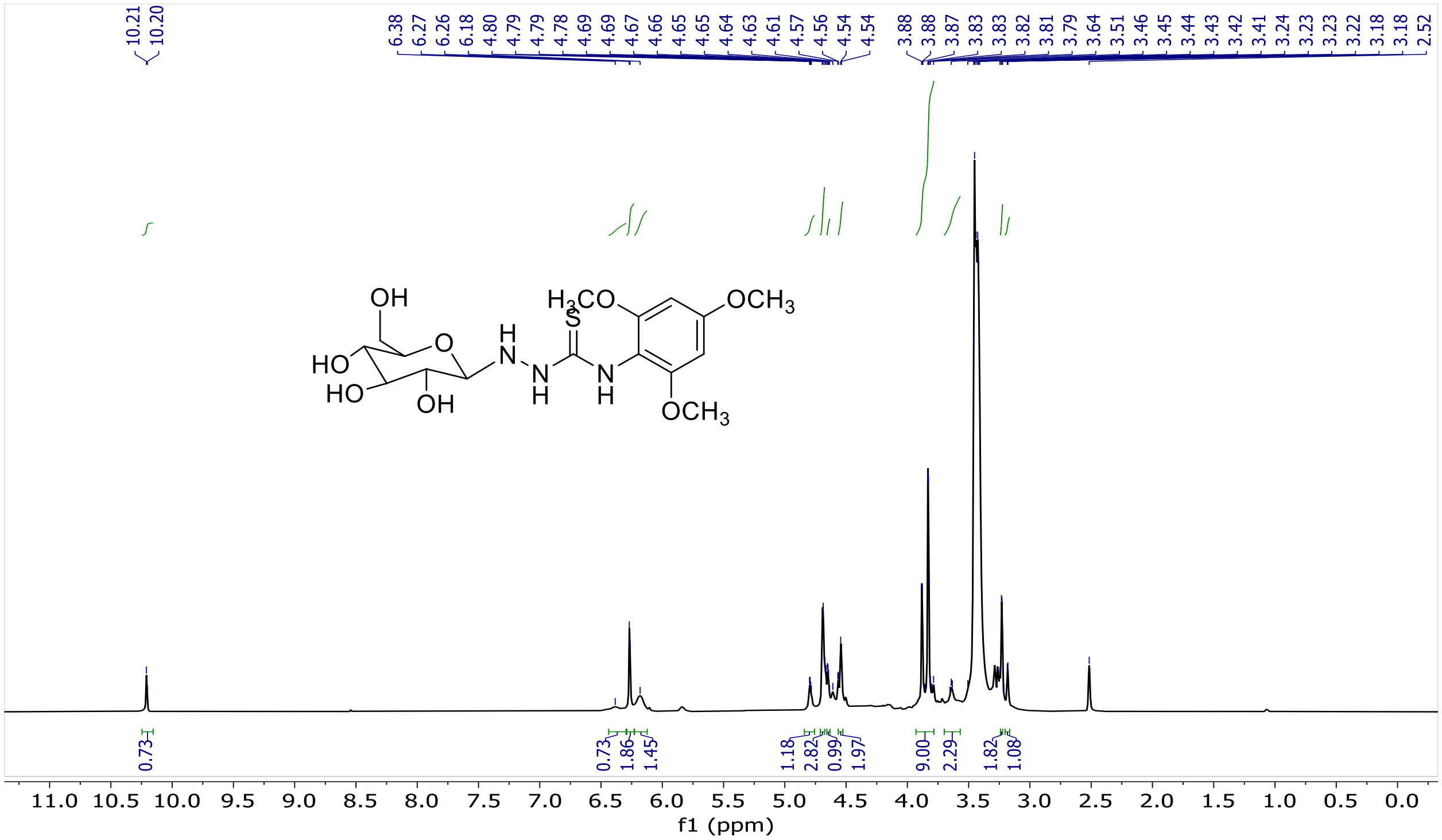

Figure S33. ^{1}H NMR (400 MHz, DMSO-d6) spectrum of template **1'** in DMSO-d6.

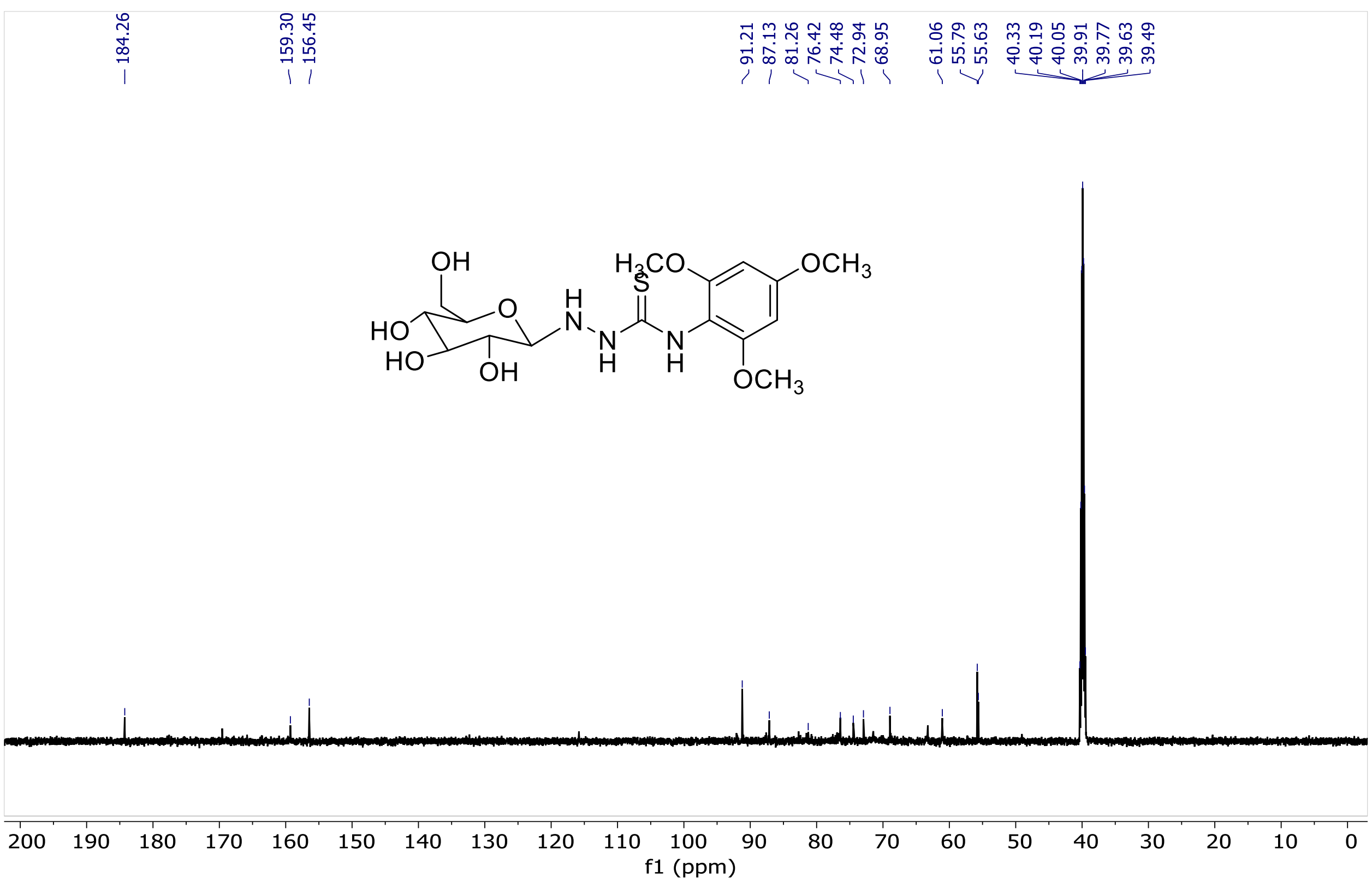

Figure S34. ^{13}C NMR (101 MHz, DMSO-d6) spectrum of template **1'** in DMSO-d6.

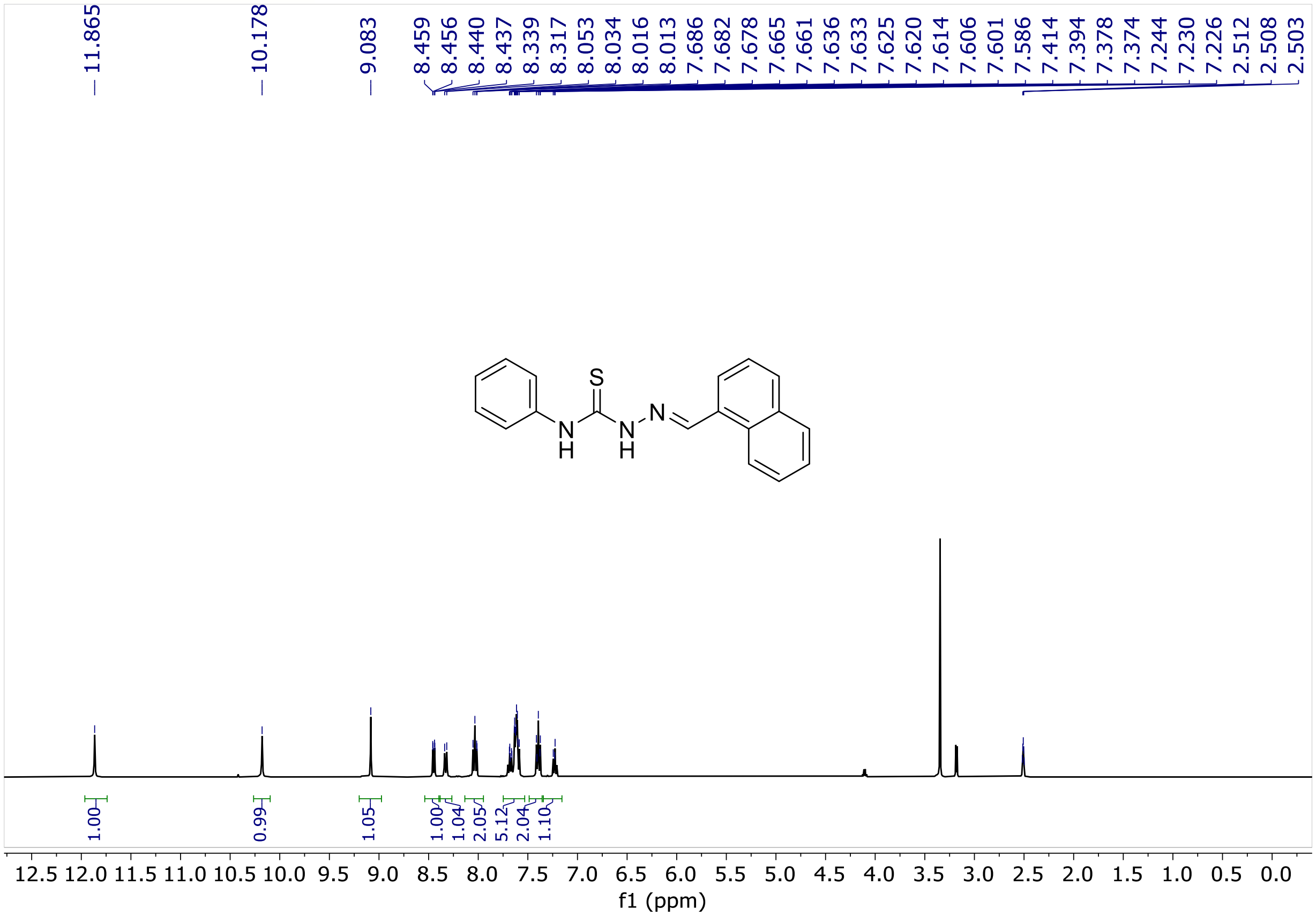

Figure S35. ^{1}H NMR (400 MHz, DMSO-d6) spectrum of compound **13** in DMSO-d6.

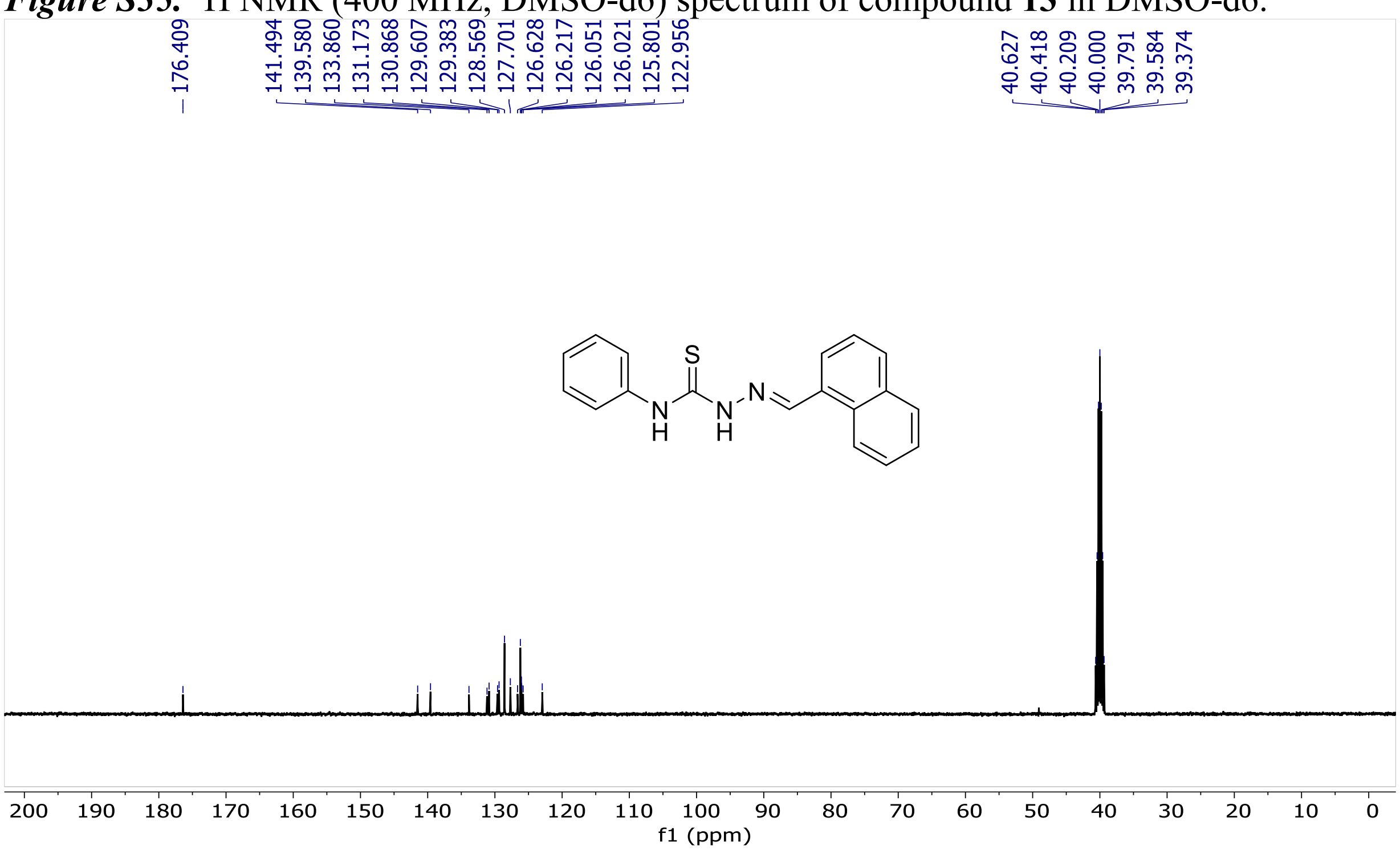

Figure S36. ^{13}C NMR (101 MHz, DMSO-d6) spectrum of compound **13** in DMSO-d6.

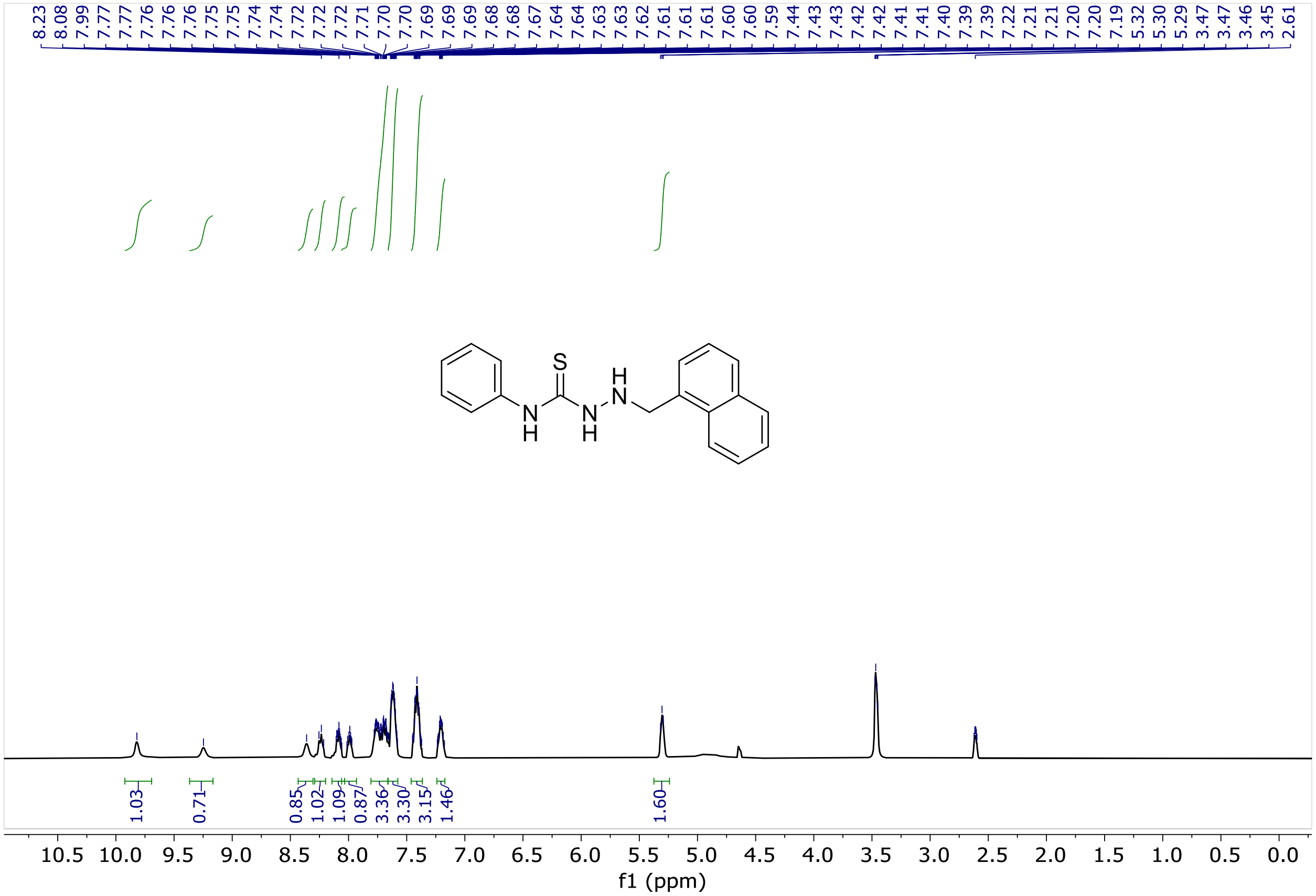

Figure S37. ^{1}H NMR (400 MHz, DMSO-d6) spectrum of compound **7a** in DMSO-d6.

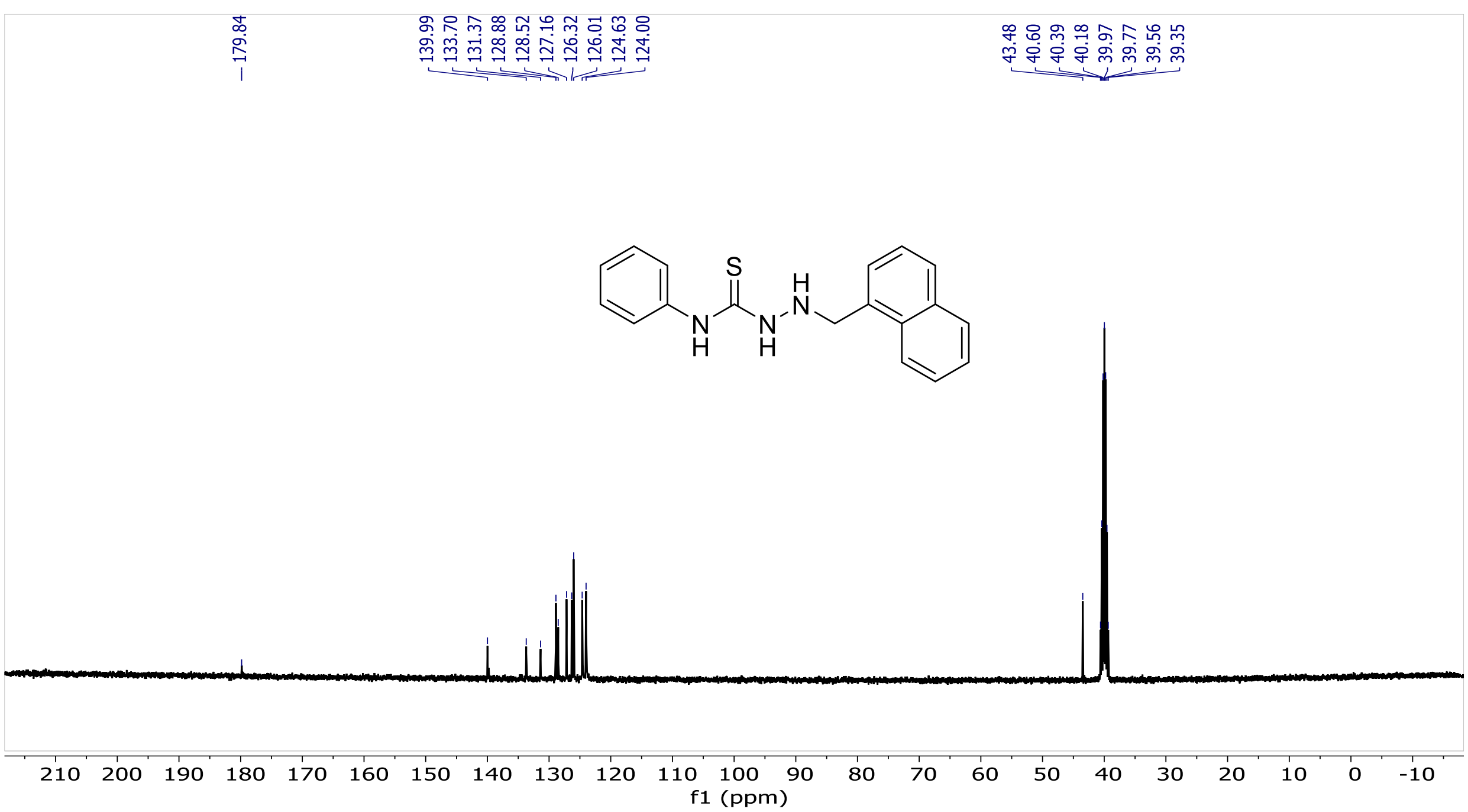

Figure S38. ^{13}C NMR (101 MHz, DMSO-d6) spectrum of compound **7a** in DMSO-d6.

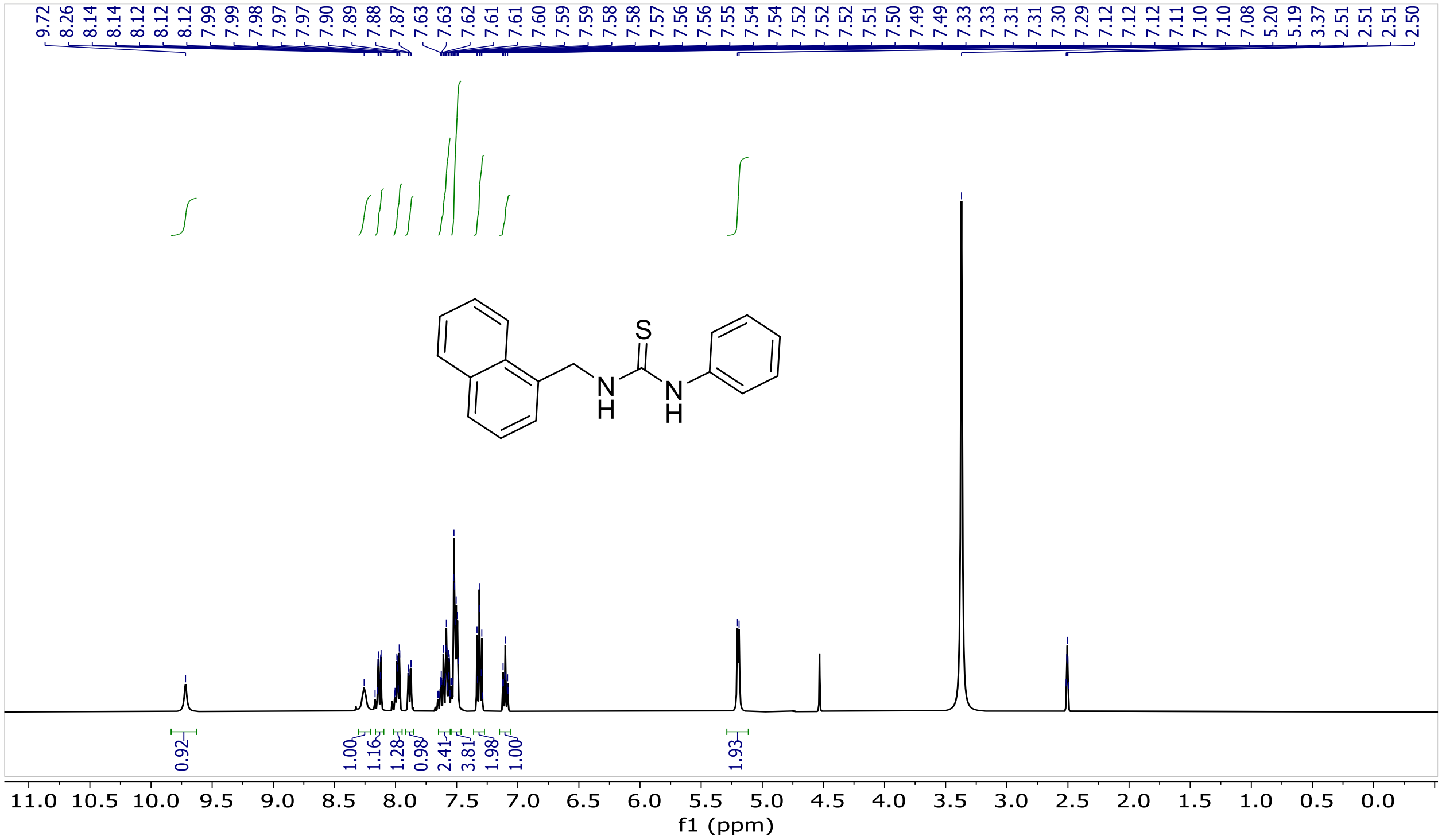

Figure S39. ^{1}H NMR (400 MHz, DMSO-d6) spectrum of compound **7b** in DMSO-d6.

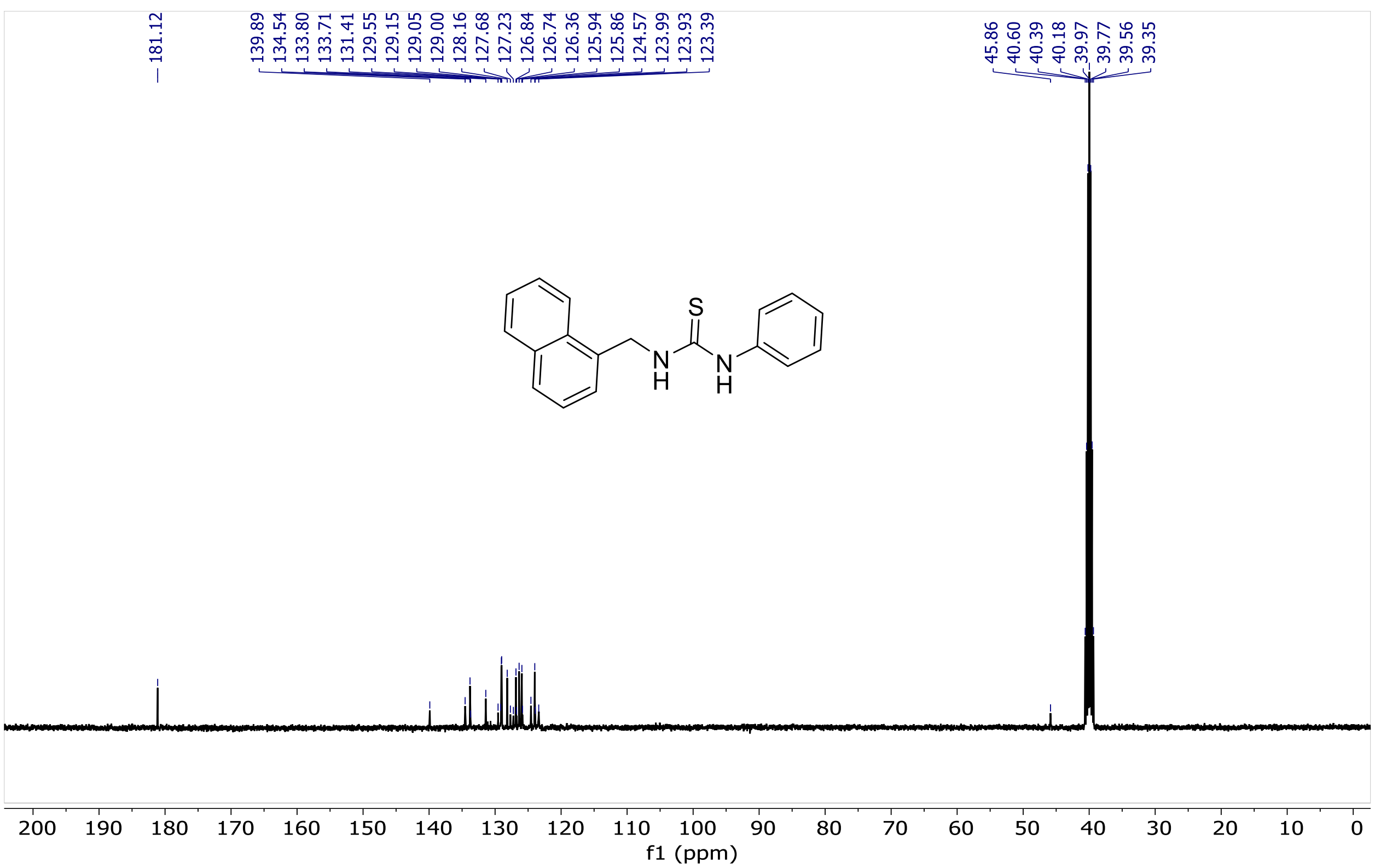

Figure S40. ^{13}C NMR (101 MHz, DMSO-d6) spectrum of compound **7b** in DMSO-d6.

8. ESI-MS Spectra of Key Compounds

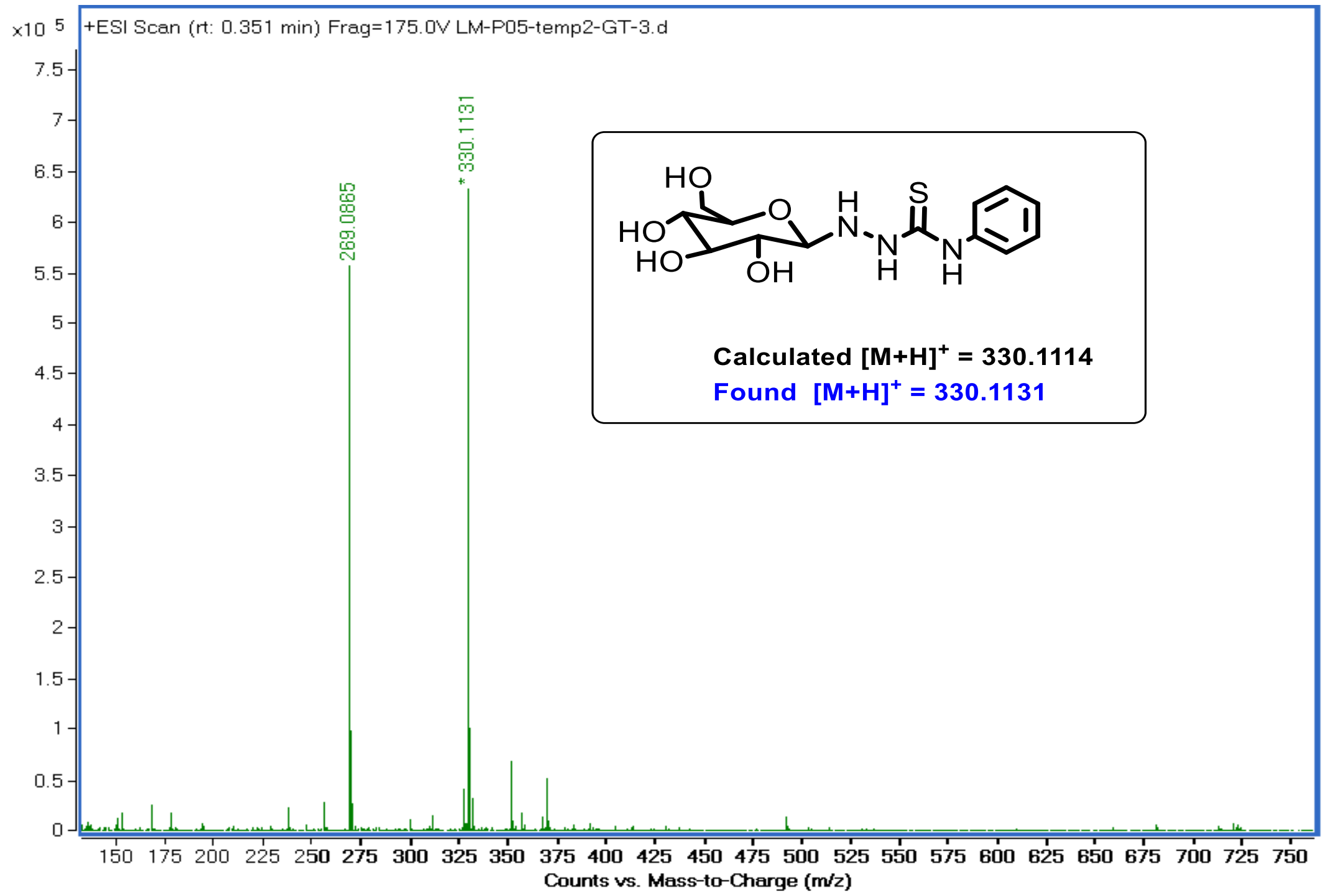

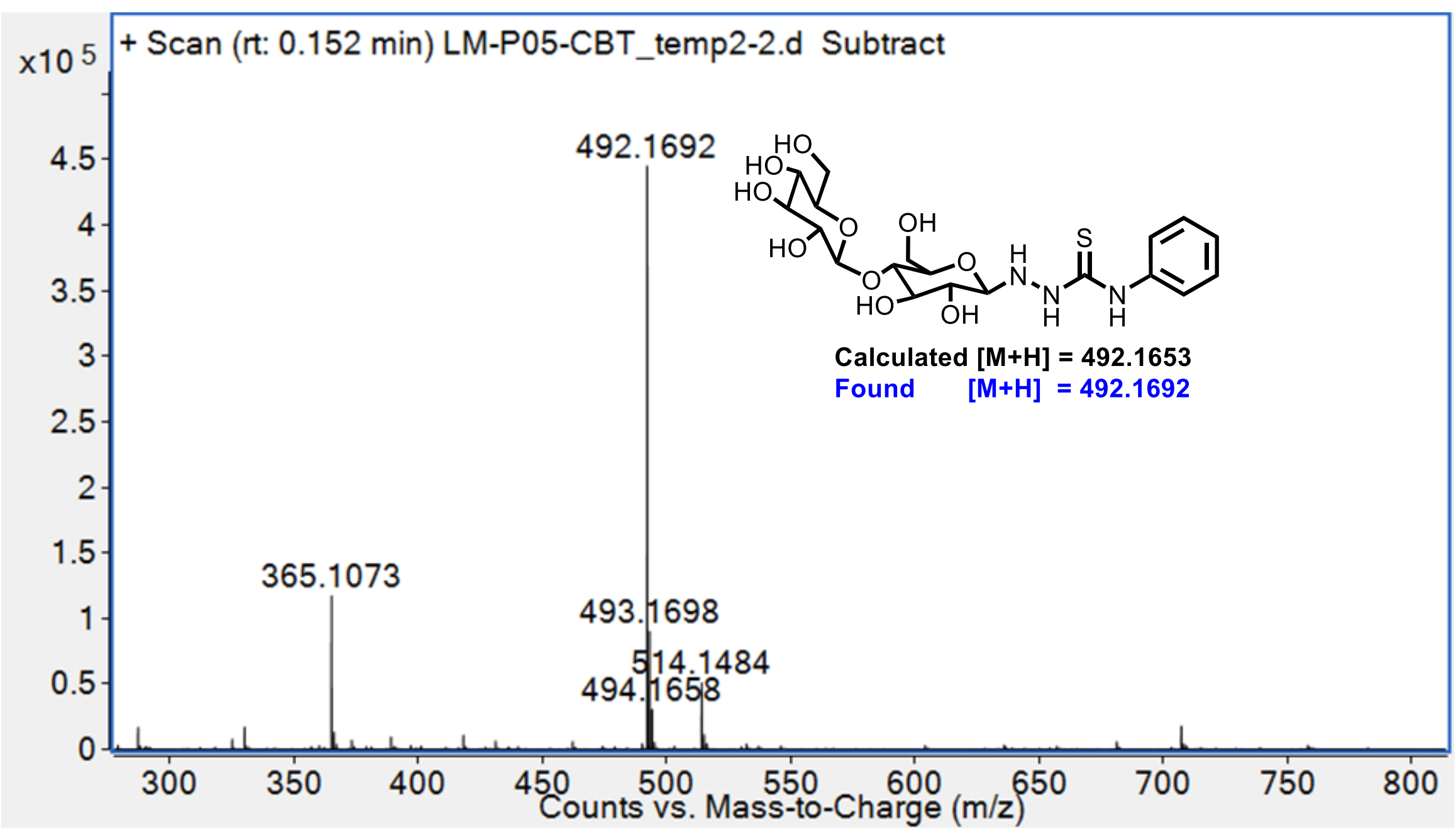

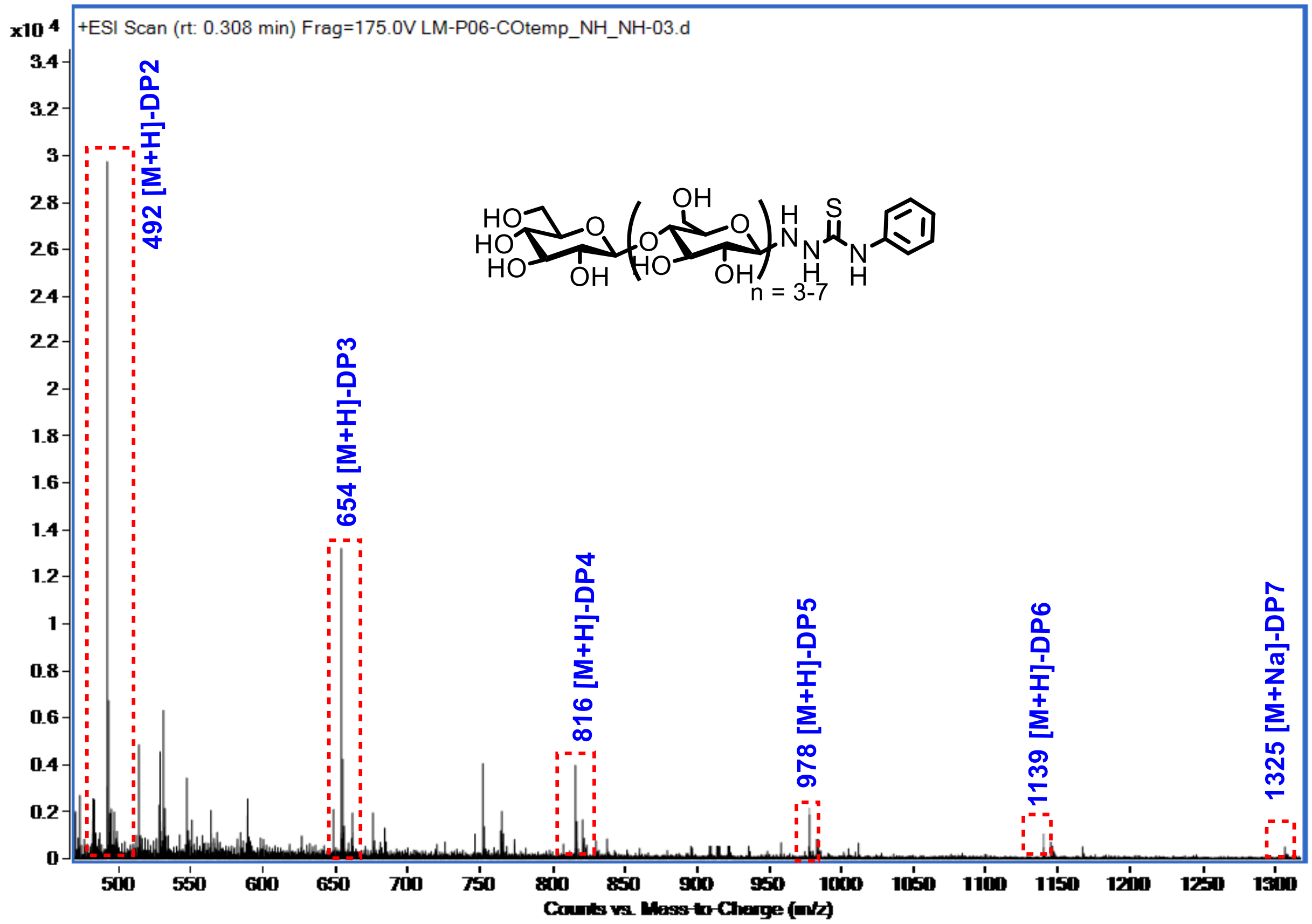

+ESI Scan (rt: 0.308 min) Frag=175.0V LM-P06-COtemp_NH_NH-03.d
x10 4
492 [M+H]-DP2
654 [M+H]-DP3
816 [M+H]-DP4
978 [M+H]-DP5
1139 [M+H]-DP6
1325 [M+Na]-DP7
n = 3-7
Counts vs. Mass-to-Charge (m/z)

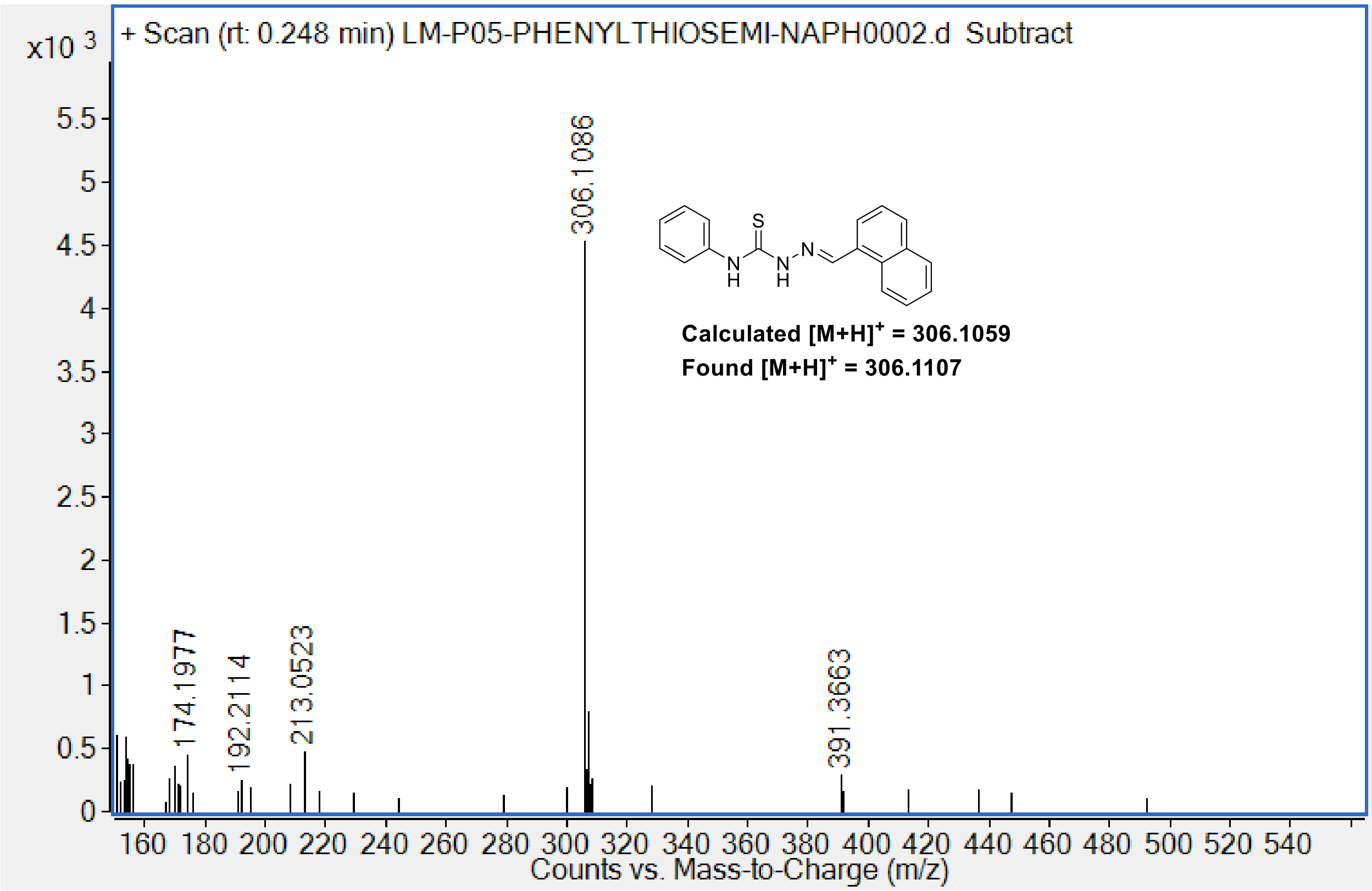
x10 3
+ Scan (rt: 0.248 min) LM-P05-PHENYLTHIOSEMI-NAPH0002.d Subtract
306.1086
174.1977
192.2114
213.0523
391.3663
Calculated [M+H]+ = 306.1059
Found [M+H]+ = 306.1107
Counts vs. Mass-to-Charge (m/z)

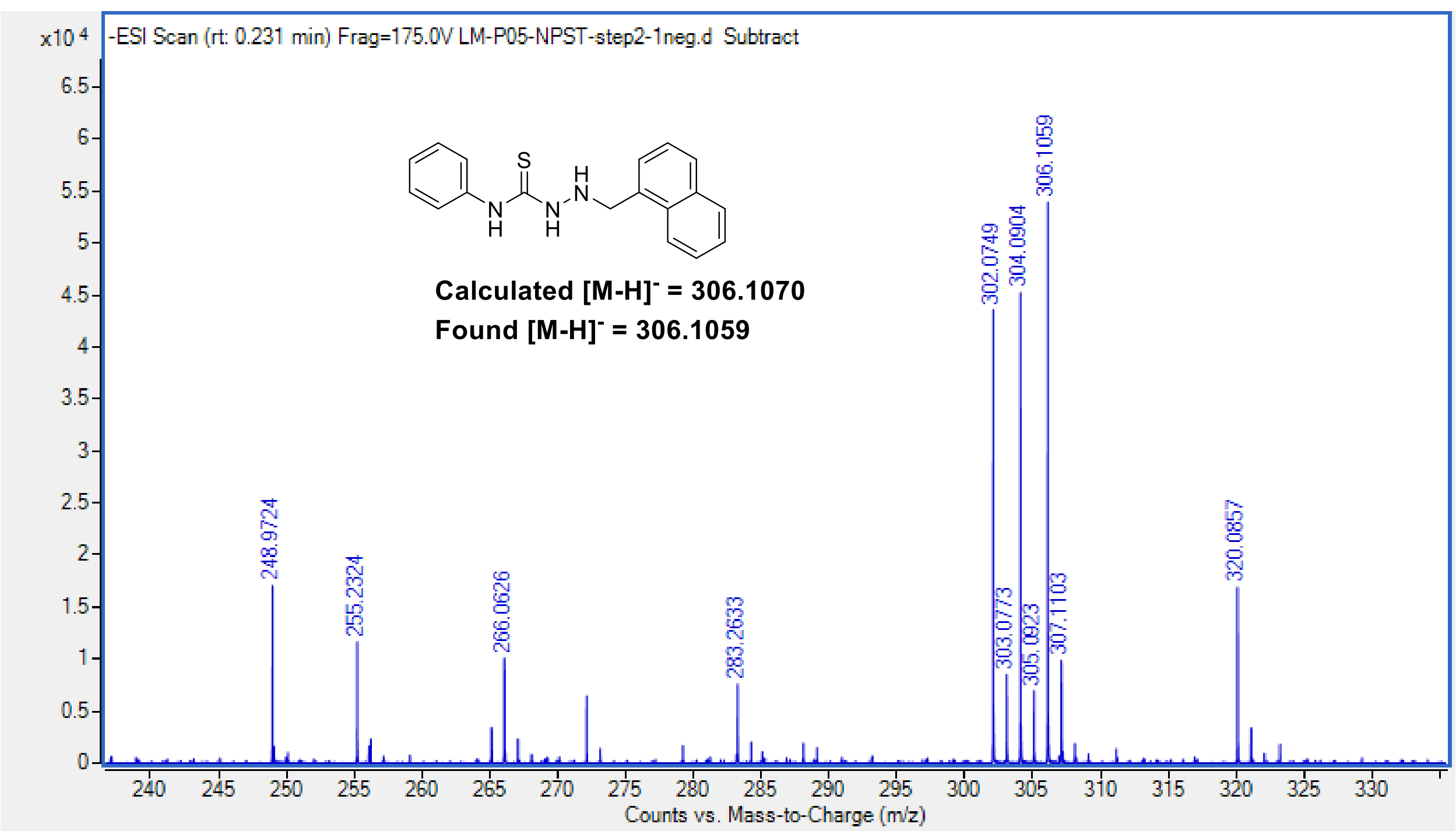
x10 4
-ESI Scan (rt: 0.231 min) Frag=175.0V LM-P05-NPST-step2-1neg.d Subtract
248.9724
255.2324
266.0626
283.2633
302.0749
303.0773
304.0904
305.0923
306.1059
307.1103
320.0857
Calculated [M-H]- = 306.1070
Found [M-H]- = 306.1059
Counts vs. Mass-to-Charge (m/z)

References

(1) Sravan Kumar, J.; Alam, M. A.; Gurrapu, S.; Nelson, G.; Williams, M.; Corsello, M. A.; Johnson, J. L.; Jonnalagadda, S. C.; Mereddy, V. R. Synthesis and Biological Evaluation of Novel Benzoxaboroles as Potential Antimicrobial and Anticancer Agents. *J. Heterocycl. Chem.* **2013**, *50*, 814-820.
(2) Gunasekara, R. W.; Zhao, Y. Correction to "A General Method for Selective Recognition of Monosaccharides and Oligosaccharides in Water". *J. Am. Chem. Soc.* **2025**, *147*, 15915-15915.
(3) Awino, J. K.; Zhao, Y. Protein-Mimetic, Molecularly Imprinted Nanoparticles for Selective Binding of Bile Salt Derivatives in Water. *J. Am. Chem. Soc.* **2013**, *135*, 12552-12555.
(4) (a) Giguère, D.; Sato, S.; St-Pierre, C.; Sirois, S.; Roy, R. *Bioorg. Med. Chem. Lett.* **2006***, 16,* 123-126. (b) de Lorenzo, S.; Pillet, L.; Lim, D.; Paradisi, F. *Org. Biomol. Chem.*, **2023**, 21 , 6356-6359. (c) Qiua, X.; Fairbanks, A. J. *Org. Biomol. Chem.*, **2020**, 18, 7355-7365.
(5) Saphier S, Haft A, Margel S. Bacterial reduction as means for colonic drug delivery: can other chemical groups provide an alternative to the azo bond. *J. Med. Chem*. **2012**, 55, 23, 10781–10785.
(6) Sathisha MP, Budagumpi S, Kulkarni NV, Kurdekar GS, Revankar VK, Pai KS. Synthesis, structure, electrochemistry and spectral characterization of (D-glucopyranose)-4-phenylthiosemicarbazide metal complexes and their antitumor activity against Ehrlich ascites carcinoma in Swiss albino mice. *Eur J Med Chem.* **2010**, *45*, 106-13.
(7) Erickson, H. P. Size and shape of protein molecules at the nanometer level determined by sedimentation, gel filtration, and electron microscopy. *Biol. Proced. Online* **2009**, *11*, 32-51.
(8) Wiseman, T., Williston, S., Brandts, J. F. & Lin, L. N. Rapid Measurement of Binding Constants and Heats of Binding Using a New Titration Calorimeter. *Anal. Biochem.* **1989**, *179*, 131-137.
(9) Jelesarov, I. & Bosshard, H. R. Isothermal titration calorimetry and differential scanning calorimetry as complementary tools to investigate the energetics of biomolecular recognition. *J. Mol. Recognit.* **1999**, *12*, 3-18 ().
(10) Velazquez-Campoy, A., Leavitt, S. A. & Freire, E. Characterization of protein-protein interactions by isothermal titration calorimetry. *Methods Mol. Biol.* **2004**, *261*, 35-54.
